# Molecularly Defined Glycocalyx Models Reveal AB_5_ Toxins Recognize Their Target Glycans Superselectively

**DOI:** 10.1021/jacsau.5c00305

**Published:** 2025-05-20

**Authors:** Laia Saltor Núñez, Vajinder Kumar, James F. Ross, Jonathan P. Dolan, Sumitra Srimasorn, Xiaoli Zhang, Ralf P. Richter, W. Bruce Turnbull

**Affiliations:** † School of Chemistry, 4468 University of Leeds, Leeds LS2 9JT, U.K.; ‡ Astbury Centre for Structural Molecular Biology, University of Leeds, Leeds LS2 9JT, U.K.; § School of Molecular and Cellular Biology, University of Leeds, Leeds LS2 9JT, U.K.; ∥ School of Biomedical Sciences and School of Physics and Astronomy, University of Leeds, Leeds LS2 9JT, U.K.; ⊥ Bragg Centre for Materials Research, University of Leeds, Leeds LS2 9JT, U.K.

**Keywords:** synthetic glycocalyx, biomimetic interfaces, lectin binding, glycoconjugate, superselectivity, QCM-D

## Abstract

AB_5_ toxins are a class of bacterial toxins that recognize cell surface carbohydrates to facilitate their uptake by the target cell. Among them are cholera toxin (CT) from Vibrio cholerae that causes cholera, and Shiga toxin (STx) from Shigella dysenteriae and certain strains of Escherichia coli, which cause hemolytic uremic syndrome. While the glycolipid ligands for CT and STx (gangliosides GM1 and Gb_3_, respectively) have long been known, recent studies have shown that fucosylated structures, like Lewis^*x*^ (Le^*x*^), also play a role in CT binding. This realization raises questions about the importance of interactions between these toxins and nonglycolipid components of the glycocalyx, which are not well understood. To address this challenge, we created glycocalyx models of defined thickness and tunable molecular composition through grafting of mucin-like glycopolymers on solid-supported lipid bilayers (SLBs). The synthesized mucin-like glycopolymers comprised a hyaluronic acid (HA) backbone, an anchor tag (biotin or hexa-histidine) at the HA reducing end, and side chains of relevant oligosaccharides (Le^*x*^, Gb_3_, or lactose) at defined densities. Analyses by quartz crystal microbalance with dissipation monitoring and spectroscopic ellipsometry provided quantification of the thickness, mesh size, and target glycan concentration of the glycocalyx models and of toxin binding kinetics. The B subunit pentamers of both CT and STx showed significantly enhanced affinity in the model glycocalyx environment due to multivalent binding to their respective target glycans. Most notably, toxin binding increased superlinearly with the concentration of the target glycan in the model glycocalyx. We propose that such “superselective” binding is an important factor in host cell selection. Our approach provides a new set of tools to make designer glycocalyces and analyze multivalent protein-glycan interactions in a controlled environment.

## Graphical abstract

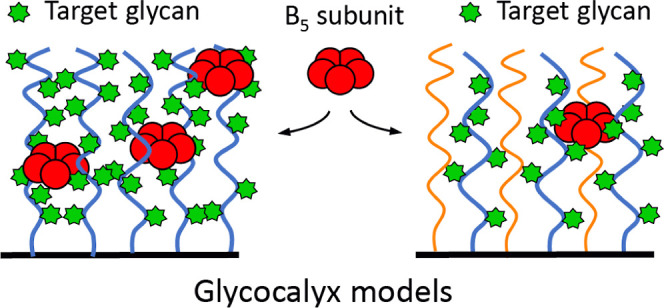

The glycocalyx is a carbohydrate layer on the cell surface, presenting glycolipids, glycoproteins, proteoglycans, and glycosaminoglycans (GAGs).^1,2^ This interface performs important cellular functions, including protecting the cell from external pathogenic agents (e.g., toxins and viruses) and mediation of communication between cells.^3,4^ Moreover, the glycocalyx is also the target of diverse glycan binding proteins (lectins).^5–7^ The high diversity and complex disposition of carbohydrate structures in this layer make it challenging to study the interactions of the glycocalyx with viruses and lectins. Therefore, several groups have sought to build better-defined models that reproduce selected properties of the glycocalyx, ranging from simple arrays of glycoconjugates attached to a surface,^8–15^ to more complex systems in which lipid-linked glycans are presented in fluid layers such as supported lipid bilayers (SLBs) or giant unilamellar vesicles (GUVs) that mimic the cell membrane.^16,17^

Several groups have described the synthesis and application of structures mimicking mucin proteins to add a third dimension to their models and increase their similarity to the extracellular matrix.^18–24^ These glycopolymers typically comprise monosaccharides or oligosaccharides attached to a polymer backbone, an anchor at one end (e.g., a lipid or covalent bond to a surface), and sometimes a fluorophore at the other end or along the chain. They have been incorporated, for example, into SLBs^20^ and arrays^21^ to study binding with Influenza A viruses and introduced onto red blood cell membranes^18^ to study interactions with Concanavalin A and Sambucus nigra agglutinin.

We and others have also described films made from GAGs, with thicknesses ranging from tens of nm to μm, as models of the glycocalyx and glycan-rich extracellular matrix.^25–28^ A common approach to achieve such films is by incorporating biotin at the reducing ends of hyaluronic acid (HA) or sulfated GAGs as an anchor for their attachment on a surface. The biophysical properties of the resulting films were studied in detail, including surface density, thickness, elasticity, and porosity. Such films have proven versatile to study how GAG-binding proteins (e.g., chemokines,^29^ growth factors, and TSG-6 ^25^) and proteoglycans (e.g., aggrecan^26^) bind to GAG films and modulate their biophysical properties.

In the present work, we have established glycocalyx models to investigate the binding interactions of an important class of lectins that can interact with multiple components of the glycocalyx: the AB_5_ bacterial toxin family of proteins that is responsible for several diarrheal diseases.^30,31^ AB_5_ toxins have a quaternary structure consisting of 5 subunits of a glycan-binding protein that arrange into a doughnut-shaped pentamer (B_5_) and an A-subunit that is enzymatically active and toxic to the host cell (Figure 1A,B). A prominent example of AB_5_ toxins is cholera toxin (CT), secreted by Vibrio cholerae, which is the cause of life-threatening diarrhea in the world’s longest pandemic.^32^ To recognize and enter its intestinal epithelial and endothelial host cells, CT first binds the cell glycocalyx through the B-subunit (CTB). CTB has two sets of binding sites that recognize distinct glycans: the canonical binding site is located on the base of the protein and recognizes the oligosaccharide portion of ganglioside GM_1_ with high specificity and affinity;^33,34^ the noncanonical binding site is located on the lateral face of CTB and binds more weakly, with *K*_d_ in the millimolar range, to histo-blood group antigens Lewis^*y*^ (Le^*y*^) and Lewis^*x*^ (Le^*x*^) (Figure 1A).^35–39^ On the other hand, Shiga toxin (STx) is an AB_5_ toxin secreted by Shigella dysenteriae and some strains of Escherichia coli, such as O157:H7.^40,41^ Infection causes food poisoning, resulting in abdominal pain, watery diarrhea, hemorrhagic colitis, and hemolytic uremic syndrome.^42–45^ The B-subunit pentamer of Shiga Toxin (STxB) has three sets of glycan-binding sites per protomer (total 3 × 5 = 15) located on the base of the protein,^40,46,47^ all of which recognize the glycosphingolipid Gb_3_ (and in some protein subtypes, also the glycosphingolipid Gb_4_). While the multivalent interaction has a *K*_d_ in the nanomolar range, individual Gb3 oligosaccharides bind with varying affinity from 1.5 to >15 mM depending on the binding site (Figure 1B).^48–51^

**Figure 1:**
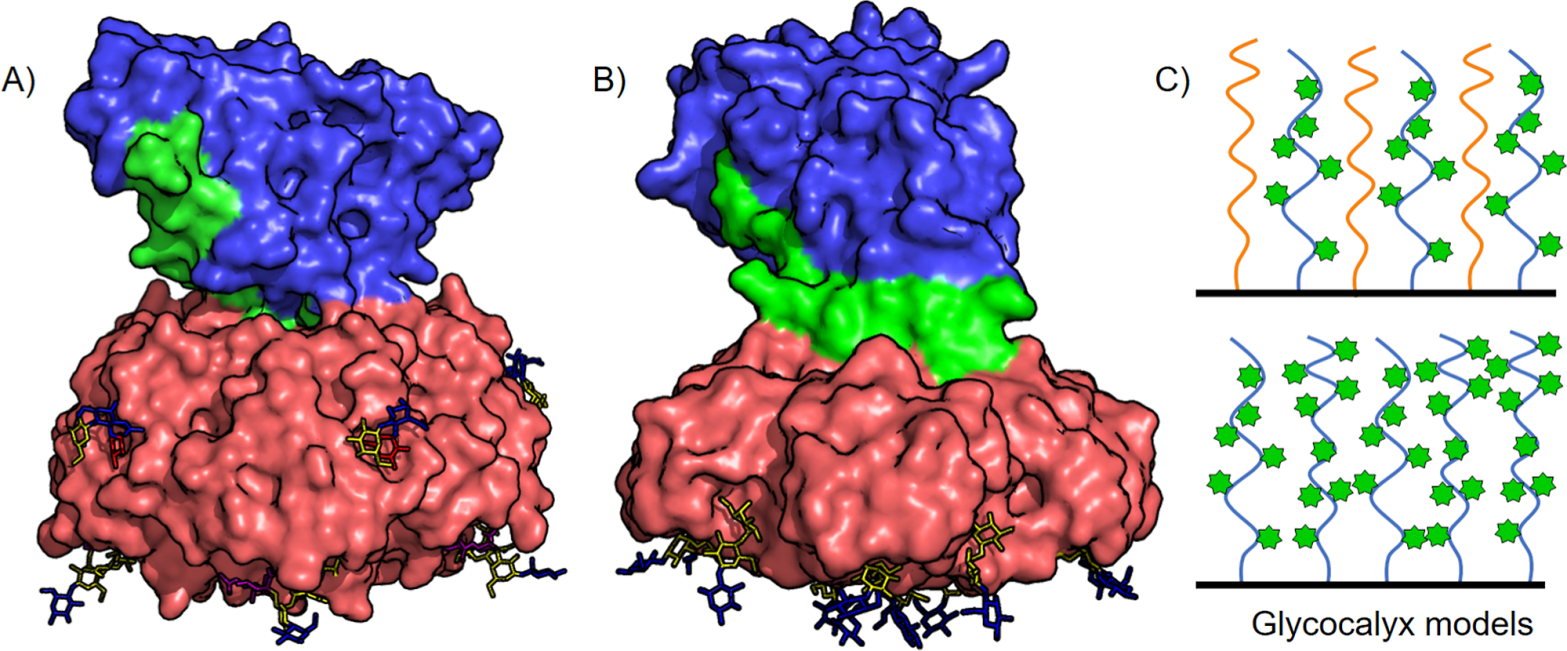
Models of (A) cholera toxin and (B) Shiga toxin bound to their carbohydrate ligands. The model of cholera toxin bound to GM1 (bottom face) and Lewis^*x*^ (lateral face) is based on Protein Data Bank files 3CHB, 1XTC, and 6HJD. The model of Shiga toxin bound to Gb_3_ oligosaccharide (bottom face) is based on Protein Data Bank files 1BOS and 1DM0. In each case, the B_5_ subunit is colored red, the A1 toxin domain is colored blue, and the A2 linker peptide is colored green. The oligosaccharides are shown as stick representations in the colors corresponding to the symbolic nomenclature for glycans: glucose and *N*-acetylglucosamine in blue; galactose and *N*-acetylgalactosamine in yellow; fucose in red; and sialic acid in purple. (C) Schematic representation of a glycocalyx model with tunable target glycan density to analyze multivalent binding of B_5_ subunits in molecularly defined microenvironments. The glycan (represented as a green star) density is modified by mixing different mucin-like structures (top) up to saturating the surface with one type of glycopolymer (bottom).

An open question currently is how the multiple binding sites on AB_5_ toxins conspire for selective recognition of their host cells and to facilitate cell entry. The arrangement of the glycolipid-binding sites on the pentameric faces of CTB and STxB appear optimal for interaction with multiple glycolipids in the cell membrane, but what about other potential interactions that could occur higher in the glycocalyx? The arrangement of the lower affinity Le^*x*^- or Le^*y*^-binding sites around the periphery of CTB might be better disposed for multivalent interactions with a 3D arrangement of glycans attached to glycoproteins. But could having similarly low affinity binding sites arranged on the flat surface of STxB also allow efficient multivalent interactions with a 3D glycocalyx? How strongly does the binding of AB_5_ toxins depend on the density of their glycan binders in the glycocalyx? To address these questions, we describe the synthesis of glycopolymers based on an HA backbone with mucin-like densities of pendant glycans and with a defined degree of substitution (*DS*). We incorporated a biotin (and, alternatively, a polyhistidine) anchorage tag at one end of the polymers to attach them to a surface for the construction of glycocalyx models with defined composition and molecular organization­(Figure 1C). After characterization on a surface, we demonstrate how such glycocalyx models can be used to quantify the dependence of CTB and STxB binding on the concentration of their respective target glycan. Specifically, we reveal a superlinear dependence of multivalent binding on ligand concentration, a phenomenon that has been termed “superselectivity”.^52^

## Results

### Design and Synthesis of Mucin-Like Glycopolymers

HA was chosen as the polymer backbone because the physical properties of films of plain HA polysaccharides grafted to a surface have been studied extensively.^29,53,54^ HA is very soluble under physiological conditions, and its negative charge and large persistence length (4 nm)^55^ facilitate the formation of relatively thick films at comparatively low grafting densities.^56,57^ The charge state of HA also reproduces the dominance of negative charges in glycocalyces, typically imparted through GAGs and sialylated glycoconjugates such as mucins.^4^

HA has an alternating sequence of β-linked *N*-acetylglucosamine (GlcNAc) and glucuronic acid (GlcA) residues, the latter of which can be used for derivatization of the polymer with pendant amide groups while controlling the *DS*.^58,59^ Introduction of an alkyne through amide bonds on HA has been described^60–63^ and allows copper-catalyzed azide alkyne cycloaddition (CuAAC) chemistry to incorporate desired pendant groups. Furthermore, the introduction of azides into oligosaccharides is also well-known.^64–66^ Moreover, the hemiacetal group at the reducing terminus of HA allows chemical modification at a single position by oxime ligation to attach an anchor (e.g., biotin) for grafting the structures to a surface.^53,54^

Lewis^*x*^ (Le^*x*^) and Gb_3_ trisaccharides and lactose (Lac) disaccharides were chosen as glycan moieties for the synthesis of well-defined mucin-like glycopolymers. Le^*x*^ and Gb_3_ were selected for their affinity to CTB and STxB, respectively, and Lac as a convenient control. The first step was the synthesis of the oligosaccharides with a pendant azide group (Figure 2).

**Figure 2:**
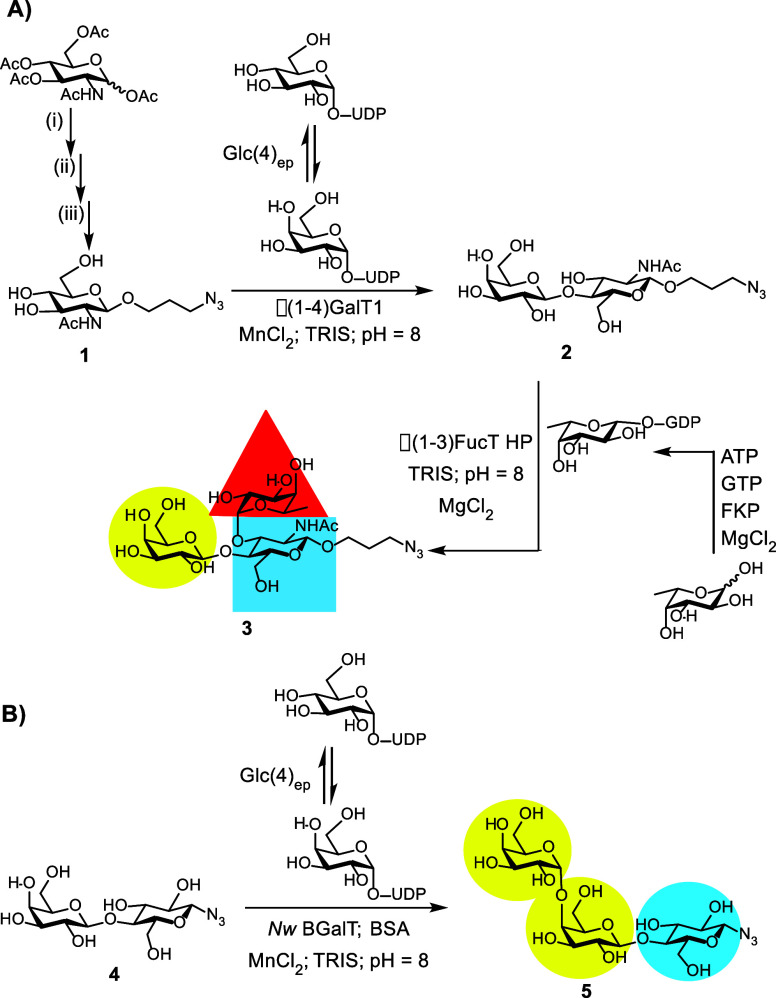
(A) Chemoenzymatic synthesis of azidopropyl Le^*x*^-N_3_: (i) trimethylsilyl trifluoromethanesulfonate (TMSOTf), CH_2_Cl_2_, r.t, 2.5 h, 90%; (ii) 3-azidopropan-1-ol, camphorsulfonic acid, DCE, 80 °C, overnight, 30%; and (iii) sodium methoxide, MeOH, r.t., 3 h, 68%. (B) Enzymatic synthesis of Gb_3_-N_3_.

Azidopropyl Le^*x*^ (Le^*x*^-N_3_, **3**) was synthesized in two stages, starting with chemical attachment of an azide group into GlcNAc, followed by enzymatic synthesis of Le^*x*^ (Figure 2A). Per-acetylated GlcNAc was converted to an oxazoline using TMSOTf and used to glycosylate azidopropanol in the presence of camphorsulfonic acid to give the β-glycoside product.^64,65^ Deprotection of the hydroxyl groups using sodium methoxide in methanol provided azidopropyl GlcNAc **1**. Enzymatic synthesis of Le^*x*^-N_3_ was performed in a one pot, two-step process. First, 3-azidopropyl *N*-acetyllactosamine **2** was made using two enzymes: UDP-Glc-4-epimerase (Glc(4)_ep_)^67^ converted uridine diphosphate glucose (UDP-Glc) into uridine diphosphate galactose (UDP-Gal) in situ for Homo sapiens β-1,4-galactosyltransferase (β(1–4)­GalT1) to glycosylate azidopropyl GlcNAc acceptor **1**.^68,69^ The crude reaction mixture was then used directly for the synthesis of **3**. Guanosine 5′-diphospho-β-l-fucose (GDP-Fuc) was synthesized using l-fucose (Fuc), using adenosine triphosphate (ATP) and guanosine-5′-triphosphate (GTP) as substrates for Bacteroides fragilis GDP-Fuc pyrophosphorylase (FKP).^70^ Finally, the reaction of GDP-Fuc with **2** was catalyzed by Helicobacter pylori α-1,3-fucosyltransferase (α(1–3)­FucT HP)^71^ to achieve Le^*x*^-N_3_.^68,69^ Synthesis of azido Gb_3_ **5** was performed as reported previously,^72^ with in situ generation of UDP-Gal, and Neisseria weaveri α­(1,4)­galactosyltransferase (*N*_w_ GalT)^73^ to glycosylate azido Lac **4** (Figure 2B).^66^

Alkyne-substituted HA has been prepared previously in the presence of EDC and NHS as activators in slightly acidic media (MES buffer at pH 6).^60–63^ However, our initial attempts to follow this method led to products that displayed a variety of additional signals in their NMR spectra that indicated other moieties derived from the coupling agents had become attached to the HA backbone, and could not be removed after multiple rounds of purification (Figure S1A).^60–63^ 4-(4,6-Dimethoxy-1,3,5-triazin-2-yl)-4-methylmorpholinium chloride (DMTMM) has been described as an alternative to EDC/NHS for the formation of amides in monosaccharides and polysaccharides.^59,74–76^ The conditions described by Yu et al.^75^ were adapted to attach propargylamine, and we obtained clean conversion to HA-*g*-propargyl **6** (Figures 3A and S1B). The number of equivalents of propargylamine and DMTMM was varied to provide HA-*g*-propargyl with different *DS* (Table S1).

**Figure 3:**
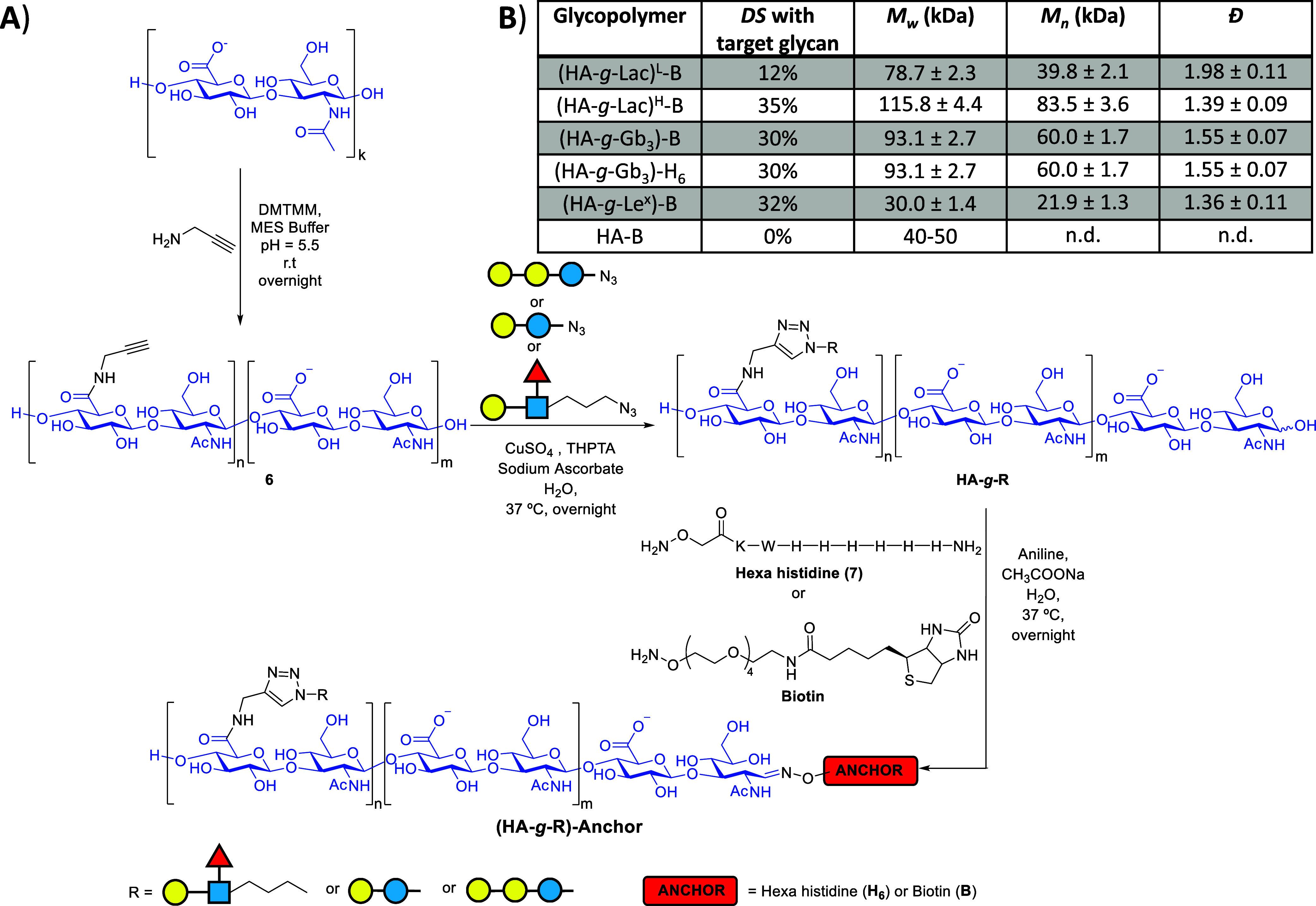
A) Synthesis of **(HA-** *g* **-R)-Anchor** glycopolymers as mucin-like structures. R = Le^*x*^, Gb_3_, or Lac, and anchor = biotin (B) or hexa-histidine (H_6_), as schematically shown. (B) Table of the mucin-like structures synthesized, with their physical properties. Degree of substitution (*DS*) with R per HA disaccharide was determined by ^1^H NMR; weight-average molecular mass (*M*_w_), number-average molecular mass (*M*_*n*_), and dispersity (*D̵* = *M*_w_/*M*_n_) were determined by SEC-MALS; see Methods for details.

HA-*g*-propargyl **6**, with the highest and lowest *DS*, was conjugated to Lac-N_3_ **4** using CuAAC in the presence of CuSO_4_, sodium ascorbate, and tris­((1-hydroxy-propyl-1*H*-1,2,3-triazol-4-yl)­methyl)­amine (THPTA)^77^ to give glycopolymers **HA-** *g* **-Lac** ^**L**^ and **HA-** *g* **-Lac** ^**H**^, where the superscripts ^L^ and ^H^ denote their comparatively low and high *DS*, respectively (Figure 3B). ^1^H NMR spectroscopy showed no unreacted alkyne remaining, and comparison of the integrations of the HA acetamide signal, the triazole proton in the aromatic region of the spectrum, and the anomeric proton from glucose at 5.11 ppm indicated the *DS* was 12% for **HA-** *g* **-Lac** ^**L**^ and 35% for **HA-** *g* **-Lac** ^**H**^. HA-*g*-propargyl **6**, with the highest *DS*, was also coupled to Le^*x*^-N_3_ **3** and Gb_3_-N_3_ **5**, under the same conditions to give glycopolymers with comparable *DS* (30% for **HA-** *g* **-Gb**_**3**_ and 32% for **HA-** *g* **-**Le^*x*^). In these cases, a small alkyne signal was still visible in the ^1^H NMR spectra of the final glycopolymers, but as each glycopolymer had similar densities of glycans to **HA-** *g* **-Lac** ^**H**^, we concluded that the incomplete cycloaddition reactions would have no impact on our subsequent experiments. We note that the *DS* values estimated for HA-*g*-propargyl samples were consistently higher than those for the corresponding HA-*g*-glycans. However, as the products of CuAAC reactions presented more distinct ^1^H NMR signals for comparison, their integration was more reliable than for HA-*g*-alkyne, thus giving better estimation of *DS*.

Soltes et al. have reported that treating HA with copper­(II) salts and ascorbate can result in some degradation of HA,^78^ and we also observed that the size of these glycopolymers (as analyzed by size exclusion chromatography multi angle light scattering (SEC-MALS)) decreased during the CuAAC reaction, albeit to varying degrees. Starting from a number-average molecular mass *M*_*n*_ of 137 kDa for HA-*g*-propargyl (Table S2), the CuAAC reactions provided glycopolymers with *M*_*n*_ = 22 kDa for **HA-** *g* **-**Le^*x*^, 60 kDa for **HA-** *g* **-Gb**_**3**_, 40 kDa for **HA-** *g* **-Lac** ^**L**^, and 84 kDa for **HA-** *g* **-Lac** ^**H**^ (Figure 3B and Table S2).

Finally, the reducing end of each HA-*g*-R glycopolymer was modified to allow its anchorage at a surface. Oxime ligation of HA-*g*-R and alkoxyamine-(ethylene glycol)_4_-biotin, using aniline as a nucleophilic catalyst at pH 7,^53,54^ provided **(HA-** *g* **-**Le^*x*^ **)-B**, **(HA-** *g* **-Gb**_**3**_ **)-B**, **(HA-** *g* **-Lac** ^**L**^ **)-B**, and **(HA-** *g* **-Lac** ^**H**^ **)-B** (Figure 3). The same procedure was also performed on underivatized HA (*M*_*w*_ = 40–50 kDa) to synthesize HA-biotin (**HA-B**) as a noninteracting building block for the construction of glycocalyx models (Figure 3B). In addition, a peptide with a hexa-histidine sequence and a terminal alkoxyamine (**7**) was made by solid-phase peptide synthesis and attached to the reducing end of **HA-** *g* **-Gb**_**3**_ by oxime ligation, giving **(HA-** *g* **-Gb**_**3**_ **)-H**_**6**_ (Figure 3). Successful terminal modification of the mucin-like glycopolymers was confirmed by quartz crystal microbalance with dissipation monitoring (QCM-D) during construction of the glycocalyx models on surfaces presenting either streptavidin (SAv, for biotin capture) or Ni^2+^-nitrilo triacetic acid (NTA) moieties (for histidine capture; vide infra).

### Preparation of Molecularly Defined Glycocalyx Models

The self-organization mechanism and final architecture of our model glycocalyces are shown schematically in Figure 4A. SLBs (Figure 4A ①) were formed by the method of vesicle spreading,^79^ and reproduce salient properties of the cell membrane, notably the lipid bilayer organization and fluidity allowing diffusion of lipids and attached proteins and/or glycopolymers in the membrane plane. The lipid composition can be readily varied to build desired functions into SLBs. In our case, the SLBs contained mostly phospholipid DOPC to provide a background that is resistant to nonspecific binding of most proteins, along with a small fraction of synthetic lipids designed to attach the mucin-like glycopolymers via their biotin. A SAv monolayer was added (Figure 4A ②) to link the biotin on mucin-like glycopolymers to biotin-presenting lipids. In solution and when surface-anchored at low coverage (Figure 4A ③), the mucin-like glycopolymers are expected to form random coils; as the surface coverage increases, the individual molecules will repel each other and entail stretching of the HA backbone and formation of a “brush” morphology (Figure 4A ④).

**Figure 4:**
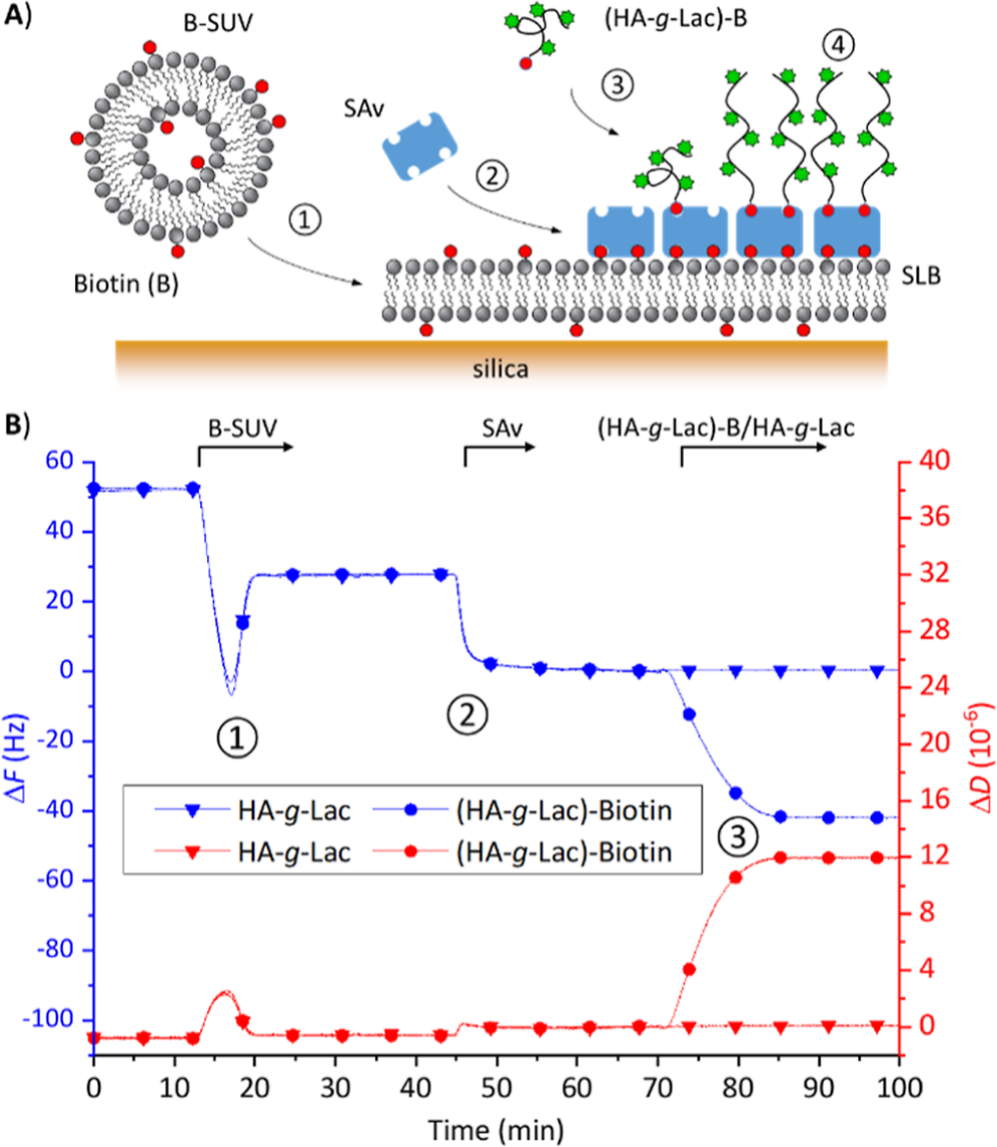
(A) Scheme for the supramolecular self-organization process to form glycocalyx models: (1) adsorption of small unilamellar vesicles containing biotinylated lipids (B-SUVs) on the silica surface, and their subsequent rupture to form a supported lipid bilayer (SLB); (2) binding of streptavidin (SAv) by at least two biotins on the SLB to form a SAv monolayer; (3) anchorage of the biotinylated mucin-like glycopolymer and formation of a glycopolymer brush. (B) Quartz crystal microbalance with dissipation monitoring (QCM-D) data showing frequency shift (Δ*F*), dissipation shift (Δ*D*; overtone *i* = 5) demonstrating stable and specific anchorage of **(HA-** *g* **-Lac** ^**L**^ **)-B** via its biotin on a SAv-on-SLB surface. Conditions: B-SUVs (DOPC/DOPE-CAP-B 95:5 (mol/mol), 50 μg/mL), SAv (20 μg/mL), **(HA-** *g* **-Lac** ^**L**^ **)-B**/**HA-** *g* **-Lac** ^**L**^ (20 μg/mL); all solutions were prepared in working buffer (HBS; HEPES 10 mM, NaCl 150 mM, pH 7.4). Arrows atop the graph indicate the start and duration of incubation with each sample as indicated; during remaining times, plain working buffer was flowed over the sensor surface.

QCM-D was used to monitor the assembly of glycocalyx models. QCM-D is sensitive to the mass/thickness and mechanical properties of surface adlayers. To a first approximation, a negative shift in resonance frequency (Δ*F*) relates to an increase in mass (including hydrodynamically coupled solvent), and a positive dissipation shift (Δ*D*) is a measure of adlayer softness. QCM-D data in Figure 4B are for the formation of a **(HA-** *g* **-Lac** ^**L**^ **)-B** brush. The biphasic response upon exposure of small unilamellar vesicles (SUVs) to the QCM-D sensor surface (Figure 4B; 13 to 23 min) is characteristic of SUVs initially binding intact, followed by their rupture and coalescence into a SLB. The extrema in Δ*D* and Δ*F* here arise from the SUV layer being softer and trapping more solvent, respectively, than the final SLB.^79^ The net frequency shift at the end of SUV exposure (Δ*F* = −25 ± 1 Hz) reveals a film thickness of 4.5 nm, as expected for a hydrated lipid bilayer, and the close-to-zero net dissipation shift (Δ*D* < 0.5 × 10^–6^) indicates the SLB is of good quality (i.e., with minimal residual surface-bound SUVs).^79^ Exposure to SAv (Figure 4B; 45 to 55 min) led to a further decrease in frequency (Δ*F* = −24 ± 1 Hz) and a relatively small increase in dissipation (Δ*D* = 0.6 × 10^–6^), consistent with the formation of a protein monolayer of ∼4 nm thickness. Indeed, with 5 mol % of biotin-presenting lipids in the SLB, a dense monolayer of SAv is expected to form.^29^

Binding was clearly observed when **(HA-** *g* **-Lac** ^**L**^ **)-B** was flowed over the SAv-on-SLB surface, whereas there was no measurable response for **HA-** *g* **-Lac** ^**L**^ (Figure 4B; 72 to 88 min; lines with circle and triangle symbols, respectively). This demonstrated specific anchorage of the mucin-like structure via its biotin tag. The responses for **(HA-** *g* **-Lac** ^**L**^ **)-B** were saturable (Δ*F* = −40 Hz and Δ*D* = 6.8 × 10^–6^) and unchanged upon rinsing with buffer, indicating full occupation and stable binding to the biotin-binding sites on the surface.

Similar experiments with **(HA-** *g* **-**Le^*x*^ **)-B** and **(HA-** *g* **-Gb**_**3**_ **)-B** and their nonbiotinylated precursors demonstrated specific, saturable, and stable anchorage of all these mucin-like structures (Figures S2 and S3). In addition, (Ni^2+^-NTA)_3_-presenting lipids^80^ were incorporated into a bilayer (Figure S4) to capture the hexa-histidine anchor tag at the reducing end of **(HA-** *g* **-Gb**_**3**_ **)-H**_**6**_. The his-tagged glycopolymer could be anchored specifically and stably via its H_6_ tag to the (Ni^2+^-NTA)_3_-presenting SLBs (Figure S5). This illustrates the versatility of our approach to making model glycocalyces.

### Quantification of Model Glycocalyx Thickness, Mesh Size, and Target Glycan Concentration

We deployed in situ spectroscopic ellipsometry (SE) to quantify the surface density of mucin-like structures. SE is sensitive to the thickness and refractive index of surface adlayers and enables label-free quantitation of the biomolecular mass per unit surface area (i.e., the areal mass density, AMD). Brushes of mucin-like glycopolymers were formed as described above for the QCM-D analyses, with all binding steps instead monitored by SE (Figures S6–S8).

Table 1 captures the AMD (determined by SE) and the thickness (*h*) (determined by QCM-D) of the glycopolymer brushes with the most relevant target glycans (i.e., Gb_3_ for STxB and Le^*x*^ for CTB). The brush thicknesses (15 to 20 nm) exceed the hydrodynamic diameter of the toxin B_5_ molecules (∼5.5 nm)^81,82^ by several fold, indicating that the toxin can fully immerse and will experience a three-dimensional glycan environment within the brush.

**Table 1:** Salient Properties of Brushes of Mucin-Like Structures

**glycopolymer**		**(HA-** *g* **-Gb**_**3**_ **)-H**_**6**_	**(HA-** *g* **-Gb**_**3**_ **)-B**	**(HA-***g***-**Le^*x*^**)-B**
brush	AMD (ng/cm2)	98.7 ± 0.3	68.8 ± 0.2	66.1 ± 0.4
	*h* (nm)	19.6 ± 0.8	15.6 ± 2.2	17.6 ± 0.8
glycopolymer	Γ (pmol/cm^2^)		6.1 ± 0.9	6.6 ± 0.9
	*M*_*n*,anchored_ (kDa)		11.3 ± 1.5	10.0 ± 1.4
	*L*_c,anchored_ (nm)		20.7 ± 2.8	17.1 ± 2.3
	*d*_rms_ (nm)		5.2 ± 0.4	5.0 ± 0.4
target glycan	Γ (pmol/cm^2^)	54.0 ± 0.2	37.6 ± 0.1	36.1 ± 0.2
	*c* (mM)	27.5 ± 1.1	24.1 ± 3.4	20.5 ± 0.9
	*d*_rms_ (nm)	3.9 ± 0.1	4.1 ± 0.2	4.3 ± 0.1

Table 1 also captures salient features of the glycopolymers in the brushes (for biotin-anchored polymers only). The grafting density (Γ) was here assumed to equal the surface-density of available biotin-binding sites on the SAv monolayer (as determined by SE; Figures S7 and S8). From the AMD and Γ, the number-average molar mass (*M*_*n*_,_anchored_) of glycopolymers was determined. It is notable that the average masses of the surface-anchored glycopolymers (Table 1) are lower than the corresponding average masses of the glycopolymers in solution (as determined by SEC-MALS; Figure 3B). Most likely, this is due to the process of surface-grafting preferentially selecting smaller polymer chains, as reported earlier.^83^ Considering, in addition, the *DS* of the glycopolymers with pendant target glycans (Figure 3B), the average contour length of the HA backbone (*L*_c,anchored_) was obtained. That the brush thickness is comparable to, or only marginally smaller than, the contour length (Table 1) implies that the glycopolymer chains are almost fully stretched in the brush environment.

From the grafting density Γ, the root-mean-square distance between anchor sites (*d*_rms_) of the glycopolymers was determined. In well-solvated polymer brushes, the average distance between anchor sites is equivalent to the mean spacing between polymers to a first approximation; that is, *d*_rms_ here represents a measure for the mesh size of the glycopolymer brushes. It can be seen that the mesh size (∼5 nm; Table 1) is comparable to the hydrodynamic diameter of the toxin B_5_ molecules (∼5.5 nm).^81,82^ This implies that any steric constraint imposed by the brush on the movement of toxins is rather moderate.^84^

Table 1 further shows salient features of the target glycans, such as their projected surface density, concentration, and root-mean-square distance, in the brushes. One can see here that target glycan concentrations of several tens of mM are readily achieved and that the average distances between target glycans (∼4 nm; Table 1) are also comparable to the typical distance between binding sites on the toxin molecules (∼3 nm), implying that a toxin should be able to reach multiple target glycans without substantial reorganization of the glycopolymer brush.

As a conclusion, QCM-D and SE jointly provided a detailed physicochemical characterization of the glycocalyx models, including their thickness, mesh size, and concentration of target glycans. These quantities are useful for the design and analysis of lectin binding assays.

### AB_5_ Toxins Specifically Bind Their Target Glycans in Model Glycocalyces

QCM-D was used to test AB_5_ toxin binding to model glycocalyces. Representative data for STxB are shown in Figure 5, and demonstrate that STxB binds selectively, largely reversibly, and in a dose-dependent manner to Gb_3_-containing glycocalyx models (Figure 5; lines with square symbols). Comparatively little binding was seen on SAv-on-SLB-coated surfaces lacking **(HA-** *g* **-Gb**_**3**_ **)-B** (Figure 5; lines with triangle symbols), and there was no response at all for glycocalyx models presenting Lac or Le^*x*^ instead of Gb_3_ (Figure S9). STxB also bound reversibly to **(HA-** *g* **-Gb**_**3**_ **)-H**_**6**_ (Figure S10), whereas there was no direct binding to the (Ni^2+^-NTA)_3_ SLB. These results demonstrated that biotin-anchored and H_6_-anchored mucin-like structures are suitable for interaction studies with this protein.

**Figure 5:**
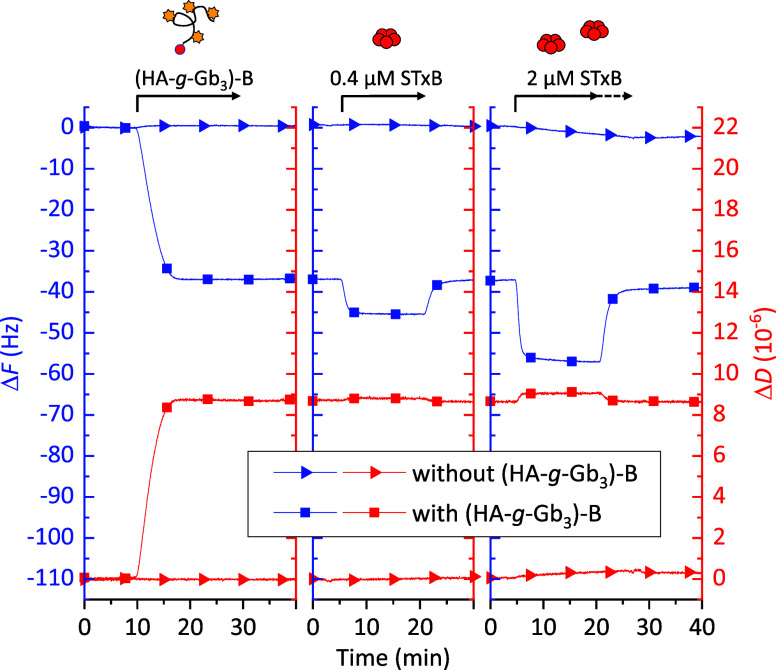
QCM-D data showing frequency shift (Δ*F*), dissipation shift (Δ*D*; overtone *i* = 5) demonstrating specific and largely reversible binding of STxB to a Gb_3_ presenting model glycocalyx. Conditions: SAv-on-SLB surfaces (not shown); **(HA-** *g* **-Gb**_**3**_ **)-B** – 20 μg/mL (lines with square symbols) or none (lines with triangle symbols); STxB (0.4 and 2 μM, as indicated); all in HBS working buffer. Arrows atop the graph indicate the start and duration of incubation with each sample (solid vs dashed arrow for 2 μM STxB with vs without glycopolymer); plain working buffer was flowed over the sensor surface during remaining times.

Analogous experiments with CTB revealed selective and dose-dependent binding to Le^*x*^, and no measurable response for the glycocalyx models presenting Gb_3_ (Figure S11). There was also no or minimal response to the Lac glycocalyx, which probably reflects the very low affinity of CTB for galactosides.^34^ CTB did not bind to bare SAv-on-SLB-coated surfaces (Figure S12), demonstrating that biotin-anchored model glycocalyces are suitable for interaction studies with CTB. CTB did bind to (Ni^2+^-NTA)_3_-presenting SLBs though (Figure S13), most likely due to the histidine residues exposed on the surface of native CTB,^85^ illustrating that the method of anchoring the glycopolymers to the surface is an important consideration for glycocalyx model design.

It is notable that the binding of the B_5_ toxins to glycocalyx models with their respective target glycan generated only very subtle (if any) QCM-D dissipation shifts (Figures 5 and S9–S12). This contrasts with previous work on the binding of chemokines, growth factors, and morphogens to GAG brushes,^86–88^ revealing a rather larger spectrum of dissipation responses ranging from strong decreases to clear increases in dissipation depending on the protein. In these studies, a decrease in dissipation was linked to model glycocalyx rigidification through protein-mediated cross-linking of polysaccharide chains; the lack of such an effect for CTB and STxB here indicates that the rigidification of the target glycan film on multivalent binding of the B_5_ toxins is only moderate.

### Quantification of AB_5_ Toxin Binding Avidities in Molecularly Defined Model Glycocalyces

B-subunit pentamers are expected to interact simultaneously with more than one copy of their target glycan in the glycocalyx. To quantify the aggregate binding strength of such multivalent interactions, we performed protein titration experiments on model glycocalyces by SE. Figure 6A provides representative data for the binding of STxB to a model glycocalyx made from **(HA-** *g* **-Gb**_**3**_ **)-B**. It can be seen that binding reached equilibrium within a few minutes at all concentrations. The unusual transient maxima in binding at the two highest STxB concentrations (2 and 4 μM) are likely due to the scattering of light while the protein mixture in the SE chamber was homogenizing. The vast majority of the protein (∼90%) was rapidly released upon rinsing in plain working buffer, confirming the reversibility of binding already seen by QCM-D (Figure 5).

**Figure 6:**
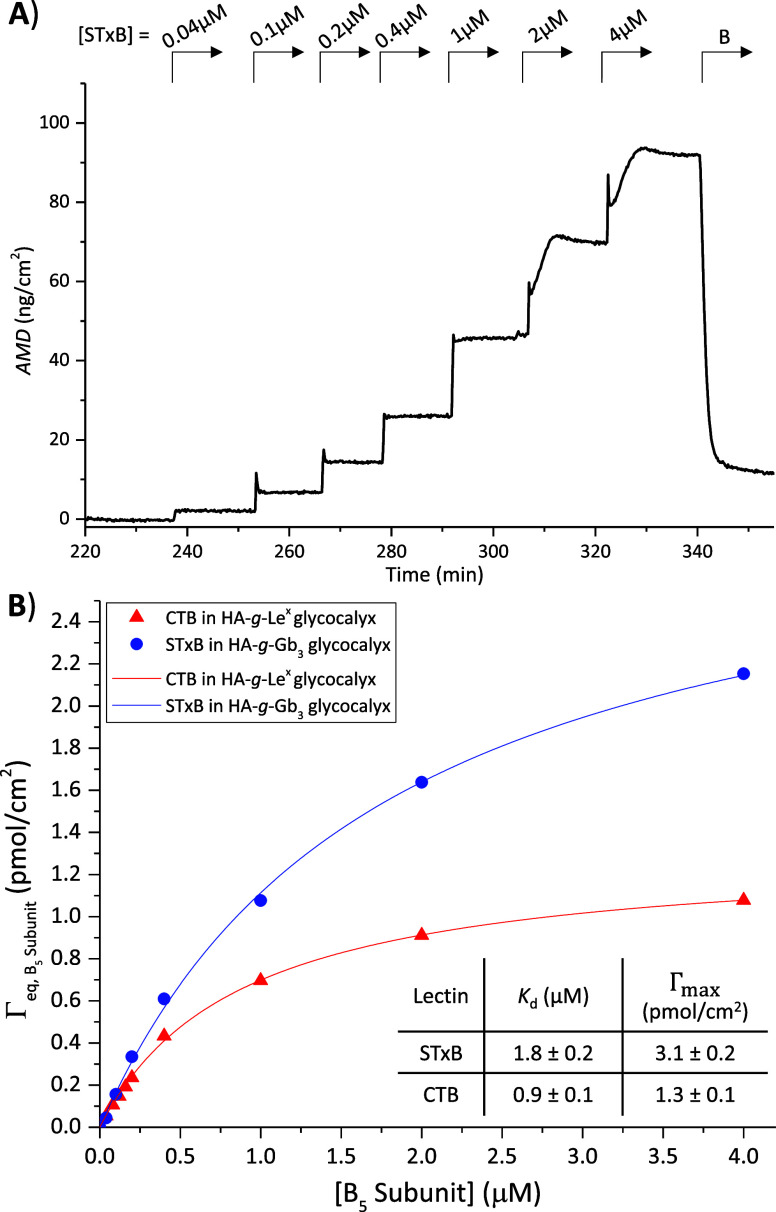
Quantifying B_5_ subunit binding avidities in model glycocalyces. (A) Representative titration curve obtained by SE for STxB in a **(HA-** *g* **-Gb**_**3**_ **)-B** model glycocalyx. (B) Equilibrium B subunit surface densities Γ_eq,B5 subunit_ as a function of the B_5_ subunit concentration for STxB and **HA-** *g* **-Gb**_**3**_ (blue circles), and for CTB and **HA-** *g* **-**Le^*x*^ (red triangles). Lines in corresponding colors are best fits with the Langmuir isotherm, Γ_eq,B5subunit_ = Γ_max,B5subunit_·[B_5_ subunit]/(*K*_d_+[B_5_ subunit]), with results indicated in the table (inset). Data taken from (A) for STxB/Gb_3_ interactions and from Figure S14 for CTB/Le^*x*^ interactions. Conditions: SAv-on-SLB with maximal (HA-*g*-Gb_3_)-B and (HA-*g*-Le^*x*^)-B coverages, corresponding to *c*_Gb3_ = 0.024 M and *c*Le^*x*^ = 0.021 M (Table 1), in HBS working buffer.

The equilibrium binding responses were converted to molar surface densities and are plotted in Figure 6B (blue circles) as a function of the molar protein concentration in the solution phase. CTB titration on a model glycocalyx made from **(HA-** *g* **-**Le^*x*^ **)-B** showed qualitatively comparable features (Figure S14), and the equilibrium binding data are also reported in Figure 6B (red triangles).

In both cases, the data were well-fitted by a Langmuir isotherm (Figure 6B; lines in matching color), which represents the simplest possible interaction model (and effectively neglects the minor fraction of protein binding that is not rapidly reversed). From this analysis, the equilibrium dissociation constant *K*_d_ (here, a measure of binding avidity) and the maximum B5 surface coverage (Γ_max_) were obtained for both lectins (Figure 6B, inset).

The dissociation constant (*K*_d_ = 1.8 ± 0.2 μM) for STxB in the model glycocalyx decreased by approximately 3 orders of magnitude compared to that previously reported for the highest-affinity individual STxB/Gb_3_ oligosaccharide interaction (*K*_d_ = 1.5 ± 0.5 mM).^49,50^ This is clear evidence for the enhanced binding due to multivalency effects in a system that differs from the presentation of Gb_3_ in lipid membranes. Comparison of Γ_STxB,max_ (3.1 ± 0.2 pmol/cm^2^) with the total surface density of Gb_3_ oligosaccharides (37.6 ± 0.1 pmol/cm^2^, Table 1) reveals a glycan/STxB pentamer ratio larger than 10. For CTB, these effects were even more pronounced, with the *K*_d_ decreasing by approximately 11,000-fold (from 10 ± 3 mM for a single CTB/Le^*x*^ binding site^36^ to 0.9 ± 0.1 μM in the model glycocalyx) and a minimal glycan/CTB pentamer ratio of approximately 30 (derived from Γ_CTB,max_ = 1.3 ± 0.1 pmol/cm^2^ and Γ_Le^*x*^_ = 36.1 ± 0.2 pmol/cm^2^ (Table 1)).

### AB_5_ Toxins Recognize Target Glycans Superselectively

The surface density of target glycans in the glycocalyx varies with cell type and state. To model such variations, we mixed glycopolymers bearing the target glycan with plain HA or a glycopolymer with pendant nontarget glycans in the model glycocalyces: to study the effect of **Gb**_**3**_ density on StxB binding, we mixed **(HA-** *g* **- Gb**_**3**_ **)-B** (89.2 kDa) with **(HA-** *g* **-Lac** ^**H**^ **)-B** (114.2 kDa) (Figure 7A, top; Figure S15A); for CTB, we mixed **(HA-** *g* **-**Le^*x*^ **)-B** (29.8 kDa) with **HA-B** (40–50 kDa; lacking pendant glycan moieties) (Figure 7A, bottom; Figure S16A). Sequential incubation of each pair of structures with a tightly controlled (and variable) incubation time for the first structure, afforded good control (and tunability) of the surface density of target glycans. The addition of “inert” glycopolymers of comparable size ensured that the thickness of the model glycocalyx and mesh size remained similar.

**Figure 7:**
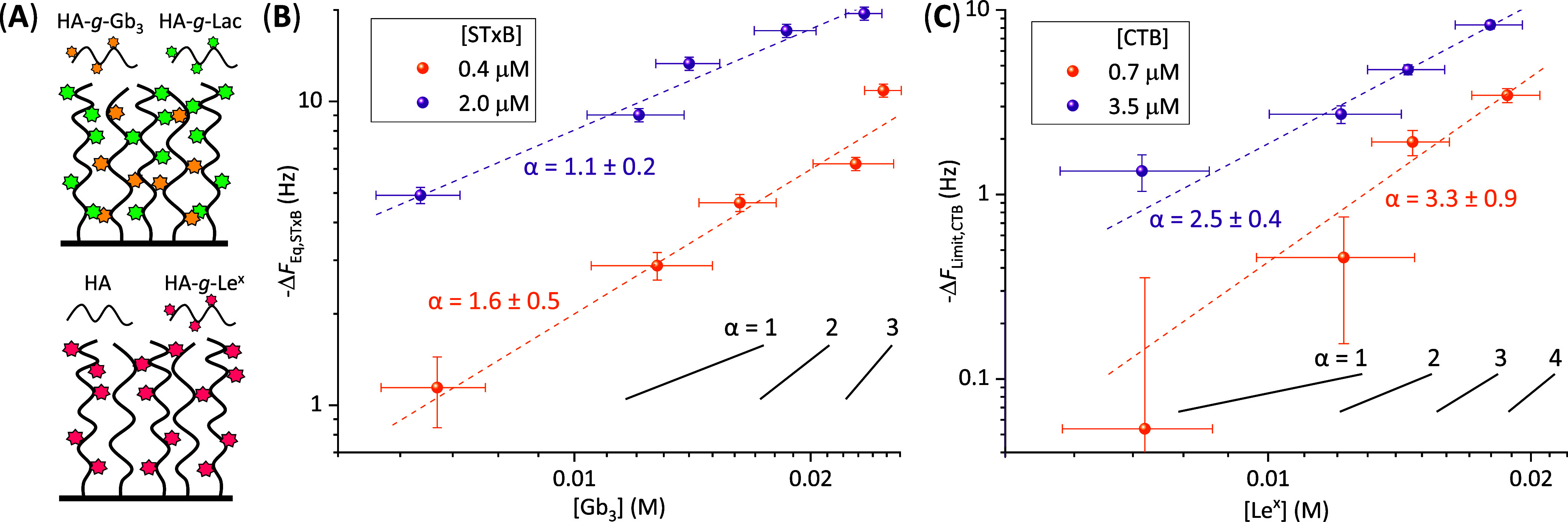
AB_5_ toxins recognize their target glycans superselectively. (A) Schemes of model glycocalyx assemblies deployed to probe for glycan density-dependent binding. Mucin-like structures with the target glycan (Gb_3_ for STxB and Le^*x*^ for CTB) were mixed with structures of similar size representing noninteracting (Lac for STxB) or no (for CTB) glycans other than the HA backbone. (B) Plots of -Δ*F*_eq,STxB_, a measure of STxB binding, against the concentration of Gb_3_ in the model glycocalyx film. Most data are extracted from Figure S15B for 0.4 μM and 2 μM STxB (color coded as indicated), apart from the data points at the highest Gb_3_ concentration, which were derived from the **(HA-** *g* **-Gb**_**3**_ **)-H**_**6**_ data in Figure S10. (C) Plot of -Δ*F*_limit,CTB_, a measure of CTB binding, against the concentration of Le^*x*^ in the model glycocalyx film. Data are extracted from Figure S16B for 0.7 μM and 3.5 μM CTB (color coded as indicated). Dashed lines in matching colors in B and C are power law fits with exponents α (i.e., straight lines in the log–log plots with slope α) as indicated.

Having formed the mixed model glycocalyces, their binding to the bacterial toxins was then quantified by QCM-D (Figures S15B and S16B). Figure 7B,C shows the net negative frequency shifts for binding of STxB and CTB, respectively, as a function of the target glycan concentration in the model glycocalyx. For the STxB experiments, use of the **(HA-** *g* **-Gb**_**3**_ **)-H**_**6**_ with (Ni^2+^-NTA)_3_-presenting SLB allowed access to the highest Gb_3_ concentrations. Each lectin was studied at two concentrations that differed by a factor of 5, within an order of magnitude of the *K*_d_, values obtained in Figure 6. As expected, lectin binding increased monotonically with the concentrations of both the target glycan and the lectin. Most interestingly, the dependence of the binding response on the concentration of target glycans was rather strong. Data in Figure 7B,C are deliberately plotted with logarithmic scales. In this plot, a slope $\alpha =\frac{d\ln\left(-\Delta F_{lectin}\right)}{d\ln c_{glycan}}=\frac{d\left(-\Delta F_{lectin}\right)/-\Delta F_{lectin}}{d\left(c_{glycan}\right)/c_{glycan}}$ larger than one indicates a superlinear increase in the rate of the relative change in lectin binding as a function of the rate of the relative change in target glycan concentration. Crude fits with power laws across the full spectrum of target glycan concentrations reveal mean α values significantly larger than 1 for both CTB concentrations tested and also for 0.4 μM STxB pentamer (Figure 7B,C; dashed lines). For 0.7 μM CTB pentamer, for example, α = 3.3 implies that a 2-fold change in Le^*x*^ concentration entails a 2^3.3^ ≈10-fold change in toxin binding. Such a superlinear dependence of multivalent binding on ligand concentration has been termed “superselectivity”,^52^ and our data thus demonstrate that AB_5_ toxins recognize their target glycans superselectively.

## Discussion

The construction of a library of glycopolymers with mucin-like densities of glycans was successfully achieved by derivatizing HA with propargyl groups and then attaching azide-functionalized glycans by CuAAC. The resulting **HA-** *g* **-Lac** ^**L**^, **HA-** *g* **-Lac** ^**H**^, **HA-** *g* **-Gb**_**3**_, **HA-** *g* **-**Le^*x*^ glycopolymers, and unmodified HA were biotinylated at their reducing termini to allow their assembly into glycocalyx models on a SAv-on-SLB surface. An alternative strategy for anchoring His-tag-functionalized glycopolymers to a (Ni^2+^-NTA)_3_-presenting SLB achieved a higher density of glycans on the surface as a result of the smaller footprint of the His-tag relative to SAv. However, we note that the (Ni^2+^-NTA)_3_-presenting SLB can have the disadvantage of glycan-independent binding to proteins with multiple surface histidine residues and would therefore be incompatible with recombinant lectins having His_6_ purification tags.

Careful structural characterization is essential at each step of the process. The traditional amide coupling agents EDC and NHS are still widely used for derivatizing HA, but we have found these reagents can give rise to poorly defined additional modifications of HA. We would therefore advocate using DMTMM in preference as activator, as this reagent consistently gave very clean amide derivatives. CuAAC is one of the most widely used bioorthogonal reactions, and while it worked well for ligating the polymer and the azide-functionalized glycans, it is important to appreciate that oxidative side reactions can lead to partial fragmentation of the glycopolymers. A comparison of SE (Table 1) and SEC-MALS (Figure 3B) data highlighted further size selection of the glycopolymers upon attachment to the surface. It is thus most important to fully characterize and understand the films ultimately created including the sizes of the brushes, the mesh size, etc.

QCM-D and SE were used to study the binding between STxB or CTB and the different glycocalyx models built using **HA-B**, (**HA-** *g* **-Lac** ^**L**^ **)-B, (HA-** *g* **-Gb**_**3**_ **)-H**_**6**_, **(HA-** *g* **-Gb**_**3**_ **)-B**, or **(HA-** *g* **-**Le^*x*^ **)-B**. These lectins showed selective binding to the brushes containing their target glycans: Le^*x*^ trisaccharide was recognized only by CTB, while films with Gb_3_ bound only to STxB. The STxB-Gb_3_ interaction (*K*_d_ = 1.8 ± 0.2 μM) was enhanced 1000-fold relative to the highest affinity monovalent Gb_3_-oligosaccharide interaction.^49,50^ Such binding enhancements are not uncommon for multivalent systems, including arrayed carbohydrates,^14,15^ yet this is still 500-fold lower affinity than reported for STxB binding to the Gb_3_ glycosphingolipid in a membrane.^89^ This is perhaps not surprising when one considers that the STxB protein architecture has evolved to have all its binding sites on one flat face of the protein, which is optimal for binding to a surface rather than to a 3D structure like our glycocalyx model. Nonetheless, at saturation the Gb_3_/STxB pentamer ratio was >10 (Figure 6), which could be consistent with all of the StxB “site 1”- and “site 2”-binding sites being occupied.^47^ However, other multivalent Gb_3_ ligands only engage the higher affinity “site 2”,^90^ and so the data could also result from only partial saturation of the Gb_3_ ligand groups.

In the case of the Le^*x*^ glycocalyx model binding to CTB, there was a larger 11,000-fold enhancement in binding relative to the 10 mM *K*_d_ reported in the literature for the monovalent interaction.^37^ In this case, the binding sites are arranged around the periphery of the protein, which should be better disposed to multivalent binding to the 3D glycocalyx model. Here, the glycan/CTB pentamer ratio was approximately 30 at saturation, even though it is not feasible for a CTB pentamer to engage with 30 copies of Le^*x*^. Our quantitative analysis (Table 1 and Figures 6 and S14) shows that the combined mass concentration of glycopolymers and proteins in the model glycocalyx does not exceed 130 mg/mL, implying that solvent typically constitutes more than 90% of the film volume and that the film is spacious enough to accommodate more protein. We therefore conclude that the lack of Le^*x*^ glycan saturation in the model glycocalyx could arise from steric occlusion of glycans that are too close to the first CTB pentamer to allow their interaction with additional pentamers and/or from unfavorable entropic effects of sterically constraining the glycopolymers in the glycocalyx upon binding (i.e., reduced conformational entropy).

The balance of intrinsic affinity of the toxins for their target glycans and of steric and entropic effects imposed by the supramolecular glycocalyx organization is likely to be quite intricate. In this regard, it is remarkable that a simple Langmuir isotherm, which assumes all binding sites to be equal and independent from each other, could reproduce the concentration-dependent binding of CTB and STxB to our glycocalyx models very well (Figure 6B). Quite possibly, more complex-binding isotherms would emerge for protein concentrations higher than those we could test here. Indeed, previous experimental and theoretical analyses of globular, multivalent proteins binding to brushes of flexible “sticky” polymers have revealed complex-binding kinetics with a sustained logarithmic dependence of protein binding on protein concentration.^91^ Such an effect could lead to a higher occupancy of glycan-binding sites than predicted by the Langmuir isotherm, and the above determined *K*_d_ values should be considered apparent values valid to a good approximation only for sufficiently low toxin concentrations.

Even though CTB is not able to complex all of the Le^*x*^ glycans in the model glycocalyx, it still showed a greater binding enhancement than for the STxB-Gb_3_ system, and it also had the greater level of superselectivity (Figure 7). Density-dependent enhancements in multivalent protein-glycan interactions have been reported for a variety of lectins and antibodies.^8,12,15,23^ The superselective recognition of CTB and STxB relying on the target glycan density in the glycocalyx was not reported previously, yet is clearly apparent through slopes α > 1 in Figure 7B,C. It is also in contrast to the widely observed phenomenon that increasing ganglioside GM1 concentration in a membrane leads to a reduction in binding affinity for CTB.^92–94^ This phenomenon has been attributed to clustering of GM1 in the membrane at higher concentrations.^94^ We would not expect a similar clustering phenomenon in our 3D glycocalyx model based on HA polymers. Conversely, in other cases, low binding at very low ligand densities has been attributed to ligand spacing being too great to allow multivalent interactions.^15,23^ Nevertheless, following previous theoretical and experimental work with other interaction systems (reviewed in ref 52), we propose that the main driver for superselective binding is the combinatorial entropy associated with linking multiple receptors (here, glycans on the mucin-like structures) to multiple ligands (here, glycan-binding sites on the lectin). In particular, our observation of enhanced superselectivity with decreasing lectin concentration (Figure 7B,C) is indeed predicted by theoretical models of multivalent binding that take into account the combinatorial entropy effects.^95^

Given the high valency of STxB, it is somewhat surprising that the level of superselectivity is reduced for STxB binding to Gb_3_ compared to CTB binding to Le^*x*^Le^*x*^.^52^ Quite likely, the heterogeneity in affinity across the three structurally distinct Gb_3_-binding sites in STxB contributes to this effect. It is though also possible that variations in the background glycopolymers (Figure 7A) play a role here, as even very weak background interactions (e.g., potentially of STxB with Lac) could make a sizable contribution to the overall avidity when combined with Gb_3_, even if Lac alone was insufficient to generate any detectable STxB binding (Figure S9). These subtle effects of heterogeneous presentations of glycans and their binding sites merit further exploration.

The clear superselective recognition that we have evidenced here raises the intriguing possibility that AB_5_ toxins exploit subtle differences in target glycan densities to discriminate between cell types and bind their target cells with high selectivity. In the case of CT, it has been shown that fucosylated glycoproteins, including mucins, enhance cell binding and intoxication.^39^ In contrast, binding to fucosylated glycolipids confers protection to the cells. We here demonstrates that low affinity ligands, such as Le^*x*^ for CTB, can mediate high avidity recognition in model glycocalyces. Superselective binding to structures bearing Le^*x*^ higher in the glycocalyx (e.g., mucins) thus could direct the toxin to cells where it is most likely to have the greatest biological effect. Such density-dependent binding phenomena may have broader implications in glycobiology. For example, different subpopulations of antibodies might evolve or be selected to recognize antigens displayed at varying densities, enabling the immune system to detect both sparsely and densely presented targets.^12^

The enhanced multivalent and superselective binding of CTB to Le^*x*^ is in stark contrast to the lack of observable binding to the lactosyl glycopolymers (Figure S11). Several groups have demonstrated that multivalent galactose- and Lac-based compounds can be used as inhibitors of CTB binding to GM1-coated surfaces,^96,97^ and Lac has been shown to bind to the E. coli heat-labile toxin GM1-binding site, which is almost identical to the GM1-binding site in CTB.^98^ The affinity of CTB for simple galactosides is about 15 mM,^34^ which is only about 2-fold lower than its affinity for Le^*x*^.^36,69^ Therefore, it might have been expected that CTB would also bind to the lactosyl glycocalyx models. It may be that the mismatch in multivalent architecture for the lactosyl glycocalyx and CTB has a greater impact on binding than for the Gb_3_ glycocalyx binding the ST*x*B, as the latter binds with a 10-fold higher monovalent affinity.

We have illustrated how mucin-like glycopolymers can be assembled into glycocalyx models with defined physical (e.g., thickness, mesh size, and charge) and tunable chemical (e.g., target glycan concentration) properties, and how such glycocalyx models can reveal the impact of the glycocalyx microenvironment on multivalent protein binding (e.g., avidity and selectivity). The modular assembly strategy facilitates the design of glycocalyx models of varying complexity, and our experimental and analytical framework can be adapted in future studies to ask more complex questions about multivalent glycan recognition. More complex structures could be achieved, for example, by attaching different glycans to the same backbone or by copresenting short and long glycopolymers for a stratified presentation of multiple glycan types, thus enabling exploration of heteromultivalent-binding processes. Glycocalyx models should also be versatile for the mechanistic analyses of binding affinity and selectivity of other (endogenous or exogenous) glycan-binding proteins or biomacromolecular complexes (e.g., viruses); of the effect of overall glycocalyx charge on binding in glycocalyxes; of dynamic clustering of glycopolymers or glycocalyx reorganization on protein binding; and of the effect of clustered target glycan presentation (e.g., presented at a high density per glycopolymer and low glycopolymer surface density vs low density per glycopolymer at high glycopolymer surface density). For example, density-variant glycopolymer microarrays have proven useful for evaluating the propensity of different lectins to cross-link mucin-like structures.^8^ Another broad area that is functionally important yet poorly understood and thus worthy exploration is the mechanism of transport of toxins and other glycan-binding proteins in glycocalyces.

## Conclusions

We have developed a method to make glycopolymers with mucin-like densities of glycans based on a HA backbone with pendant target glycans and a terminal anchor tag for the preparation of glycocalyx models. The modularity of glycopolymer synthesis and surface grafting enables designer glycocalyces with quantitively tunable physical and chemical properties. Such model glycocalyces enable detailed biophysical analysis of multivalent-binding processes and reveal new phenomena, as demonstrated here with regard to superselective recognition of target glycans by AB_5_ toxins. These and many other intriguing effects of multivalent contact between glycans and biomacromolecular complexes become amenable to mechanistic study with our glycocalyx models, shedding light on the various barrier functions of the glycocalyx in health and disease.

## Methods

A full description of experimental methods can be found in the Electronic Supporting Information (ESI).

### General Procedure for the Synthesis of HA-*g*-Glycan

HA (50 mg, 125.5 μmol –COOH, 1 equiv) was dissolved in MES buffer (15 mL, 100 mM, pH = 5.5) overnight at room temperature while placed on a rocker. DMTMM (1 to 6 equiv) was then added to the HA solution. After activation of the carboxylic acid groups for 10 min, propargylamine (1 to 6 equiv) was added. The mixture was placed on a rocker overnight at room temperature. The crude product was transferred to a dialysis bag (SnakeSkin dialysis tubing: 7000 MWCO) and dialyzed at room temperature against NaCl solution (1 M) for 24 h, followed by four dialyses against water, each for 24 h. The resulting solution was lyophilized and characterized by ^1^H NMR spectroscopy (500 MHz, D_2_O) and SEC-MALS.

### General Procedure for the Synthesis of HA-*g*-Glycan Glycopolymers

A solution containing HA-*g*-propargyl **6** (4 mM alkyne groups), azide-glycan **3**,^69^ **4**,^66^ or **5** ^72^ (4 mM), CuSO_4_ (1.2 mM), sodium ascorbate (30 mM), and tris­(3-hydroxypropyltriazolylmethyl)­amine (THPTA; 8 mM) was incubated at 37 °C overnight. The crude product was purified by dialysis (SnakeSkin dialysis tubing; MWCO 7000 Da), against 10 mM disodium ethylenediaminetetraacetic acid (EDTA) for 24 h, followed by two sequential dialyses against ultrapure water (each 24 h) at room temperature. Purified glycopolymers were lyophilized and characterized by ^1^H NMR spectroscopy (500 MHz, D_2_O) and SEC-MALS in 10 mM HEPES, 150 mM NaCl, pH 7.4.

### General Procedure for Biotinylation of HA-*g*-Glycan Glycopolymers

A solution of HA-*g*-glycan glycopolymer (final polymer concentration 25 μM, 5 mg/mL) in a solution of sodium acetate (50 mM), aniline (20 mM), and EZ-link alkoxyamine PEG_4_-biotin (Thermo Fisher; 75 μM) was incubated overnight at 37 °C at 300 rpm in a thermocycler. The following day, the product was purified using a desalting column (PD-10 G-25 with MWCO = 5000 Da; GE Healthcare), taking aliquots of 250 μL. The resulting fractions were analyzed to check for the presence of the polysaccharide by spotting 3 μL onto a TLC plate, which was dried and dipped in a solution of orcinol (20.2 mM) and sulfuric acid (0.9 M) in water, and heating with a heat gun. Fractions containing HA were then analyzed by QCM-D using a SAv presenting SLB, as described previously.^99^

### General Procedure for QCM-D Analyses

Experiments were performed with silica-coated QCM-D sensors (QSX303) in a Q-Sense E4 system (both Biolin Scientific, Västra Frölunda, Sweden) with flow modules operated at a rate of 20 μL/min and a working temperature of 23 °C for real-time in situ analyses of biomolecular-binding processes. The normalized frequency shift Δ*F* = Δ*f*_*i*_/*i* and the dissipation shift Δ*D* for overtone *i* = 5 are presented. The thickness of glycopolymer brushes was quantified from QCM-D data of brush formation through viscoelastic modeling,^100,101^ using the “small-load approximation” model in PyQTM.^102,103^

### General Procedure for SE Analyses

Experiments were performed with silicon wafer pieces as sensing surfaces on a spectroscopic rotating compensator ellipsometer (M2000V; J.A. Woollam; NE, USA) with a custom-built open cuvette at room temperature for real time in situ analysis of biomolecular-binding processes.^104^ Temporal changes in the thickness and refractive index of the biomolecular film were obtained through fitting of the measured ellipsometric angles Δ and Ψ (as a function of the wavelength λ) with an optical model composed of multiple optically isotropic layers representing the substrate, the adsorbed biomolecular films, and the surrounding buffer solution, using the CompleteEASE software (J. A. Woollam, Co., Inc.). Areal mass densities (AMDs) were determined from the film thickness and refractive index through a variant of de Fejter’s equation.^105^

## Supplementary Material



## Data Availability

The raw data associated with this paper are available from the University of Leeds data repository (10.5518/1694) or the authors upon request.

## Notes

Experimental procedures for the preparation of all glycopolymers, QCM-D data, SE data, ^1^H NMR, and SEC-MALS data for polymers (PDF)

## #

Université de Paris, Faculté des Sciences, Campus Saint-Germain-des-Prés, UMR CNRS 8601, LCBPT, 45 rue des Saints Pères, F-75006 Paris, France
