# Supplementary material for: Molecularly Defined Glycocalyx Models Reveal AB5 Toxins Recognize Their Target Glycans Superselectively
Source: JACS Au. 2025 May 20;5(6):2699–712. doi: 10.1021/jacsau.5c00305 (PMC12188407; doi:10.1021/jacsau.5c00305)
Supplement: Supplementary file 1 [file au5c00305_si_001.pdf]

ELECTRONIC SUPPORTING INFORMATION (ESI)

Molecularly defined glycocalyx models reveal AB_5 toxins recognize their target glycans superselectively

Laia Saltor Núñez,[a,b†] Vajinder Kumar,[a,b] James F. Ross,[a,b,c] Jonathan P. Dolan,[a,b] Sumitra Srimasorn,[b,d,e] Xiaoli Zhang,[b,d,e] Ralf P. Richter[b,d,e] and W. Bruce Turnbull[a,b]

[a)] School of Chemistry, University of Leeds, Leeds LS2 9JT, United Kingdom; [b)] Astbury Centre for Structural Molecular Biology, University of Leeds, Leeds LS2 9JT, United Kingdom; [c)] School of Molecular and Cellular Biology, University of Leeds, Leeds LS2 9JT; [d)] School of Biomedical Sciences and School of Physics and Astronomy, University of Leeds, Leeds LS2 9JT, United Kingdom; [e)] Bragg Centre for Materials Research, University of Leeds, Leeds LS2 9JT, United Kingdom

Table of contents

Supporting figures 2
Supporting tables 16
Supporting methods 17
  Preparation of glycopolymers and other reagents 17
    Materials and methods 17
    Synthetic procedures 17
    Enzymatic procedures 18
    Polymer conjugation procedures 19
    Peptide synthesis 21
    Preparation of small unilamellar lipid vesicles (SUVs) for SLB formation 22
  Biophysical characterisation of glycocalyx models 23
    Quartz crystal microbalance with dissipation monitoring (QCM-D) 23
    Spectroscopic ellipsometry (SE) 23
    Calculating other physiochemical properties of the glycocalyx models 24
Appendix 26
  NMR data 26
  SEC-MALS data 33
Supporting references 35

SUPPORTING FIGURES

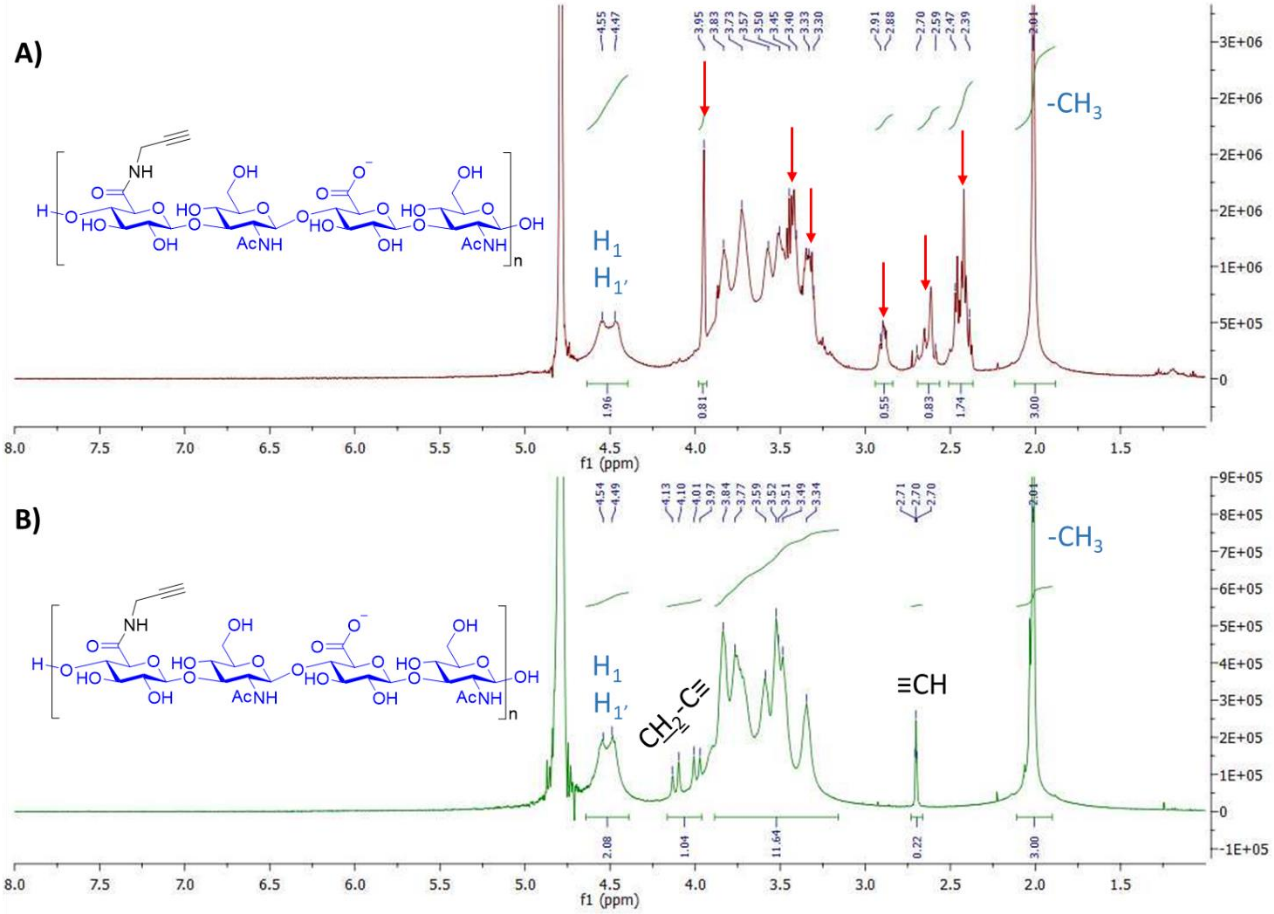

Figure S1: Comparative analysis of the purity of HA-*g*-propargyl when synthesized with EDC/NHS *vs.* DMTMM. A) Impure HA-*g*-propargyl synthesized with EDC and NHS as activators. Red arrows on top of the spectrum denote EDC-associated impurities. B) Pure HA-*g*-propargyl synthesized with DMTMM as activator. Data correspond to HA-*g*-propargyl **6** (Table S1; Test 1, *DS* = 22%), and the purity is representative for all HA-*g*-propargyl products made with DMTMM.

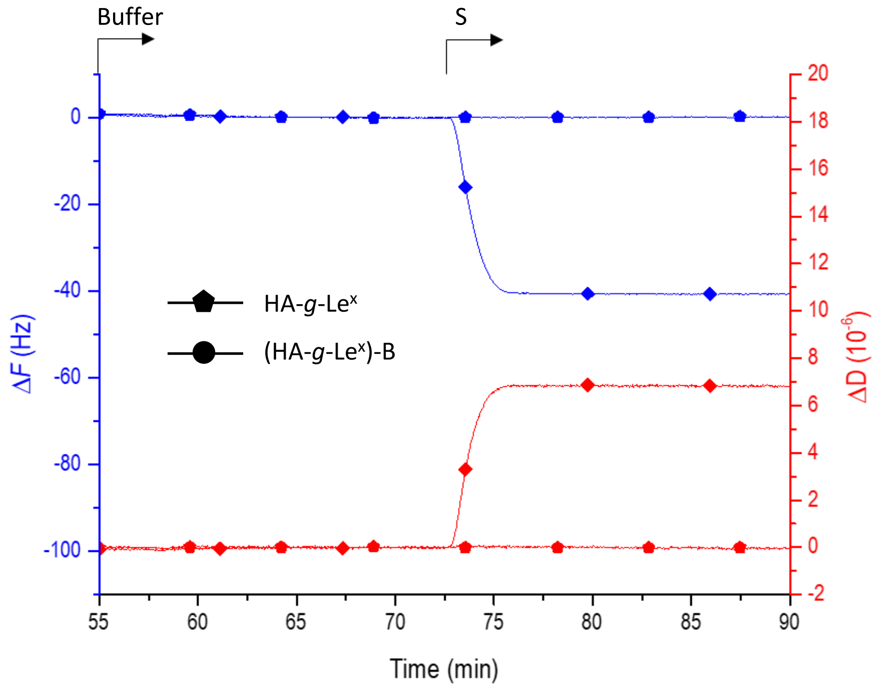

Figure S2: QCM-D data (frequency shift - Δ*F*, dissipation shift – Δ*D*; overtone *i* = 5) demonstrating specific and stable anchorage of (HA-*g*-Le^x)-B *via* the terminal biotin on a SAv-on-SLB surface. Conditions: SAv-on-SLB surfaces (formation not shown here; see Figure 4 for an example and conditions); glycopolymer sample (S) - 20 µg/mL **HA-*g*-Le^x** (lines with pentagon symbols) or **(HA-*g*-Le^x)-B** (lines with diamond symbols) in HBS; working buffer (Buffer) – HBS. Arrows atop the graph indicate the start of incubation with each sample as indicated.

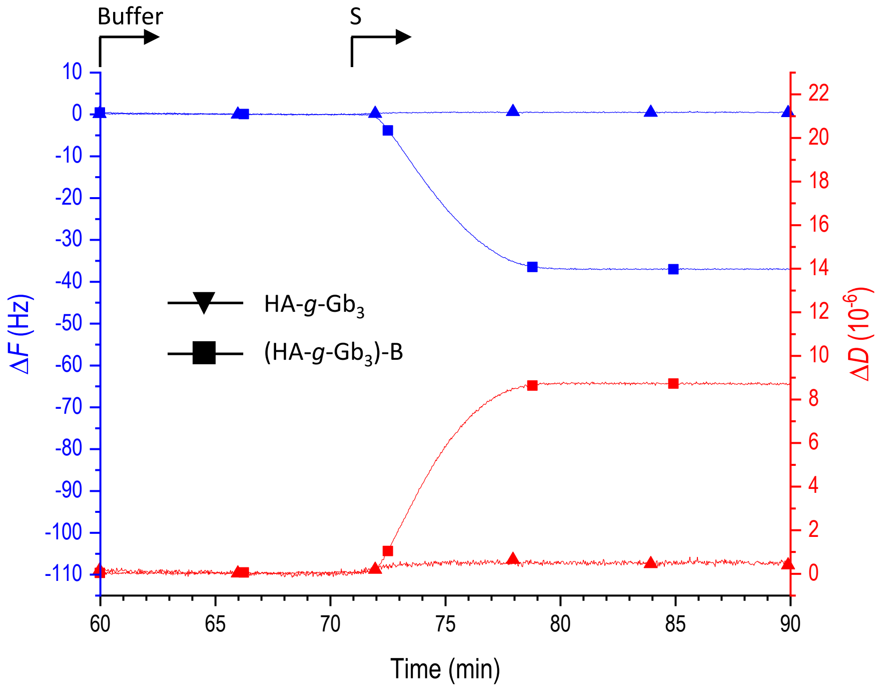

Figure S3: QCM-D data (frequency shift - Δ*F*, dissipation shift – Δ*D*; overtone *i* = 5) demonstrating specific and stable anchorage of (HA-*g*-Gb$_3$)-B *via* the terminal biotin on a SAv-on-SLB surface. Conditions: SAv-on-SLB surfaces (formation not shown here; see Figure 4 for an example and conditions); glycopolymer sample (S) - 20 µg/mL **HA-*g*-Gb$_3$** (lines with triangle symbols) or **(HA-*g*-Gb$_3$)-B** (lines with square symbols; data taken from Figure 5 and shown here to facilitate comparison) in HBS; working buffer (Buffer) – HBS. Arrows atop the graph indicate the start of incubation with each sample as indicated.

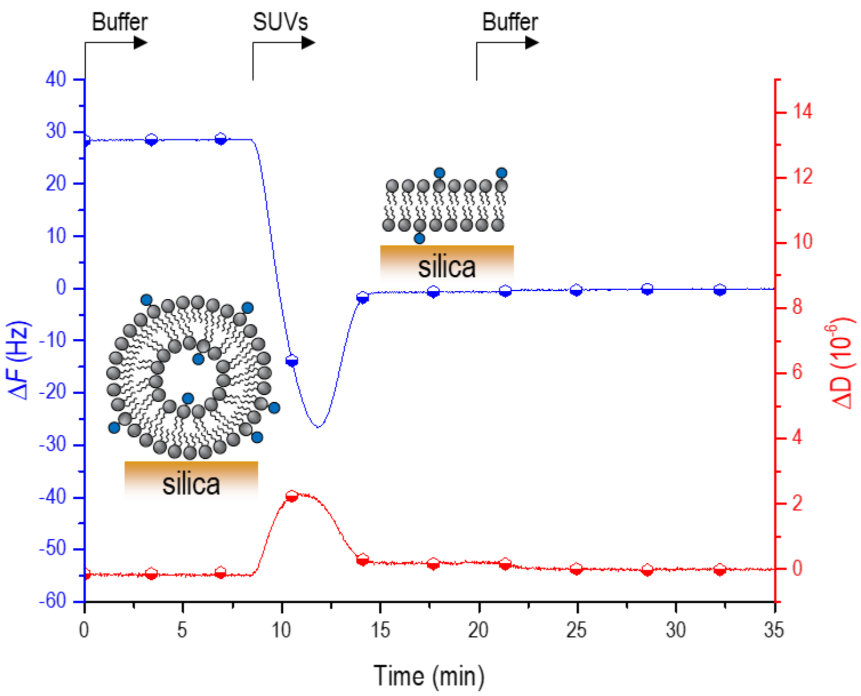

Figure S4: Formation of a Ni^{2+}-NTA presenting SLB recorded by QCM-D (frequency shift - Δ*F*, dissipation shift – Δ*D*; overtone *i* = 5). Conditions: SUVs – 50 µg/mL DOPC:(Ni^{2+}-NTA)$_3$-DODA 95:5 (mol:mol) in HBS with 5 mM $NiCl_2$; working buffer (Buffer) – HBS. Arrows atop the graph indicate the start of incubation with each sample as indicated.

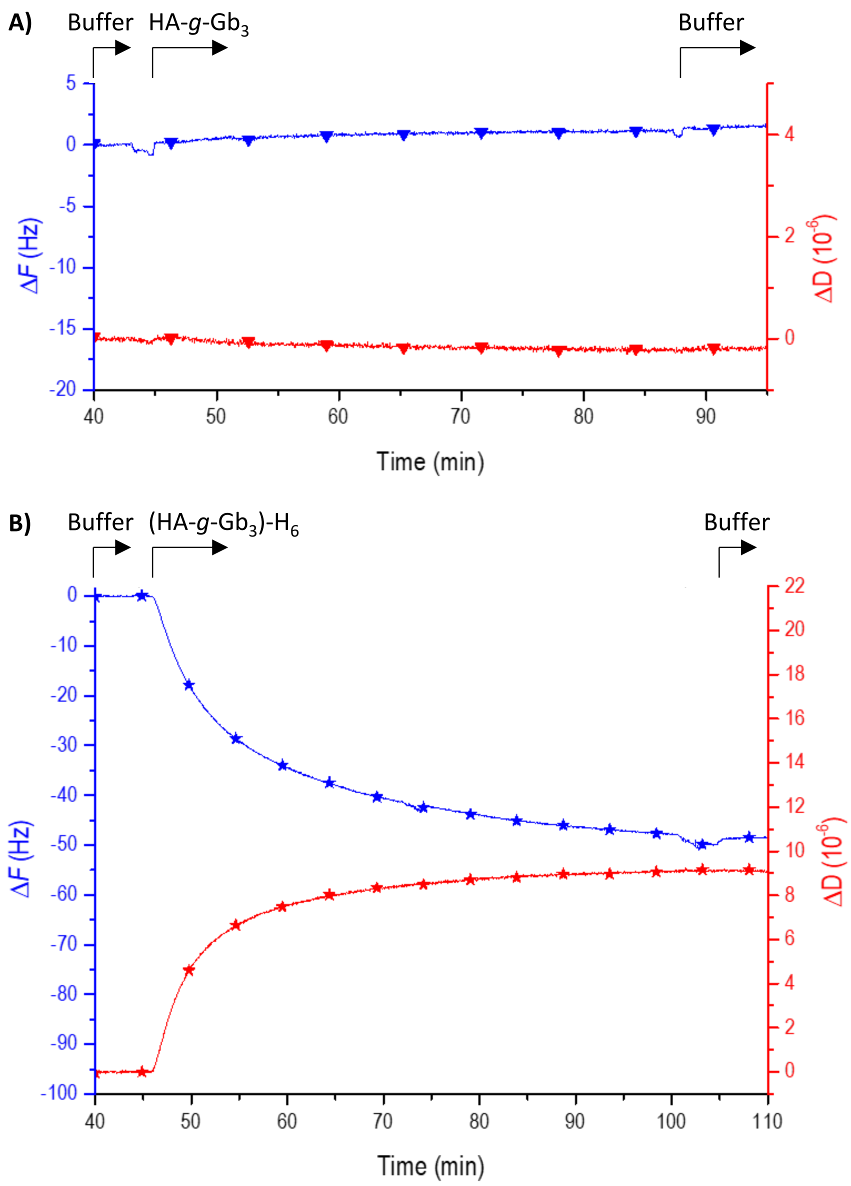

Figure S5: QCM-D data (frequency shift - Δ*F*, dissipation shift – Δ*D*; overtone *i* = 5) demonstrating specific and stable anchorage of (HA-*g*-Gb$_3$)-H$_6$ *via* the terminal H$_6$ on a Ni^{2+}-NTA presenting SLB. Conditions: Ni^{2+}-NTA presenting SLB (formation not shown here; see Figure S4 for an example and conditions); glycopolymer sample - 20 µg/mL **HA-*g*-Gb$_3$** (A) or **(HA-*g*-Gb$_3$)-H$_6$** (B) in HBS; working buffer (Buffer) – HBS. Arrows atop the graph indicate the start of incubation with each sample as indicated.

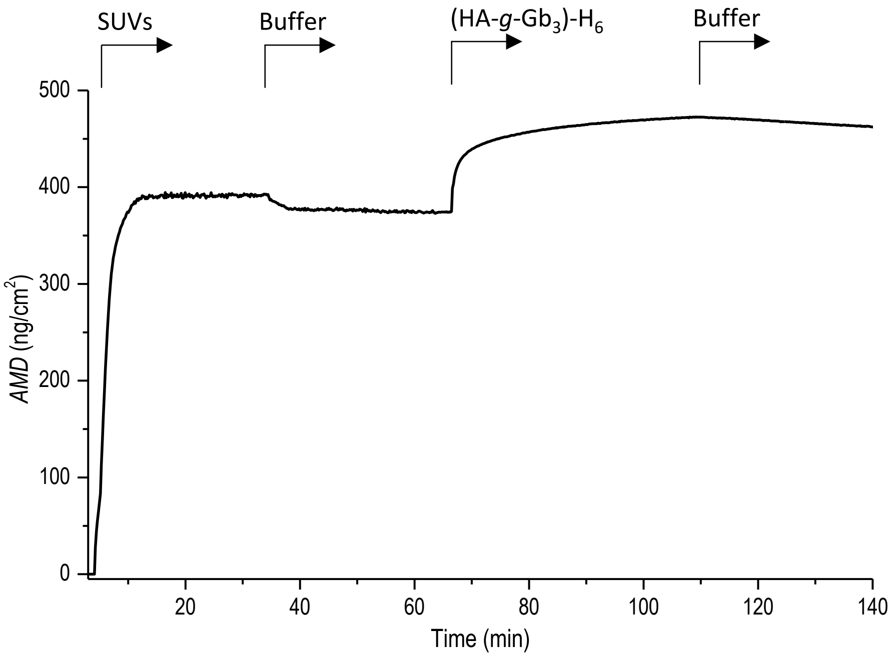

Figure S6: Formation of (HA-*g*-Gb_3)-H_6 brushes quantified by SE. Graph of areal mass density (*AMD*) against time for the formation of a Ni^{2+}-NTA presenting SLB and a brush of **(HA-*g*-Gb_3)-H_6**. Conditions: SUVs – 50 µg/mL DOPC:(Ni^{2+}-NTA)$_3$-DODA 95:5 (mol:mol) in HBS with 5 mM $NiCl_2$; glycopolymer sample – 20 µg/mL **(HA-*g*-Gb_3)-H_6** in HBS; working buffer (Buffer) – HBS. Arrows atop the graph indicate the start of incubation with each sample as indicated.

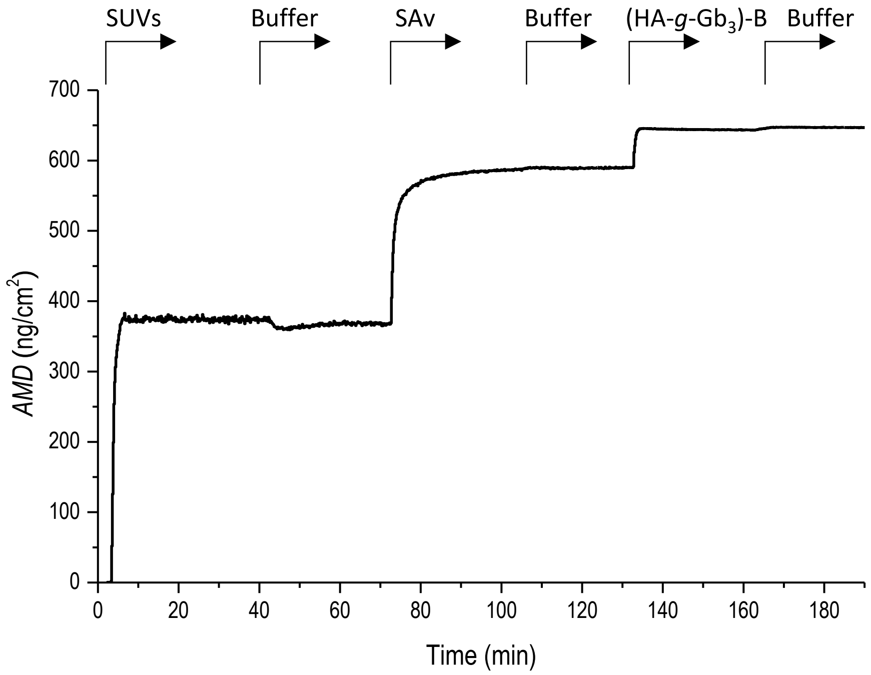

Figure S7: Formation of (HA-*g*-Gb$_3$)-B brushes quantified by SE. Graph of areal mass density (*AMD*) against time for the sequential formation of a biotin-presenting SLB, a monolayer of streptavidin (SAv), and a brush of **(HA-*g*-Gb$_3$)-B**. Conditions: SUVs – 50 µg/mL DOPC:DOPE-CAP-B 95:5 (mol:mol) in HBS; SAv – 20 µg/mL; glycopolymer sample – 20 µg/mL **(HA-*g*-Gb$_3$)-B** in HBS; working buffer (Buffer) – HBS. Arrows atop the graph indicate the start of incubation with each sample as indicated.

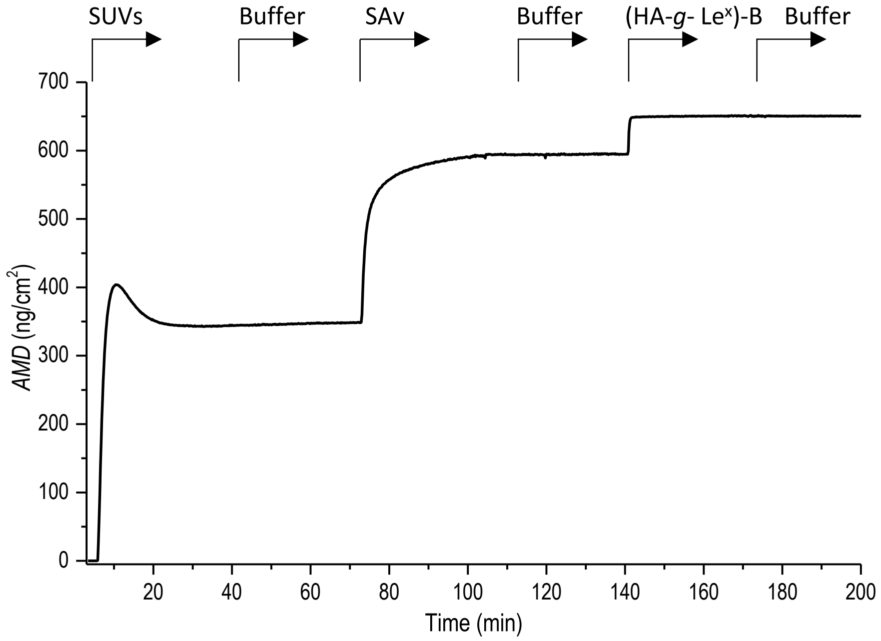

Figure S8: Formation of (HA-*g*-Le^x)-B brushes quantified by SE. Graph of areal mass density (*AMD*) against time for the sequential formation of a biotin-presenting SLB, a monolayer of streptavidin (SAv), and a brush of **(HA-*g*-Le^x)-B**. Conditions: SUVs – 50 µg/mL DOPC:DOPE-CAP-B 95:5 (mol:mol) in HBS; SAv – 20 µg/mL; glycopolymer sample – 20 µg/mL **(HA-*g*- Le^x)-B** in HBS; working buffer (Buffer) – HBS. Arrows atop the graph indicate the start of incubation with each sample as indicated.

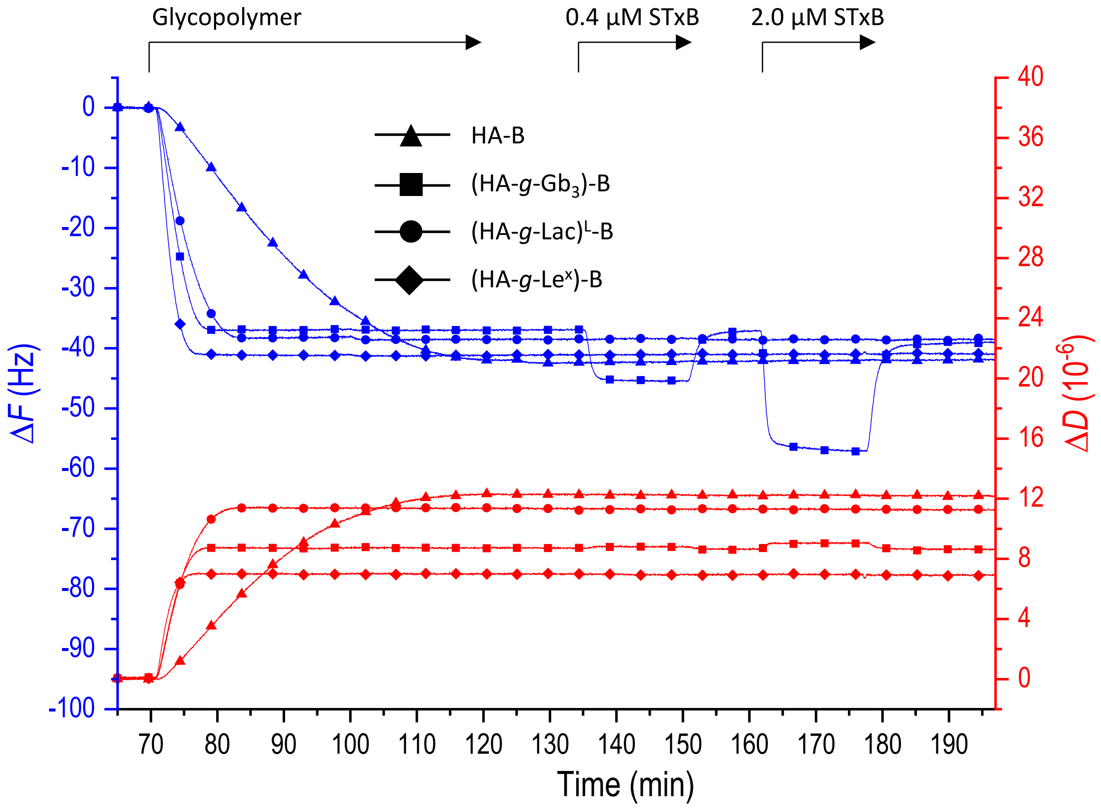

Figure S9: Additional control experiments demonstrating specificity of STxB binding to model glycocalyces with Gb3. QCM-D data (frequency shift - ΔF, dissipation shift – ΔD; overtone i = 5) show no measurable interaction of STxB with model glycocalyces presenting plain HA (lines with triangle symbols), pendant Lac glycans (lines with circle symbols) or pendant Le^x glycans (lines with diamond symbols). For comparison, data for productive interactions with a model glycocalyx presenting Gb_3 are reproduced here from Figure 5. Conditions: SAv-on-SLB surface (formation not shown here; see Figure 4B for an example and conditions); glycopolymer sample - 20 µg/mL **HA-B**, **(HA-*g*-Lac)^L-B**, **(HA-*g*-Gb_3)-B** or **(HA-*g*-Le^x)-B** (with symbols as indicated) in HBS; STxB – concentrations as indicated, in HBS; working buffer – HBS. Arrows atop the graph indicate the start and duration of incubation with each sample as indicated; during remaining times plain working buffer was flowed over the sensor.

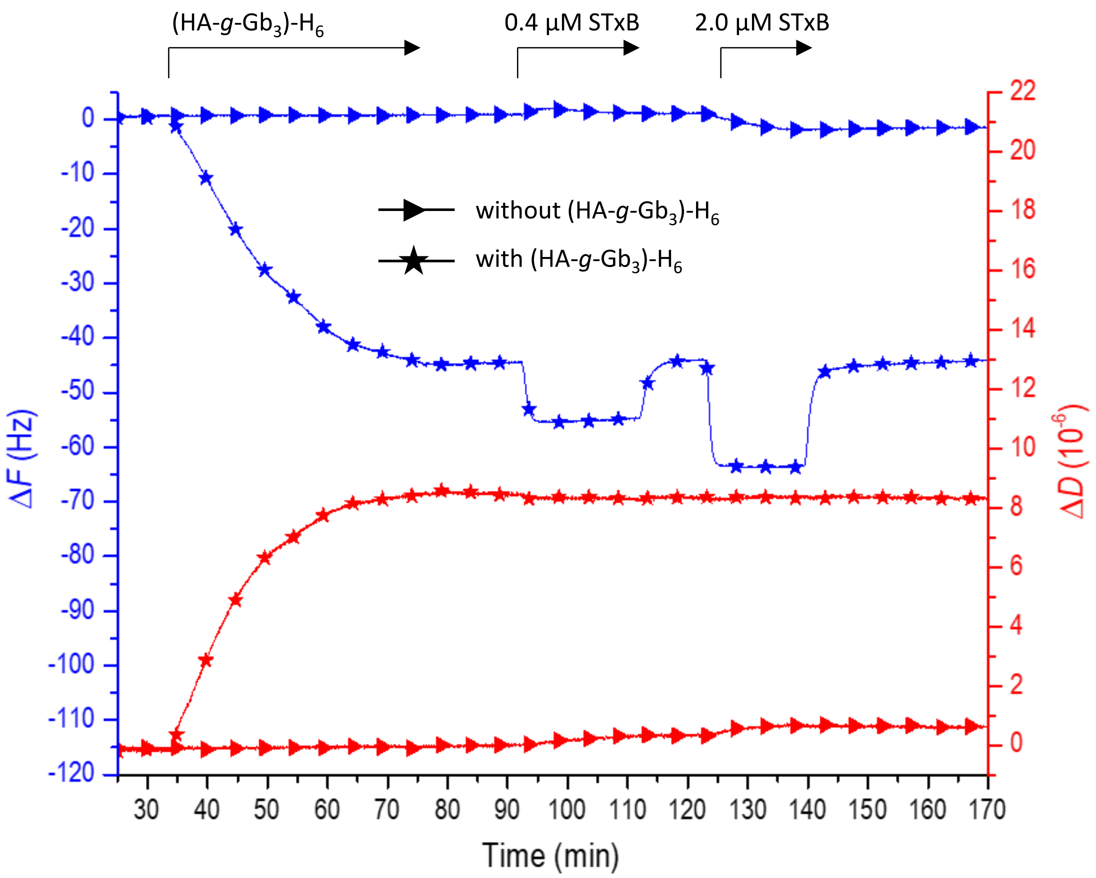

Figure S10: QCM-D data (frequency shift - ΔF, dissipation shift – ΔD; overtone i = 5) demonstrating specific and reversible binding of STxB to a model glycocalyx made from (HA-*g*-Gb$_3$)-H$_6$ on a Ni^{2+}-NTA presenting SLB. Conditions: Ni^{2+}-NTA presenting SLB (formation not shown here; see Figure S1 for an example and conditions); glycopolymer sample – **(HA-*g*-Gb$_3$) -H$_6$** – 40 µg/mL (lines with star symbols) or none (lines with triangle symbols); STxB' – concentrations as indicated, in HBS; working buffer – HBS. Arrows atop the graph indicate the start and duration of incubation with each sample as indicated; during remaining times plain working buffer was flowed over the sensor.

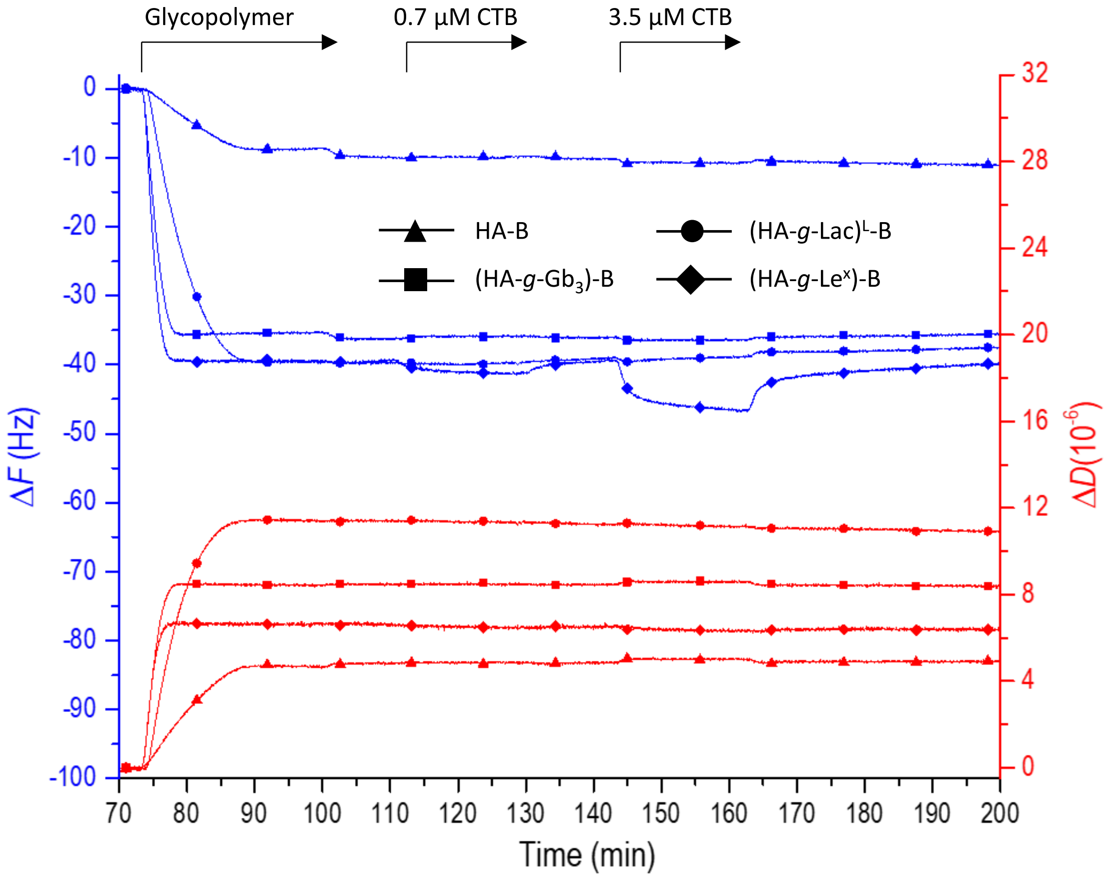

Figure S11: QCM-D data (frequency shift - Δ*F*, dissipation shift – Δ*D*; overtone *i* = 5) demonstrating specific binding of CTB to model glycocalyces with Le^x. The data show measurable interactions of CTB with model glycocalyces presenting Le^x (lines with diamond symbols), but not with model glycocalyces presenting plain HA (lines with triangle symbols), pendant Lac glycans (lines with circle symbols) or pendant Gb_3 glycans (lines with square symbols). Conditions: SAv-on-SLB surface (formation not shown here; see Figure 4B for an example and conditions); glycopolymer sample - 20 µg/mL **HA-B**, **(HA-*g*-Lac)^L-B**, **(HA-*g*-Gb_3)-B**, or **(HA-*g*-Le^x)-B** (with symbols as indicated) in HBS; CTB – concentrations as indicated, in HBS; working buffer – HBS. Arrows atop the graph indicate the start and duration of incubation with each sample as indicated; during remaining times plain working buffer was flowed over the sensor.

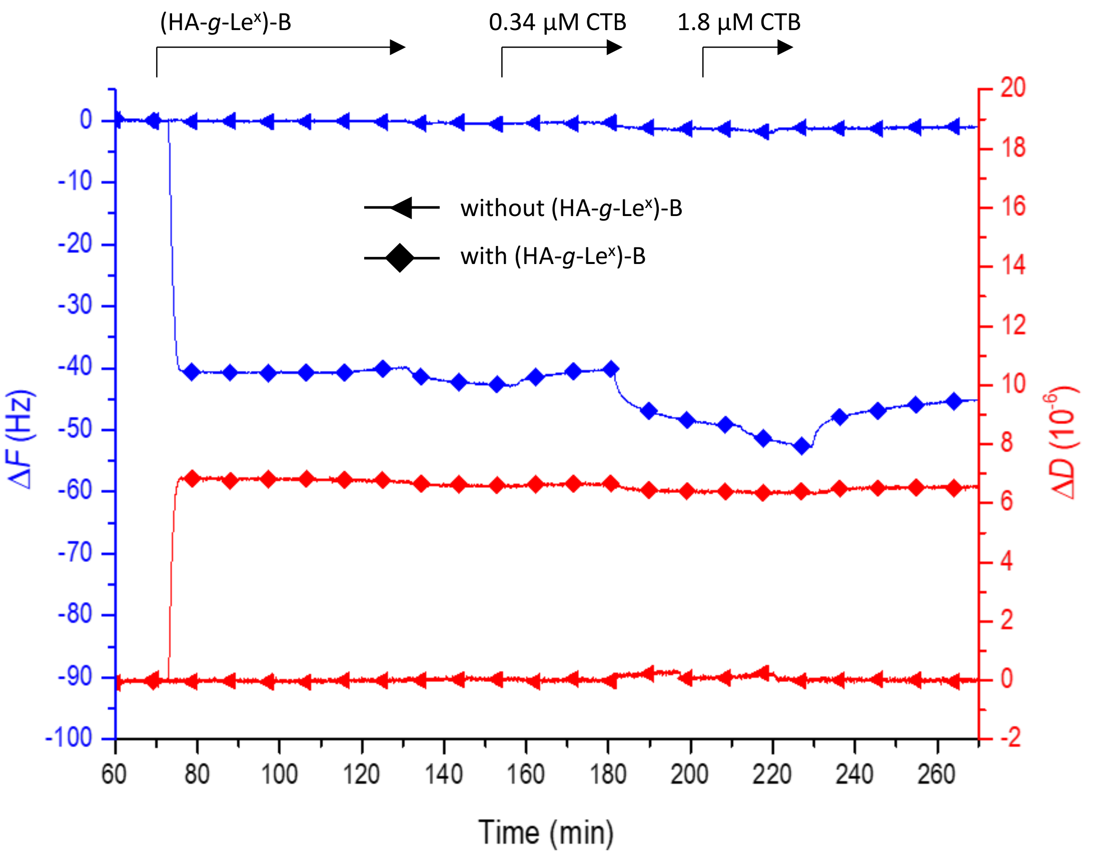

Figure S12: QCM-D data (frequency shift - Δ*F*, dissipation shift – Δ*D*; overtone *i* = 5) demonstrating specific and reversible binding of CTB to a model glycocalyx made from (HA-*g*-Le^x)-B on a SAv-on-SLB surface. Conditions: SAv-on-SLB surface (formation not shown here; see Figure 4B for an example and conditions); glycopolymer sample **- (HA-*g*-Le^x)-B** – 20 µg/mL (lines with diamond symbols) or none (lines with triangle symbols); CTB – concentrations as indicated, in HBS; working buffer – HBS. Arrows atop the graph indicate the start and duration of incubation with each sample as indicated; during remaining times plain working buffer was flowed over the sensor.

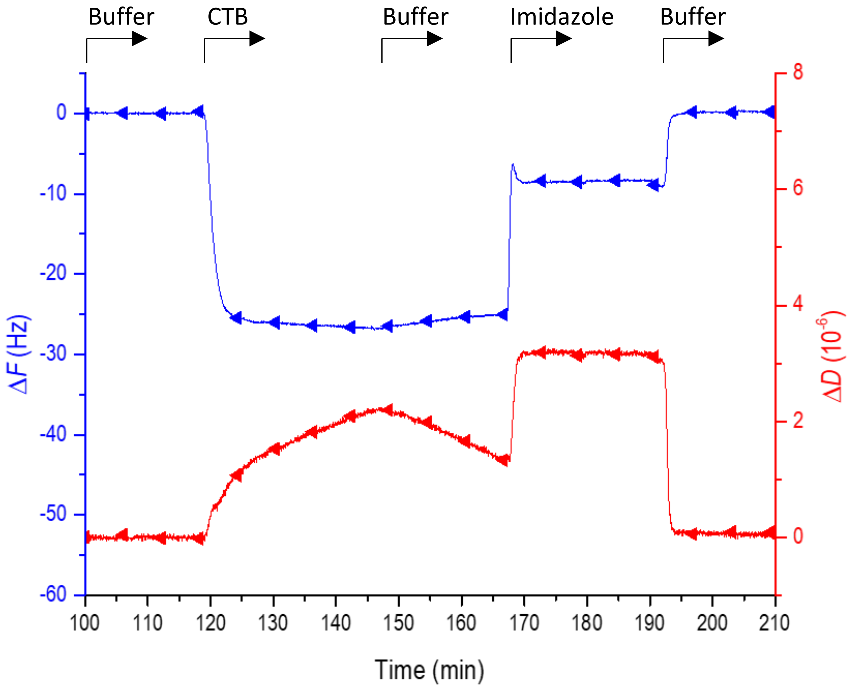

Figure S13: QCM-D data (frequency shift - Δ*F*, dissipation shift – Δ*D*; overtone *i* = 5) demonstrating binding of native CTB with a Ni^{2+}-NTA presenting SLB. Pronounced binding is seen that is partially stable in working buffer but can be rapidly eluted with imidazole. Conditions: Ni^{2+}-NTA presenting SLB (formation not shown here; see Figure S1 for an example and conditions); CTB – 0.34 µM in HBS; working buffer (Buffer) – HBS; imidazole – 500 mM in HBS. Arrows atop the graph indicate the start of incubation with each sample as indicated.

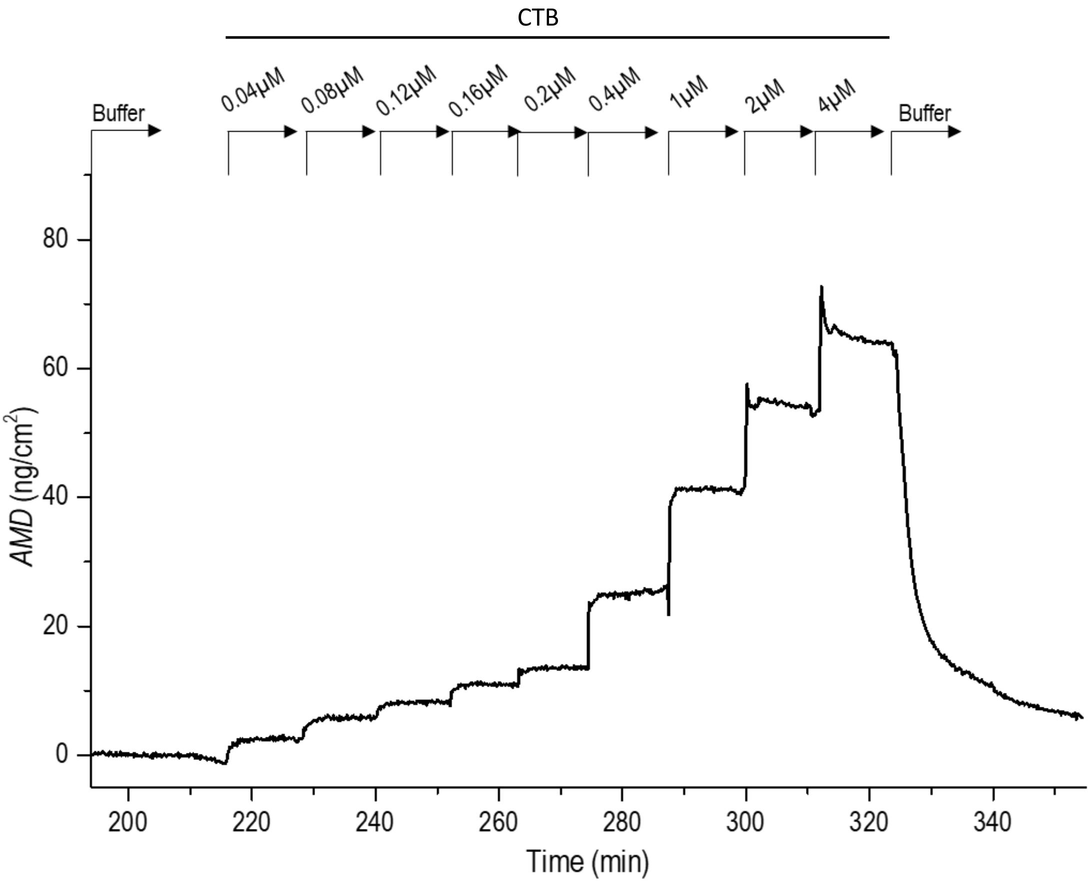

Figure S14: Titration of CTB in a model glycocalyx presenting Le^x quantified by SE. Graph of areal mass density (*AMD*) against time during CTB titration. Conditions: SAv-on-SLB surfaces with **(HA-g-Le^x)-B** grafted to saturation (data not shown here; see Figure S8 for details and conditions). CTB – incubated with stepwise increasing concentrations (from 0 to 20 μM, as indicated), in HBS; working buffer (Buffer) – HBS. Arrows atop the graph indicate the start of incubation with each sample as indicated.

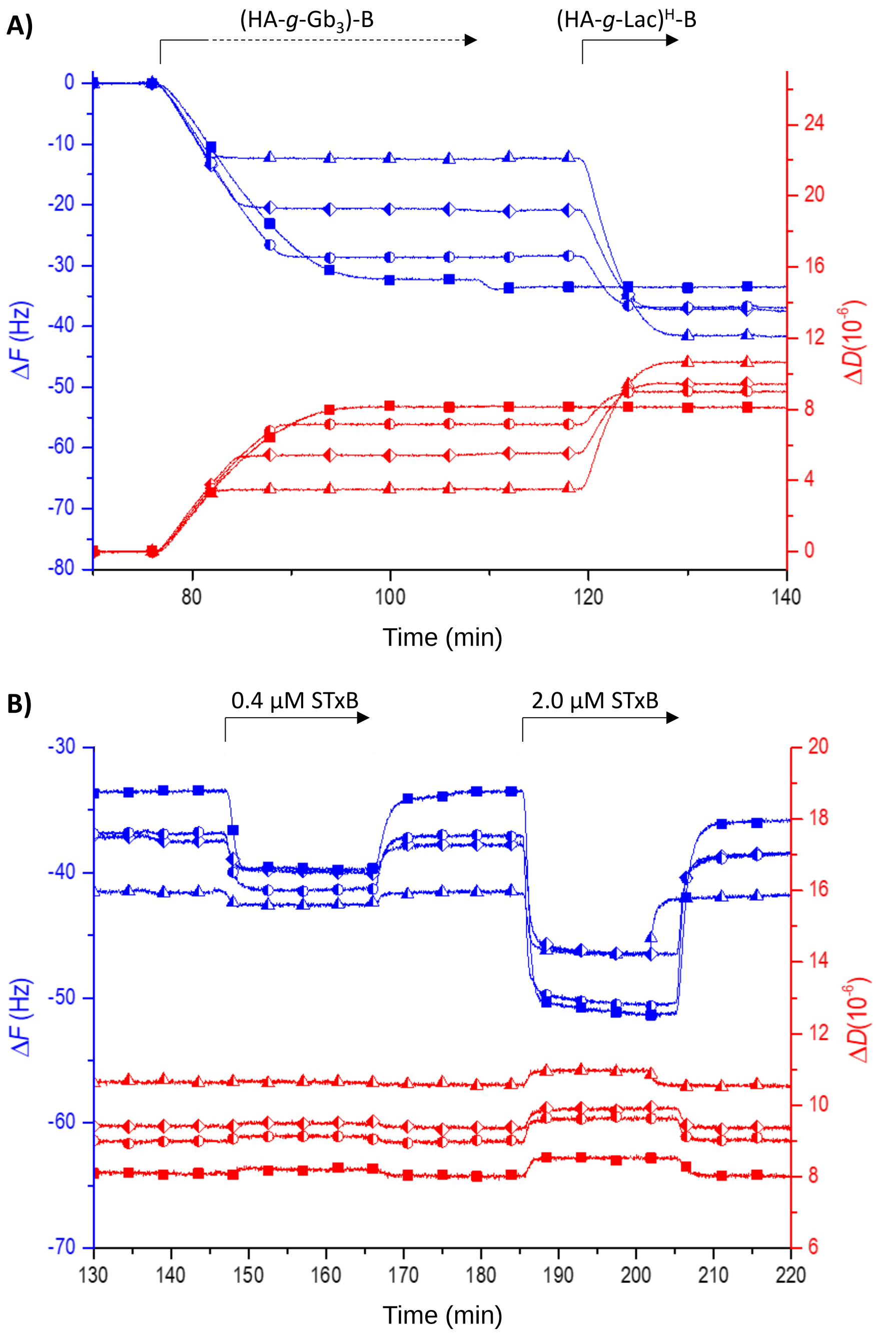

Figure S15: Formation of model glycocalyces with tuneable Gb3 concentration, and dependence of STxB binding on Gb3 concentration in model glycocalyces. Binding processes were monitored by QCM-D (frequency shift - ΔF, dissipation shift – ΔD; overtone i = 5). A) Formation of glycocalyx models made by sequential incubation of two distinct mucin-like glycopolymers. **(HA-*g*-Gb3)-B** was incubated first, with the incubation times tuned to obtain 37 ± 2 % (line with triangle symbols), 62 ± 3 % (line with diamond symbols), 85 ± 5 % (line with circle symbols) and 100 ± 7 % (line with square symbols) of the maximal **(HA-*g*-Gb3)-B** surface coverage; Gb3 percentages were quantified from the ratio of frequency shifts post **(HA-*g*-Gb3)-B** incubation (errors reflect reproducibility of the 100% surface coverage, and measurement accuracy for all surface coverages). Remaining biotin binding sites on the surface were then backfilled with **(HA-*g*-Lac)^{H}-B** to achieve similar total glycopolymer surface density and film thickness irrespective of Gb3 content. B) Dependence of STxB binding on Gb3 concentration in model glycocalyces. STxB binding was monitored for the model glycocalyces as shown in (A), with matching symbol code. Negative frequency shifts for STxB at equilibrium ($-\Delta F_{eq,STxB}$) were taken as a measure for the magnitude of STxB binding in Figure 7B. Conditions: SAv-on-SLB surface (formation not shown here; see Figure 4B for an example and conditions); **(HA-*g*-Gb3)-B** – 20 µg/mL (incubated for 5, 8, 12 and 32 min, respectively); **(HA-*g*-Lac)^{H}-B** – 20 µg/mL (incubated to saturation); STxB – concentrations as indicated, in HBS; working buffer – HBS. Arrows atop the graph indicate the start and duration of incubation with each sample as indicated; during remaining times plain, working buffer was flowed over the sensor.

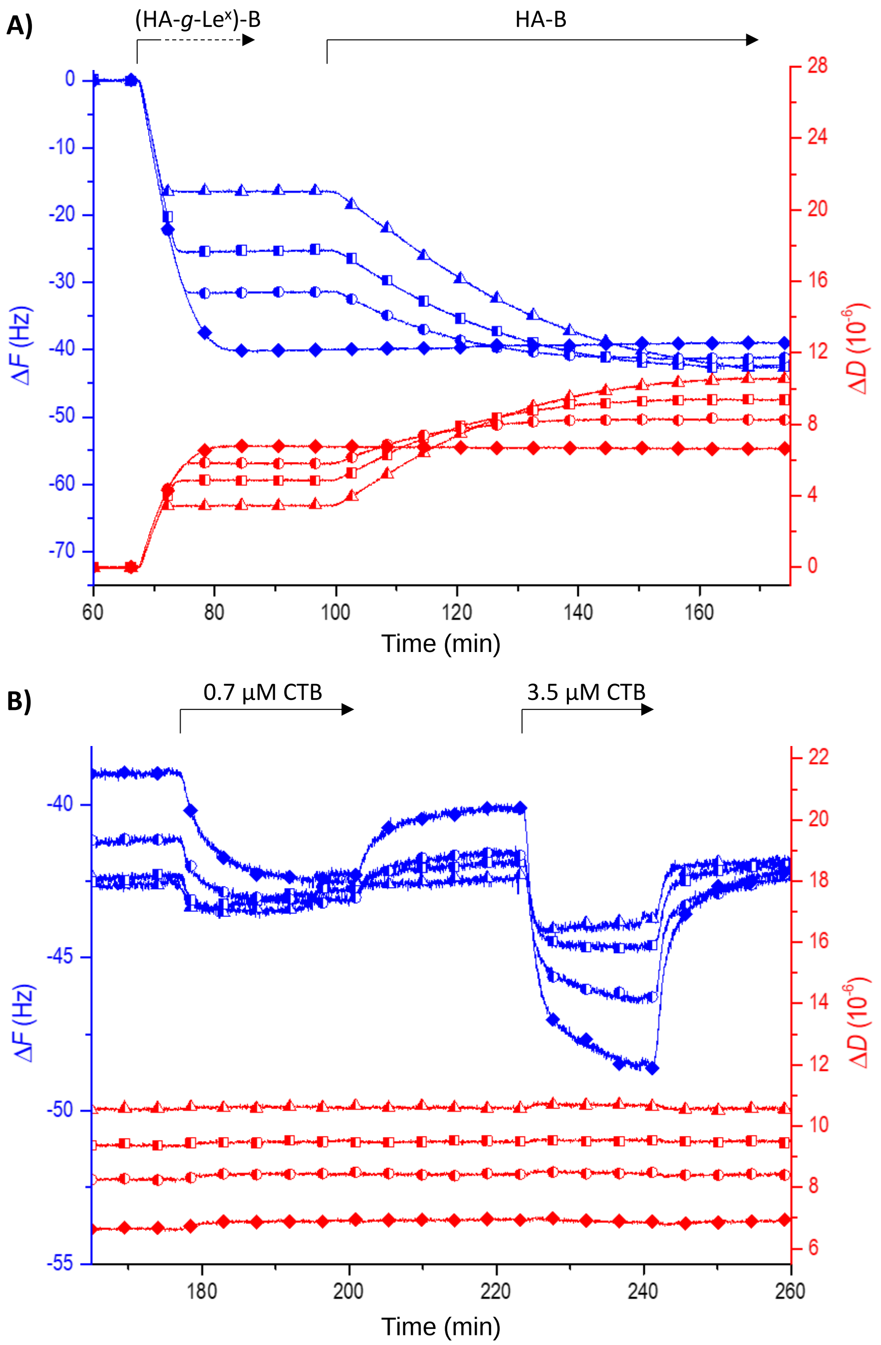

Figure S16: Formation of model glycocalyces with tuneable Le^x concentration, and dependence of CTB binding on Le^x concentration in model glycocalyces. Binding processes were monitored by QCM-D data (frequency shift - ΔF, dissipation shift – ΔD; overtone i = 5. A), and data are presented analogous to Figure S15. Formation of glycocalyx models made by sequential incubation of two distinct mucin-like glycopolymers. **(HA-*g*-Le^x)-B** was incubated first, with the incubation time tuned to obtain 41 ± 2 % (line with triangle symbols), 63 ± 3 % (line with square symbols), 79 ± 4 % (line with circle symbols) and 100 ± 7 % (line with diamond symbols) of the maximal **(HA-*g*-Le^x)-B** surface coverage. Remaining biotin binding sites were then backfilled with **HA-B** to achieve a similar total glycopolymer surface density and film thickness irrespective of Le^x content. B) Dependence of CTB binding on Le^x concentration in model glycocalyces. CTB binding was monitored for the model glycocalyces as shown in (A), with matching symbol code. Negative frequency shifts at the end of CTB incubation (-$\Delta F_{\mathrm{limit,CTB}}$) were taken as a measure for the magnitude of CTB binding in Figure 7C. Conditions: SAv-on-SLB surface (formation not shown here; see Figure 4B for an example and conditions); **(HA-*g*-Le^x)-B** – 20 µg/mL (incubated for 4.5, 6.5, 8 and 20 min, respectively); **HA-B** – 20 µg/mL (incubated to saturation); CTB – concentrations as indicated, in HBS; working buffer – HBS.

SUPPORTING TABLES

Table S1: Amounts of reactants used for preparation of HA-*g*-propargyl **6** (*via* reaction with DMTMM) and resulting *DS* (Figures S1B, S22 and S23).

**Experiment**	**DMTMM**			**Propargylamine**			***DS***
	mg	µmol	eq.	µL	µmol	eq.	
**Test 1**	35	125.5	1	8	125.5	1	22%
**Test 2**	105	376.5	3	24	376.5	3	33%
**Test 3**	210	753	6	48	753	6	50%

Table S2: Mass average molar mass (M_w), number average molar mass (M_n), polydispersity index ($Đ$) and z-average radius of gyration (R_g) obtained from SEC-MALS analyses of glycopolymers (Figures S28-30).

**Sample**	M_w (kDa)	M_n (kDa)	$Đ = M_w / M_n$	R_g (nm)
**HA-*g*-Propargyl 6** (22%)	177.8 ± 2.5	109.1 ± 3.1	1.63 ± 0.05	50.0 ± 1.2
**HA-*g*-Propargyl 6** (33%)	141.5 ± 2.0	81.9 ± 3.1	1.73 ± 0.06	50.3 ± 1.4
**HA-*g*-Propargyl 6** (50%)	235.0 ± 11.7	171.3 ± 9.4	1.37 ± 0.10	58.1 ± 3.7
**HA-*g*-Lac^L**	78.7 ± 2.3	39.8 ± 2.1	1.98 ± 0.11	27.1 ± 2.9
**HA-*g*-Lac^H**	115.8 ± 4.4	83.5 ± 3.6	1.39 ± 0.09	33.6 ± 4.7
**HA-*g*-Le^x**	30.0 ± 1.4	21.9 ± 1.3	1.36 ± 0.11	18.7 ± 9.5
**HA-*g*-Gb$_3$**	93.1 ± 2.7	60.0 ± 1.7	1.55 ± 0.07	31.9 ± 3.4

SUPPORTING METHODS

Preparation of glycopolymers and other reagents

Materials and methods

Materials. All dried solvents used were obtained from the School of Chemistry dry solvent system or otherwise dried according to standard methods. All solvents used for flash chromatography were HPLC grade except acetone which was GPR grade. The glassware for reactions performed under dry conditions were kept in the oven overnight and under N_2 atmosphere during the reaction. Materials and reagents used came from Sigma-Aldrich/Merk, Fisher Scientifics, Alfa Aesar, Fisher, Fluorochem or Carbosynth.

NMR methods. ^{1}H NMR, ^{13}C NMR, ^{1}H-^{1}H COSY and ^{1}H-^{13}C HSQC spectra were recorded on Bruker AV-NEO 500-CP or Bruker AV3HD-400 spectrometers. Chemical shifts are given in parts per million (ppm) referenced either to an internal standard (TMS) or to the residual solvent signal.[1] The following abbreviations are used to describe ^{1}H NMR signals: br = broad signal, s = singlet, d = doublet, t = triplet, q = quartet, quint = quintet, sx =sextuplet, sp = septuplet, dd= double doublet, dt = double triplet and ddd = double double doublet.

Mass spectrometry methods. Electrospray (ES+) ionisation (ESI) mass spectra were obtained on a Bruker HCT-Ultra mass spectrometer. High-resolution ES+ was performed on a Bruker Daltonics MicroTOF mass spectrometer. LC-MS analysis was performed on a Brucker AmaZon X series LC-MS spectrometer.

Chromatography methods. Aluminium foil coated with silica gel (60 F, 1.2 mm) for thin layer chromatography was from Merck. To visualize the spots, several methods and reagents were used depending on the nature of the analytes: UV light (254 nm), 1% solution of potassium permanganate in water, 5% solution of sulfuric acid in methanol, 5% H_2SO_4/methanol solution or orcinol solution (20.2 mM)/ H_2SO_4 (0.9 M) in water.
Flash chromatography was used to purify products. Chromatogel silica gel (35-70 µm) was used as stationary phase. Mixtures of organic solvents used as eluents are listed in each experimental method. Alternatively, a Biotage flash purification system was used to purify products using 10 g or 100 g SNAP stationary phase cartridges and a mixture of organic solvents as eluents described in each experimental method. Size exclusion chromatography was performed using a Biogel P2 column attached to a GE Pharmacia ÄKTA Prime FPLC system.

SEC-MALS analyses of glycopolymers. Glycopolymers were loaded onto a Superdex-75 10/300 (GE) column running at a flow rate of 0.75 ml/min in phosphate-buffered saline (PBS; pH 7.4). Samples eluting from the column passed through a Wyatt Helios 18-angle laser photometer. A coupled Wyatt Optilab TrEX detector further provided access to in-line analysis of the refractive index. The radius moments, molecular mass moments and concentrations of the resulting peaks were analysed using Astra V6 software. Weight (M_w) and number (M_n) average molecular masses, the dispersity $Đ = M_w/ M_n$, and the z-average radius of gyration (R_g) are reported in Table S2.

Synthetic procedures

2-Methyl-(3,4,6-tri-O-acetyl-1,2-dideoxy-α-D-glucopyranoso)[1,2-d]-2-oxazoline [2,3]
TMSOTf (0.6 mL, 2.8 mmol, 1.1 eq.) was added to a solution of 1,3,4,6-tetra-O-acetyl-*N*-acetylglucosamine (1 g, 2.57 mmol, 1 eq.) dry DCM (25 mL) under N_2. The reaction was stirred for 1.5 h at room temperature. Then, TEA (1.5 mL) was added to the mixture and the solvents were removed under vacuum to obtain a yellow-brown oil. The resulting product was purified by silica gel column chromatography (elution with hexane/ethyl acetate (EtOAc) from 50:50 to 10:90). Finally, the title compound was obtained as a colourless oil (760 mg, 90%).
TLC (Hexane/ EtOAc 3:1): R_f = 0.22
^{1}H NMR (400 MHz, $CDCl_3$): δ 5.95 (d, *J* = 7.6 Hz, 1H, H_1); 5.26 (t, *J* = 2.4 Hz, 1H, H_3); 4.93 (ddd, *J* = 1.3 Hz, *J* = 2.0 Hz, *J* = 9.3 Hz, 1H, H_4); 4.17-4.10 (m, 3H, H_2, H_6, H_6'); 3.61 (dt, *J* = 4.3 Hz, *J* = 8.9 Hz, 1H, H_5); 2.12 (s, 3H, Me); 2.10 (s, 3H, Me); 2.9 (d, *J* = 1.8 Hz, 3H, Me); 2.08 (s, 3H, Me).
^{13}C NMR (100 MHz, $CDCl_3$): δ 170.6 (Me-($\underline{C}$=N)-O-), 169.6 (-($\underline{C}$=O)-Me), 169.2 (-($\underline{C}$=O)-Me), 166.7 (-($\underline{C}$=O)-Me), 99.4 (C_1), 70.4 (C_3), 68.4 (C_4), 67.6 (C_5), 65.0 (C_2), 63.4 (C_6), 21.0 (-$\underline{C}H_3$), 20.9 (-$\underline{C}H_3$), 20.8 (-$\underline{C}H_3$), 14.2 (-$\underline{C}H_3$).
ESI MS (positive mode): *m/z*: Found 352.1021 $[M+Na]^+$ calc for $C_{14}H_{19}NO_8Na$ 352.1003.

3-Azidopropyl 2-acetamido-2-deoxy-3,4,6-tri-*O*-acetyl-β-D-glucopyranoside[4]
A solution of 2-methyl-(3,4,6-tri-O-acetyl-1,2-dideoxy-α-D-glucopyranoso)[1,2-d]-2-oxazoline (954 mg, 2.9 mmol, 1 eq.) in dry DCE (40 mL) under N_2 was heated for 30 min to ensure that the solution was at 80

ºC. Then, CSA (336 mg, 1.45 mmol, 0.5 eq.) and 3-azido-1-propanol (0.4 mL, 4.3 mmol, 1.5 eq.) were added to the 3.1 solution and the reaction mixture was left stirring overnight at 80 ºC. The following day, the mixture was colled to room temperature and 20 mL of DCM was added. The solution was washed three times with 60 mL of a saturated solution of sodium bicarbonate ($NaHCO_3$). The organic layer was dried over $MgSO_4$ and dried under vacuum. The brown oil obtained was purified using a SNAP 25 g biotage column (elution with Hexane/EtOAc from 50:50 to 100% EtOAc) to achieve 3-azidopropyl 2-acetamido-3,4,6-tri-*O*-acetyl-β-D-glucopyranoside as a white solid (370 mg, 30%).
TLC (EtOAc 100%): R_f = 0.38
^1H NMR (Figure S18; 500 MHz, $CDCl_3$): δ 5.61 (d, *J* = 8.9 Hz, 1H, -NH-); 5.22 (dd, *J* = 10.6 Hz, *J* = 9.3 Hz, 1H, H_3); 4.93 (t, *J* = 9.6 Hz, 1H, H_4); 4.61 (d, *J* = 8.3 Hz, 1H, H_1); 4.24 (dd, *J* = 12.3 Hz, *J* = 4.8 Hz, 1H, H_6); 4.13 (dd, *J* = 12.3 Hz, *J* = 2.5 Hz, 1H, $H_{6'}$); 3.97-3.89 (m, 2H, H_2, H_a); 3.69 (ddd, *J* = 10.0 Hz, *J* = 4.8 Hz, *J* = 2.5 Hz, 1H, H_5); 3.58 (ddd, *J* = 9.7 Hz, *J* = 8.3 Hz, *J* = 4.7 Hz, 1H, $H_{a'}$); 3.40-3.32 (m, 2H, H_c); 2.08 (s, 3H, $C\underline{H_3}$); 2.02 (s, 3H, $C\underline{H_3}$); 2.01 (s, 3 H, $C\underline{H_3}$); 1.95 (s, 3H, $C\underline{H_3}$); 1.77-1.74 (m, 2H, H_b).
^{13}C NMR (Figure S18; 125 MHz, $CDCl_3$): 171.1 (-($\underline{C}$=O)-OCH_3), 170.8 (-($\underline{C}$=O)-OCH_3), 170.3 (-($\underline{C}$=O)-$NHCH_3$), 169.5 (-($\underline{C}$=O)-OCH_3), 101.2 (C_1), 72.5 (C_3), 72.0 (C_5), 68.6 (C_4), 66.2 (CH_a), 62.0 (C_6), 54.5 (C_2), 48.0 (CH_c), 28.9 (CH_b), 23.3 (-NH-$\underline{C}H_3$), 20.8 (-$\underline{C}H_3$), 20.7 (-$\underline{C}H_3$) and 20.6 (-$\underline{C}H_3$).
LC MS (positive mode): *m/z* Found 431.08 $[M+H]^+$ calc for $C_{17}H_{27}N_4O_9$ 431.18.

3-Azidopropyl 2-acetamido-2-deoxy-β-D-glucopyranoside (1)[4]
To solution of 3-azidopropyl 2-acetamido-2-deoxy-3,4,6-tri-*O*-acetyl-β-D-glucopyranoside (370 mg, 0.86 mmol, 1 eq.) in dry MeOH (5 mL) under N_2 was added 1.5 mL of 0.5 M sodium methoxide (MeONa) solution in $CHCl_3$. The mixture was stirred at room temperature for 3 hours. Then, it was neutralized with DOWEX-50 H^+ resin and the suspension as filtered. The resulting solution was dried under vacuum to achieve **1** as a white solid (177 mg, 68%)
TLC (DCE/MeOH/AcOH/H_2O 50:30:25:10): R_f = 0.59
^1H NMR (Figure S19; 500 MHz, CD_3OD): δ 4.38 (d, *J* = 8.4 Hz, 1H, H_1); 3.95 (dt, *J* = 10.0 Hz, *J* = 5.6 Hz, 1H, H_a); 3.88 (dd, *J* = 11.9 Hz, *J* = 2.2 Hz, 1H, H_6); 3.70-3.62 (m, 2H, H_2, $H_{6'}$); 3.56 (ddd, *J* =10.0 Hz, *J* = 7.3 Hz, *J* = 5.2 Hz, 1H, $H_{a'}$); 3.44 (dd, *J* = 10.4 Hz, *J* = 8.4 Hz, 1H, H_4); 3.38 (t, *J* = 6.7 Hz, 2H, H_c); 3.33-3.29 (under CD_2HOD peak, H_3); 3.26 (ddd, *J* = 9.7 Hz, *J* = 5.7, *J* = 2.2 Hz, 1H, H_5); 1.84-1.78 (m. 2H, H_b).
^{13}C NMR (Figure S19; 125 MHz, CD_3OD): δ 173.7 (-($\underline{C}$=O)-$NHCH_3$), 102.8 (C_1), 78.0 (C_5), 75.6 (C_4), 72.1 (C_3), 67.1 (CH_a), 62.8 (C_6), 57.4 (C_2), 48.9 (CH_c), 30.1 (CH_b) and 23.0 (-NH$\underline{C}H_3$).
ESI MS (positive mode): *m/z* Found 305.1451 $[M+H]^+$ calc for $C_{11}H_{21}N_4O_6$ 305.1455 and found 327.1275 $[M+Na]^+$ calc for $C_{11}H_{20}N_4O_6Na$ 327.1275.

Enzymatic procedures

3-Azidopropyl *N*-acetyllactosamine (2)
A solution of **2** (115 mg, 0.38 mmol, 16 mM); uridine diphosphate glucose (298 mg, 0.49 mmol, 21 mM); Bovine serum albumin (BSA, 1 mg/mL); β-1,4-galactosyltransferase 1 (β(1-4)GalT1) (1 mg/ mL);[5] *Bifidobacterium longum* UDP-Glucose-4-epimerase (1.75 mg/mL);[6] $MnCl_2$ (10 mM); and sodium chloride (100 mM) in Tris buffer (100 mM, pH 8.0, total volume 23.7 mL) was incubated overnight at 37 °C. The reaction mixture was subjected to mass spectrometry and TLC analysis to check if the reaction was successful. This crude mixture was used for the next enzymatic reaction without purification.
LC MS (positive mode): *m/z* Found 467.22 $[M+H]^+$ calc for $C_{17}H_{31}N_4O_{11}$ 467.20

3-Azidopropyl Le^x (3) [7]
A solution of **3** (0.38 mmol, 4 mM); L-fucose (125 mg, 0.76 mmol, 8 mM); adenosine triphosphate (770 mg, 1.52 mmol, 16 mM); guanosine triphosphate (397 mg, 0.76 mmol, 8 mM); *Bacteroides fragilis* GDP-fucose pyrophosphorylase (FKP) (4.4 µM);[5] *Helicobacter pyrol* α-1,3-fucosalytransferase (0.96 mg/mL);[5] $MgCl_2$ (10 mM) and sodium chloride (100 mM) in Tris buffer (100 mM, pH 8.0, total volume 95 mL) was incubated for two days at 37 °C. Then, MeOH (100 mL) was added to precipitate the proteins and the mixture was centrifuged. The resulting supernatant solution was mixed with silica and dried under vacuum to achieve a dry white powder, which was loaded onto a SNAP 10 g Biotage silica gel chromatography column and eluted with a gradient of EtOAc/MeOH (from 95:5 to 100% MeOH). The fractions which contained oligosaccharide (visualised on a TLC plate by dipping in a solution of 10% (*v/v*) sulphuric acid in MeOH and heating) were combined and the solvent was removed under vacuum. The remaining syrup was dissolved in 2 mL of water and purified by SEC (Biogel P2 resin) using ammonium formate (20 mM) in deionized water as eluent to obtain azidopropyl Le^x (124 mg, 53%)
TLC (DCE/MeOH/AcOH/H_2O 50:30:25:10): R_f = 0.30
^1H NMR (Figure S20; 500 MHz, D_2O): δ 5.11 (d, *J* = 4.0 Hz, 1H, $H_{1''}$); 4.84 (q, *J* = 6.5 Hz, 1H, $H_{5''}$); 4.54 (d, *J* = 8.1 Hz, 1H, H_1); 4.45 (d, *J* = 7.8 Hz, 1H, $H_{1'}$); 4.00 (dd, *J* = 12.4, *J* = 2.3 Hz, 1H, H_6); 3.98-3.92 (m, 1H, H_a); 3.92-

3.83 (m, 6H, H_2, H_3, $H_{3'}$, H_4, $H_{4'}$, H_{6a}); 3.79 (d, *J* = 2.7 Hz. 1H, $H_{4''}$); 3.76-3.64 (m, 5H, $H_{2''}$, $H_{3'}$, $H_{6'}$, $H_{a'}$); 3.61-3.57 (m, 2H, H_5, $H_{5'}$); 3.49 (dd, *J* = 9.9, *J* = 7.8 Hz, 1H, $H_{2'}$); 3.42-3.32 (m, 2H, H_c); 2.04 (s, 3H, C$\underline{H_3}$-CO-NH-); 1.84 (p, *J* = 6.5 Hz, 2H, H_b); 1.17 (d, *J* = 6.6 Hz, 3H, $H_{6''}$).
^{13}C NMR (Figure S20; 126 MHz, D_2O): δ 174.2 (-$\underline{C}$O-), 101.8 ($C_{1'}$), 100.9 (C_1), 98.6 ($C_{1''}$), 75.3 (C_5/$C_{5'}$), 74.9 (C_3/C_4/C_5/$C_{5'}$), 73.3 (C_3/C_4), 72.4 (C_3'), 71.9 (C_4''), 71.0 (C_2'), 69.2 (C_3''), 68.3 ($C_{4'}$), 67.7 ($C_{2''}$), 67.2 (CH_a), 66.7 ($C_{5''}$), 61.5 ($C_{6'}$), 59.7 (C_6), 55.8 (C_2), 47.7 (CH_c), 28.1 (CH_b), 22.2 (CO-$\underline{C}H_3$) and 15.3($C_{6''}$).
m/z Found 613.2568 $[M+H]^+$ calc for $C_{23}H_{41}N_4O_{15}$ 613.2562, and found 635.2574 $[M+Na]^+$ calc for $C_{23}H_{40}N_4O_{15}Na$ 635.2382.

Gb$_3$-N$_3$ (5)[8]
This compound was prepared and analysed (NMR data in Figure S21) as described in Reference 8.

Polymer conjugation procedures

HA-*g*-Propargyl: coupling with EDC/NHS
Hyaluronic acid (28 mg, 84 µmol -COOH, 1 eq.) was weighed in a 10 mL glass vial and dissolved in MES buffer (7 mL, 100 mM, pH = 6.0) by shaking gently overnight at room temperature. EDC (352 mg, 1680 µmol, 20 eq.) and NHS (172 mg, 1596 µmol, 19 eq.) were added in this order to the HA solution and the carboxylic acids were activated for 20 min. This step released gas bubbles. Then, propargylamine (21 µL, 336 µmol, 4 eq.) was added to the solution. The mixture was placed on a rocker overnight at room temperature. The crude product was transferred to a dialysis bag (SnakeSkin dialysis tubing: 7000 MWCO) and purified by dialysis at room temperature, successively against NaCl solution (1 M) for 24 h followed by four dialyses against water, each for 24 h. The resulting suspension was freeze-dried and analysed by ^{1}H NMR (see Figure S1A).

HA-*g*-Propargyl (6): coupling with DMTMM
Hyaluronic acid (50 mg, 125.5 µmol -COOH, 1 eq.) was dissolved in MES buffer (15 mL, 100 mM, pH = 5.5) overnight at room temperature while placed on a rocker. The following day DMTMM (210 mg, 753 µmol, 6 eq.) was added to HA solution and the carboxylic acid groups were activated for 10 min followed by the addition of propargylamine (48 µL, 753 µmol, 6 eq.). The mixture was placed on a rocker overnight at room temperature. The crude product was transferred to a dialysis bag (SnakeSkin dialysis tubing: 7000 MWCO) and purified by dialysis at room temperature, successively against NaCl solution (1 M) for 24 h followed by four dialyses against water, each for 24 h. The resulting solution was freeze-dried, and analysed by ^{1}H NMR and SEC-MALS. Three separate samples (Test 1–3) were prepared; see Figures S1B, S22-24 and below for ^{1}H NMR data, and Table S2 and Figure S29 for SEC-MALS data.
^{1}H NMR (500 MHz, D_2O): 4.54, 4.49 (2 × brs, H_1 and $H_{1'}$); 4.12 (d, *J* = 17.5 Hz, CHC≡C); 3.99 (d, *J* = 17.5 Hz, CH'C≡C); 3.91-3.30 (m, H_2, H_3, H_4, H_5, H_6, $H_{6'}$), 2.71 (s, C≡CH); 2.01 (s, CO-C$\underline{H_3}$).
For Test 1: ^{1}H NMR ratio for integrals of CO-C$\underline{H_3}$ *vs.* C≡CH: 3:0.22 (Figure S1B and Figure S22).
For Test 2: ^{1}H NMR ratio for integrals of CO-C$\underline{H_3}$ *vs* C≡CH: 3:0.33. (Figure S23).
For Test 3: ^{1}H NMR ratio for integrals of CO-C$\underline{H_3}$ *vs* C≡CH: 3:0.50 (Figure S24).

HA-*g*-Lac^L
HA-*g*-Propargyl 6 (Test 1; 22% derivatization with alkyne, 12.6 mg, 7.5 µmol alkyne. 1 eq.) was dissolved overnight in 1.5 mL of water in a 2 mL Falcon tube. The next day, in a 500 µL Eppendorf tube containing 207 µL ultrapure water, the following reagents were added in this order: **4** (18.4 µL from a stock 400 mM, 28.9 µmol, 1 eq.), $CuSO_4$ (4.4 µL from a stock 500 mM, 8.7 µmol, 0.3 eq.), sodium ascorbate (110.4 µL from a stock 500 mM, 216.9 µmol, 7.5 eq.) and THPTA (29.4 µL from a stock 500 mM, 57.8 µmol, 2 eq.). The activated azide was transferred to solution of HA-*g*-Propargyl resulting on a mixture of 7.23 mL containing these final concentrations: **6** (4 mM), **4** (4 mM), $CuSO_4$ (1.2 mM), sodium ascorbate (30 mM) and THPTA (8 mM). The reaction mixture was incubated at 37 ºC overnight. The crude product was transferred to a dialysis bag (SnakeSkin dialysis tubing: 7000 MWCO), and purified by dialysis at room temperature, successively against ethylenediaminetetraacetic acid solution (10 mM EDTA) for 24 h and two dialyses against water, each for 24 h. The resulting solution was freeze-dried and analysed by ^{1}H NMR and SEC-MALS.
^{1}H NMR (Figure S25; 500 MHz, D_2O): δ 8.21 (s, 0.11H, triazole-CH; 5.80 (d, *J* = 9.2 Hz, 0.13H, H_1''); 4.54 (m, 2H H_1 and $H_{1'}$); 4.08-3.44 (m); 3.42-3.27 (m); 2.71 (s, 0.04H, residual C≡CH), 2.01 (s, 3H, CO-C$\underline{H_3}$).
SEC-MALS (HEPES buffer 10 mM, NaCl 150 mM, pH 7.4): see Table S2 and Figure S30.

HA-g-Lac^{H}

HA-*g*-Propargyl **6** (Test 3; 50% derivatization with alkyne, 22.5 mg, 28.9 µmol alkyne. 1 eq.) was dissolved overnight in 1.5 mL of water in a 15 mL Falcon tube. The next day, in a 2 mL Eppendorf tube containing 790 µL ultrapure water, the following reagents were added in this order: **4** (18.4 µL from a stock 400 mM, 28.9 µmol, 1 eq.), $CuSO_4$ (4.42 µL from a stock 500 mM, 8.7 µmol, 0.3 eq.), sodium ascorbate (110.4 µL from a stock 500 mM, 216.9 µmol, 7.5 eq.) and THPTA (29.4 µL from a stock 500 mM, 57.8 µmol, 2 eq.). Then, the activated azide was transferred to solution of HA-*g*-Propargyl resulting on a mixture of 7.23 mL containing these final concentrations: **6** (4 mM), **4** (4 mM), $CuSO_4$ (1.2 mM), sodium ascorbate (30 mM) and THPTA (8 mM). The reaction mixture was incubated at 37 ºC overnight. The crude product was transferred to a dialysis bag (SnakeSkin dialysis tubing: 7000 MWCO), and purified by dialysis at room temperature, successively against ethylenediaminetetraacetic acid solution (10 mM EDTA) for 24 h and two dialyses against water, each for 24 h. The resulting solution was freeze-dried and analysed by ^{1}H NMR and SEC-MALS.
^{1}H NMR (Figure S26; 500 MHz, D_2O): δ 8.21 (s, 0.11H, triazole-CH); 5.80 (d, *J* = 9.2 Hz, 0.13H, $H_{1''}$); 4.54 (m, 2H H_1 and $H_{1'}$); 4.08-3.44 (m); 3.42-3.27 (m); 2.71 (s, 0.02H, residual C≡CH); 2.01 (s, 3H, CO-C$\underline{H_3}$).
SEC-MALS (HEPES buffer 10 mM, NaCl 150 mM, pH 7.4): see Table S2 and Figure S30.

HA-*g*-Gb$_3$

HA-*g*-Propargyl **6** (Test 3; 50% derivatization with alkyne, 22.7 mg, 29.0 µmol alkyne) was dissolved overnight in 5.8 mL of water in a 15 mL Falcon tube. The next day, in a 2 mL Eppendorf tube containing 790.1 µL ultrapure water, the following reagents were added in this order: **5** (72.5 µL from a stock 400 mM, 28.9 µmol, 1 eq.), $CuSO_4$ (17.4 µL from a stock 500 mM, 8.7 µmol, 0.3 eq.), sodium ascorbate (434 µL from a stock 500 mM, 216.9 µmol, 7.5 eq.) and THPTA (116 µL from a stock 500 mM, 57.8 µmol, 2 eq.). The activated azide was transferred to solution of HA-*g*-Propargyl resulting on a mixture of 7.23 mL containing these final concentrations: **6** (4 mM), **5** (4 mM), $CuSO_4$ (1.2 mM), sodium ascorbate (30 mM) and THPTA (8 mM). The reaction mixture was incubated at 37 ºC overnight. The crude product was transferred to a dialysis bag (SnakeSkin dialysis tubing: 7000 MWCO), and purified by dialysis at room temperature, successively against ethylenediaminetetraacetic acid solution (10 mM EDTA) for 24 h and two dialyses against water, each for 24 h. The resulting solution was freeze-dried and analysed by ^{1}H NMR and SEC-MALS.
^{1}H NMR (Figure S27; 500 MHz, D_2O): δ 8.21 (s, 0.35H, triazole-CH); 5.82-5.76 (m, 0.30H, $H_{1''}$); 4.97 (d, *J* = 3.9 Hz, 0.37H, $H_{1''''}$); 4.57-4.49 (m, 2H H_1 and $H_{1'}$); 4.08-3.44 (m); 3.42-3.27 (m); 2.71 (s, 0.10H, residual C≡CH), 2.01 (s, 3H, CO-C$\underline{H_3}$).
SEC-MALS (HEPES buffer 10 mM, NaCl 150 mM, pH 7.4): see Table S2 and Figure S31.

HA-*g*-Le^{x}

HA-*g*-Propargyl **6** (Test 3; 50% derivatization with alkyne, 22.2 mg, 28.9 µmol alkyne. 1 eq.) was dissolved overnight in 5.8 mL of water in a 15 mL Falcon tube. The next day, in a 2 mL Eppendorf tube containing 790 µL ultrapure water, the following reagents were added in this order: **3** (72.5 µL from a stock 400 mM, 28.9 µmol, 1 eq.), $CuSO_4$ (17.4 µL from a stock 500 mM, 8.7 µmol, 0.3 eq.), sodium ascorbate (434 µL from a stock 500 mM, 216.9 µmol, 7.5 eq.) and THPTA (116 µL from a stock 500 mM, 57.8 µmol, 2 eq.). The activated azide was transferred to solution of HA-*g*-Propargyl resulting on a mixture of 7.23 mL containing these final concentrations: **6** (4 mM), **3** (4 mM), $CuSO_4$ (1.2 mM), sodium ascorbate (30 mM) and THPTA (8 mM). The reaction mixture was incubated at 37 ºC overnight. The crude product was transferred to a dialysis bag (SnakeSkin dialysis tubing: 7000 MWCO), and purified by dialysis at room temperature, successively against ethylenediaminetetraacetic acid solution (10 mM EDTA) for 24 h and two dialyses against water, each for 24 h. The resulting solution was freeze-dried and analysed by ^{1}H NMR and SEC-MALS.
^{1}H NMR (Figure S28; 500 MHz, D_2O): δ 7.92 (s, 0.3H, triazole-CH); 5.12 (d, J = 3.9 Hz, 0.3H, $H_{1''''}$); 4.85 (q, J = 6.5 Hz, 0.3H, $H_{5''''}$); 4.63 – 4.45 (m, 2.6H, H1, $H_{1'}$, $H_{1''}$ and $H_{1'''}$); 4.00 – 3.28 (m, 13H); 2.71 (s, 0.04H, residual C≡CH); 2.16 – 2.13 (m, 0.6H, H_c); 2.04 – 1.97 (m, 3.9H, CO-C$\underline{H_3}$ and CO-C$\underline{H_{3''}}$); 1.17 (d, J = 6.6Hz, 0.9H, $H_{6''''}$).
SEC-MALS (HEPES buffer 10 mM, NaCl 150 mM, pH 7.4): see Table S2 and Figure S31.

(HA-*g*-Lac)^{L}-B/ (HA-*g*-Lac)^{H}-B/ (HA-*g*-Gb$_3$)-B/ (HA-*g*-Le^{x})-B/ HA-B

A solution of **2**/HA (final polymer concentration 25 µM, 5 mg/mL) was transferred to a 200 µL Eppendorf tube. Sodium acetate (final concentration 50 mM), aniline (final concentration 20 mM) and EZ-link alkoxyamine PEG_4-biotin (ThermoFisher; final concentration 75 µM) were added following the order described. The reaction mixture was incubated overnight at 37 ºC at 300 rpm in a thermocycler. The following day, the product was purified using a desalting column (Pd-10 G-25M with MWCO = 5000 Da; GE Healthcare) taking aliquots of 250 µL. The resulting fractions were analysed to check for the presence

of the polysaccharide by spotting 3 µL onto a TLC plate, which was dried and dipped in a solution of orcinol (20.2 mM) and sulfuric acid (0.9 M) in water, and heating with a heat gun. Fractions containing HA were then analysed by QCM-D using a streptavidin presenting supported lipid bilayer (see Figure 4 for SLB formation) and a previously established method [9] to determine which fractions contained **(HA-*g*-Lac)^{L}-B/ (HA-*g*-Lac)^{H}-B/ (HA-*g*-Gb$_3$)-B/ (HA-*g*-Le^x)-B/ HA-B**.

(HA-*g*-Gb$_3$)-H$_6$
A solution of **HA-*g*-Gb$_3$** (final glycopolymer concentration 25 µM (or 5 mg/mL)) was transferred to a 200 µL Eppendorf tube. Sodium acetate (final concentration 50 mM), aniline (final concentration 20 mM) and peptide **7** (final concertation 75 µM) were added following the order described. The reaction mixture was incubated overnight at 37 ºC at 300 rpm in a thermocycler. The following day, the product was purified using a desalting column (Pd-10 G-25M with MWCO = 5000 Da; GE Healthcare) taking elution aliquots of 250 µL. The resulting fractions were analysed to check for the presence of the polysaccharide by spotting 3 µL onto a TLC plate, which was dried and dipped in a solution of orcinol (20.2 mM) and sulfuric acid (0.9 M) in water, and heating with a heat gun. Fractions containing HA were then analysed by QCM-D using a Ni^{2+}-NTA presenting supported lipid bilayer (see Figure S4 for SLB formation) and a previously established method[9] to determine which fractions contained **(HA-*g*-Gb$_3$)-H$_6$.**

Peptide synthesis

Preparation of the resin. Fmoc-protected Rink amide resin methylbenzhydryl amine (MHBA) was weighed in a polypropylene syringe fitted with a polyethylene filter disc, and washed with DCM (3 × syringe volume), DMF (3 × syringe volume), MeOH (3 × syringe volume) and DCM (3 × syringe volume). Fmoc removal was achieved by reaction with 20% piperidine in DMF (1 syringe volume × 3 min + 1 syringe volume × 15 min); followed by washing with DMF (2 × syringe volume) and DCM (3 × syringe volume).

Peptide coupling. Incorporation of protected amino acids was accomplished by using 6 eq. of both Fmoc-amino acid, Oxyma Pure and DIC dissolved in the minimum amount of DMF. Coupling reactions were rotated for 90 min at room temperature. Afterwards, the resin was washed with DCM (3 × syringe volume), DMF (3 × syringe volume) and MeOH (3 × syringe volume). If the coupling was not complete, as assessed by heating some resin beads in a saturated solution of ninhydrin in MeOH. If the beads were blue, meaning the presence of free amines, the coupling was repeated using another 3 eq. of the reagents. If the beads stayed yellow, the coupling was successful.
After the coupling of the first 6 amino acids, a mini cleavage was performed to check the purity of the peptide.
Incorporation of 2-[(Bis-Boc)amino]oxyacetic acid on the peptide (*N*-terminal position) was accomplished by using 6 eq. each of this molecule, Oxyma Pure and DIC. The coupling reaction was left to proceed for 90 min at room temperature. The coupling was checked by heating some resin beads in a saturated solution of ninhydrin in MeOH.

Mini cleavage. A small sample of beads from the isolated deprotected amino acid-resin were exposed to 1 mL of the cleavage cocktail (95:2.5:2.5 (*v*/*v*/*v*) TFA/H_2O/TIS) for 45 minutes. The filtrate was collected and the mixture was evaporated under a N_2 stream. The remaining solid was dissolved in MeOH, filtered and analysed by LCMS.

Cleavage and isolation. Deprotection and cleavage was carried out by reaction with a 95:2.5:2.5 (*v*/*v*/*v*) TFA/H_2O/TIS mixture (1 mL for 100 mg of resin) for 2.5 h at room temperature. The filtrate was then collected and the resin was washed with TFA (5 mL or until the filtrate was not orange) which was added to the filtrate; then TFA was evaporated under a N_2 stream. The crude product was precipitated with diethyl ether. The peptide dissolved in water was lyophilized, purified by HPLC and quantified by spectrophotometry at 280 nm (Figure S17) and characterized by mass spectroscopy.

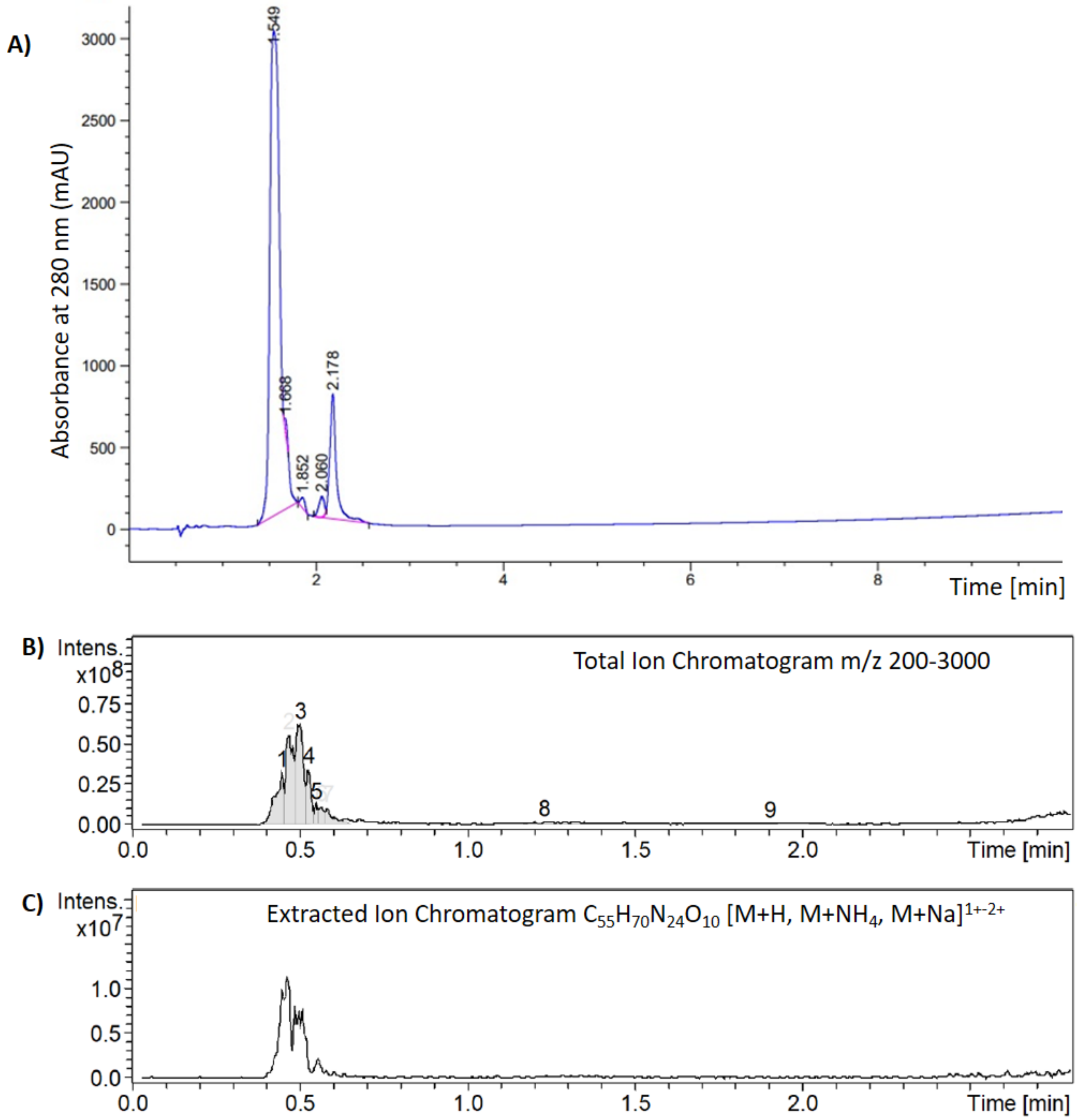

Figure S17: HPLC and LCMS chromatograms of the peptide H_2N-O-K-W-H_6-NH_2 7. A) Absorbance at 280 nm against time for reverse phase HPLC. The multiple peaks may correspond to the product in different states of protonation as the LCMS B) total ion chromatogram and C) extracted ion chromatogram for the peptide also shows several peaks, all of which correspond to the expected mass of the peptide.

Yield: 4.9 mg, 3%
ESI HRMS (positive mode): m/z: Found 1227.5819[M+H]$^+$ calc for $C_{55}H_{71}N_{24}O_{10}$ 1227.5779.
LCMS (positive mode): m/z: Found 307.58 [M+4H]$^{4+}$ calc for [$C_{55}H_{70}N_{24}O_{10}$ + 4H]$^{4+}$ 307.65; Found 409.81 [M+3H]$^{3+}$ calc for [$C_{55}H_{70}N_{24}O_{10}$ + 3H]$^{3+}$ 409.86; Found 614.64 [M+2H]$^{2+}$ calc for [$C_{55}H_{70}N_{24}O_{10}$ + 2H]$^{2+}$ 614.29.

Preparation of small unilamellar lipid vesicles (SUVs) for SLB formation

1,2-Dioleoyl-sn-glycero-3-phosphocholine (DOPC) was obtained from Avanti Polar Lipids (Alabaster, USA), as was a lipid analogue with two oleoyl tails and phosphoetanolamine headgroup with a conjugated linker and a terminal biotin (DOPE-CAP-B). A lipid analogue with two oleoyl tails and a chelator headgroup comprising three nitrilotriacetic acid moieties (DODA-(NTA)$_3$) was prepared as described previously[10] and kindly provided by Changjiang You and Jacob Piehler (Osnabrück University, Osnabrück, Germany).

Tris-NTA provides a more stable anchorage than the conventional mono-NTA because of its higher affinity for His tags,[11] especially convenient for the desired stable grafting of glycopolymers.
SUVs were prepared by mixing DOPC and DOPE-CAP-B (for biotin presenting SUVs), or DOPC and DODA-(NTA)$_3$ (for NTA-presenting SUVs), at 95:5 molar ratio in chloroform in a glass vial. The mixture was dried under a stream of N_2 gas until a thin film was achieved, and then left to dry further for 2 h under vacuum. The lipids were re-suspended in HBS (10 mM HEPES pH 7.4, 150 mM NaCl) at 2mg/mL, followed by five cycles of vortex, freezing and thawing to obtain a homogeneous multilamellar membrane suspension. The suspension was then sonicated on ice by tip sonication (Fisher Scientific) in pulse mode (1 s on/1 s off) at 30% power (total 15 min active sonication), followed by centrifugation at 12.100 $\times g$ for 10 min to remove titanium particles from the sonicator tip. The SUVs thus obtained were stored in an inert atmosphere (N_2) at 4 °C until use.

Biophysical characterisation of glycocalyx models

Quartz crystal microbalance with dissipation monitoring (QCM-D)

Preparation of sensors and working buffer. Silica coated QCM-D sensors (QSX303, Biolin Scientific, Västra Frölunda, Sweden) were cleaned by immersion for 30 min in 2% sodium dodecyl sulphate (SDS) solution in ultrapure water, rinsing with ultrapure water, blow-drying in N_2 gas, and exposure to UV/ozone (ProCleaner Plus; Bioforce Nanoscience, USA) for 30 min just before use.
HBS working buffer (10 mM HEPES pH 7.4, 150 mM NaCl) was degassed before the experiment, and used for sample dilution to the desired working concentrations just before their injection.

Experimental setup, data acquisition and presentation. QCM-D experiments were performed with 4 independent flow modules using the Q-Sense E4 system (Biolin Scientific). Flow modules were connected to a syringe pump (Legato, World Precision Instruments) to operate in withdraw mode at 20 μL/min. The working temperature was set to 23 °C.
Changes in the sensors' resonance frequency (Δf_i) and dissipation (ΔD_i) were continuously recorded for six overtones (i = 3, 5, 7, 9, 11 and 13, corresponding to resonance frequencies of approximately 15, 25, 35, 45, 55 and 65 MHz) with sub-second time resolution, using the instrument's data acquisition software QSoft.
Whilst data for i = 5 are typically presented, all other overtones gave qualitatively similar information. Normalised frequency shifts, $\Delta F = \Delta f_i / i$, are presented throughout.

Quantification of film thickness from QCM-D data. The thickness of SLBs and dense SAv monolayers was estimated from the QCM-D frequency shift using the Sauerbrey equation as $h = -C\Delta F/\rho$, with the mass-sensitivity constant C = 18.0 ng/(cm^2 Hz). The film density was assumed to be 1.0 g/cm^3 for lipids, and 1.1 g/cm^3 for SAv, reflecting the solvated nature of the film, to a good approximation. We verified that $\Delta D/-\Delta F \ll 0.4\ 10^{-6}$/Hz to ascertain that films are sufficiently rigid for the Sauerbrey equation to provide reliable film thickness estimates.[12]
Glycopolymer brushes (plain or with bound B_5 subunits) were too soft for the Sauerbrey equation to provide accurate film thickness data. The brush thickness was therefore quantified through viscoelastic modelling,[12,13] using the 'small-load approximation' model in PyQTM.[14,15] The bulk solution was treated as a Newtonian fluid with viscosity η = 0.89 mPa·s. The brush was approximated as a mechanically isotropic film with thickness h, density ρ, and frequency-dependent shear storage modulus ($G'(f) = G_0'(f/f_0)^{\beta'}$) and shear loss modulus ($G''(f) = G_0''(f/f_0)^{\beta''}$). The reference frequency pertinent to G_0' and G_0'' was arbitrarily set to f_0 = 15 MHz, and the powers β' and β'' were taken to be constant over the relatively narrow frequency range covered by QCM-D. Data from all six overtones were used for viscoelastic modelling.[12] h, G_0', β' and G_0'' were treated as adjustable parameters, whilst ρ and β'' were fixed. Considering the solvated nature of the glycopolymer brushes, we fixed ρ = 1.0 g/cm^3 to a good approximation. We also fixed β'' = 0.9, as this provided more robust data for the four remaining adjustable parameters, and as preliminary fits including β'' into the set of adjustable parameters showed values for this parameter scattered around 0.9 for the various brushes. Presented data represent the best-fit value ± confidence intervals as computed by PyQTM.

Spectroscopic ellipsometry (SE)

Ellipsometry measures changes in the polarisation of light, specifically, the ellipsometric angles Δ and ψ, upon reflection at the surface of interest. As the polarisation of light is very sensitive to changes in the optical properties of the surface, it can be used to characterize the thickness and refractive index of thin organic surface coatings. From these parameters, the surface density of molecules can be quantified.

Preparation of the sensing surface and working buffer. Pieces of silicon wafer with dimensions 3 mm × 20 mm and a thin native oxide coating (BT Electronics, Les Ulis, France) were used as sensing surfaces. Prior to use, the wafer pieces were cleaned as described for QCM-D sensors. HBS working buffer was degassed before the experiment.

Experimental setup. Experiments were performed with a spectroscopic rotating compensator ellipsometer (M2000V; J.A. Woollam; NE, USA) with a horizontal plane of light incidence. Ellipsometric data were acquired over a range of wavelengths λ, from 380 to 1000 nm, at an angle of incidence of 70 degrees, and a time resolution of ~5 s. CompleteEASE software (J. A. Woollam) was used for data acquisition and analysis.
A custom-built open cuvette (~100 µL volume) with glass windows was used for the real time *in situ* analysis of binding processes.[16] Samples (in working buffer) were directly pipetted into the cuvette and homogenized by a magnetic stirrer located at the bottom of the cuvette. SUVs were incubated under continuous stirring. All other samples were stirred for approximately 5 s after sample injection; for the remainder of the sample incubation time the stirrer was turned off and adsorption left to proceed from stagnant solution. To remove sample, working buffer was flowed through the cuvette, assisted by a flow-through tubing system and a peristaltic pump (IPC; Ismatec, Germany) operated at a flow rate of 5 mL/min; during these phases, the stirrer was turned on to ensure homogenization and maximize exchange of the cuvette content.

Quantification of areal mass density from SE data. A model with a stack of multiple optically isotropic layers relates the measured ellipsometric angles Δ and Ψ as a function of λ to the optical properties of the substrate, the adsorbed biomolecular films, and the surrounding buffer solution.
The semi-infinite bulk solution was treated as a transparent Cauchy medium (refractive index: $n_{sol}(\lambda) = A_{sol} + B_{sol}/\lambda^2$, where A_{sol} = 1.325 and B_{sol} = 0.00322 µm^2.[17]
The native oxide film on the Si wafers was modeled as a single and transparent Cauchy layer. Its optical properties were determined from the measurements acquired in the presence of bulk solution but in the absence of the biomolecular film, which were then fitted over the range of λ using the tabulated values for the underlying Si substrate (implemented in CompleteEASE). To this end, B_{SiO2} = 0.0159 was fixed and the thickness h_{SiO2} and optical parameter A_{SiO2} of the silicon oxide coating, and the angle of incidence, where adjustable parameters. Typical resulting values were A_{SiO2} = 2.411 ± 0.062 and h_{SiO2} = 1.01 ± 0.17 nm, with an error in the angle of incidence below 0.1 degrees. The normalized χ^2 value was lower than 1.6, that is close to 1, indicating a fit of good quality. The oxide film parameters and angle of incidence were subsequently fixed when analyzing biomolecular binding to the surface.
The adsorbed biomolecular film was fitted as two separate layers. The SLB (plain or with a monolayer of streptavidin) was considered a first layer (index 1), which was treated as a transparent Cauchy medium with thickness h_1 and a wavelength-dependent refractive index $n_1(\lambda) = A_1 + B_1/\lambda^2$. The glycopolymer brush (plain or with bound toxin B subunits), was treated as a separate transparent Cauchy medium (index 2) with thickness h_2 and $n_2(\lambda) = A_2 + B_2/\lambda^2$. We set, $B_{sol} = B_1 = B_2$, recognising that the wavelength dispersion of the solvated layers is not sensitively affected by the biomolecules. During the processes of SLB and SAv monolayer formation, the thickness h_2 was set to zero, and A_1 and h_1 were the adjustable fit parameters. Layer 1 was then assumed to remain unchanged, *i.e.*, A_1 and h_1 were fixed and A_2 and h_2 were the adjustable fit parameters, during the subsequent glycopolymer and toxin B subunit binding processes. The χ^2 values for the fits were typically below 2.0, indicating a fit of high quality, although occasionally (for some sample injections) a moderately higher value was transiently obtained due to light scattering during mixing of solutions with distinct refractive indices.
*AMD*s were determined through a variant of de Fejter's equation,[18] $AMD = h(A - A_{sol})/(dn/dc)$, using the refractive index increments, dn/dc, of 0.18 cm^3/g for all proteins, 0.15 cm^3/g for all glycopolymers, and 0.169 cm^3/g for all lipids.
Molar surface densities Γ or number average molecular masses M_n were obtained from the *AMD* through the equation $AMD = \Gamma \times M_n$, with known molecular mass or molar surface density, respectively. Errors in *AMD* (and Γ or M_n) comprise the temporal noise and the confidence intervals of the data fitting.

Calculating other physiochemical properties of the glycocalyx models

Concentration of target glycans in model glycocalyces. c_{glycan} was determined from the areal mass density *AMD* and the thickness h of the glycopolymer film, and the molecular mass per target glycan moiety in the glycopolymer ($M_{HA\text{-glycan fragment}} = M_{HA\ 1ds}/DS + M_{glycan}$, with $M_{HA\ 1ds}$ = 400 Da the molecular mass per HA disaccharide and M_{glycan} the molecular mass added to the HA chain upon conjugation of one target glycan moiety) as $c_{glycan} = AMD/(h \times M_{HA\text{-glycan fragment}})$. Here the thickness (plain glycopolymers or with bound toxin B subunits) determined by viscoelastic modelling of QCM-D was used, as this provided a more robust information on glycopolymer film thickness than the apparent thickness obtained by SE.

Root-mean-square distance of target glycans in model glycocalyces. $d_{\mathrm{rms,glycan}}$ was determined from the target glycan concentration and Avogadro's number as $d_{\mathrm{rms,glycan}} = (c_{\mathrm{glycan}} \times N_{\mathrm{A}})^{-1/3}$.

Root-mean-square distance of glycopolymer anchor sites. $d_{\mathrm{rms,gp\ anchor}}$ was determined from the molar glycopolymer surface density Γ_{gp} and Avogadro's number as $d_{\mathrm{rms,gp\ anchor}} = (\Gamma_{\mathrm{gp}} \times N_{\mathrm{A}})^{-1/2}$.

Contour length of glycopolymers. L_{c} (along the HA backbone) was determined from the molecular mass of the glycopolymer (M_{gp}), the contour length per HA disaccharide ($L_{\mathrm{c,ds}}$ = 1 nm) the degree of substitution (DS) and $M_{\mathrm{HA\text{-}glycan\ fragment}}$, as $L_{\mathrm{c}} = M_{\mathrm{gp}}/(M_{\mathrm{HA\text{-}glycan\ fragment}}) \times L_{\mathrm{c,ds}}/DS$.

APPENDIX

NMR data

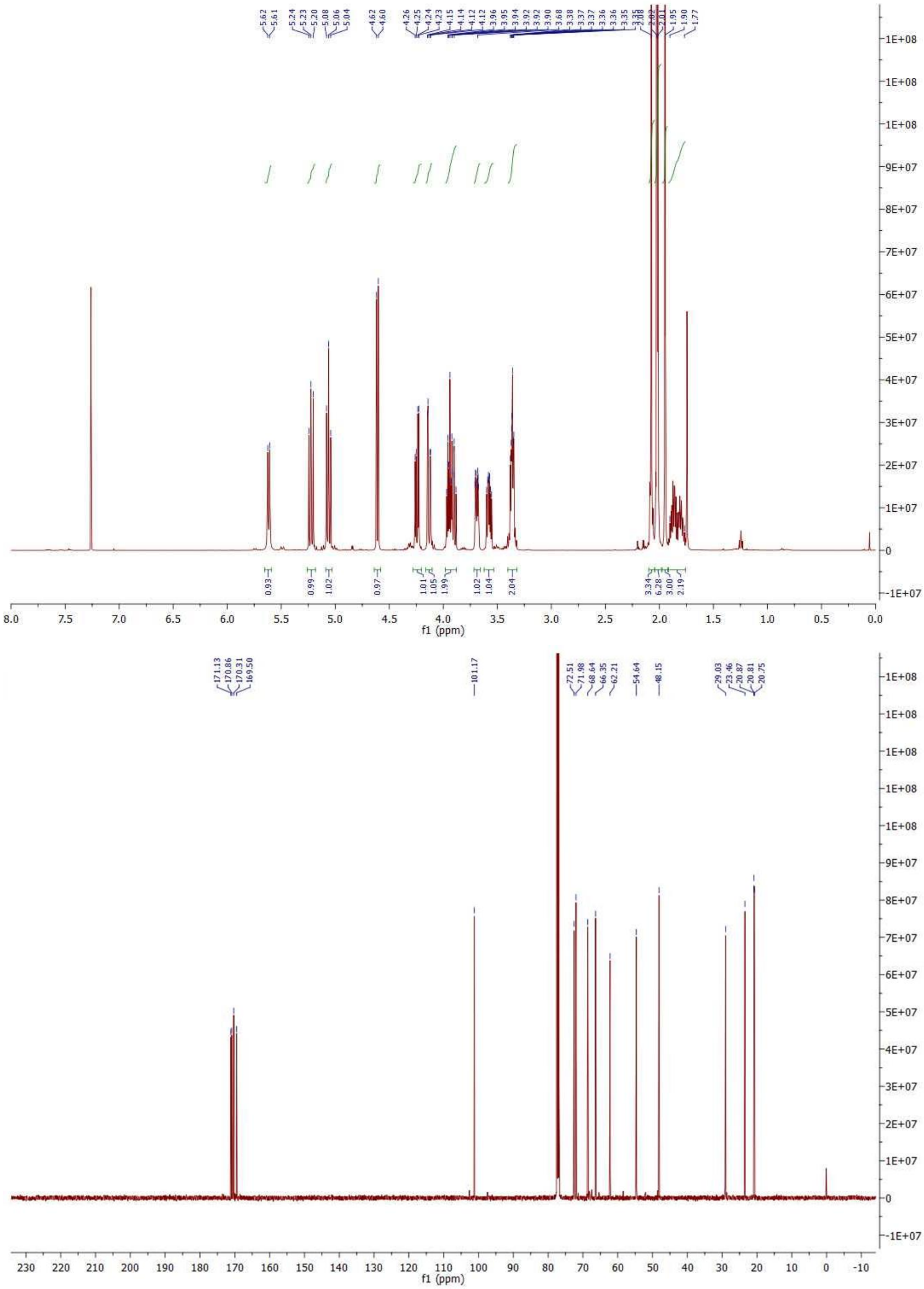

Figure S18: ^{1}H NMR (top) and ^{13}C NMR (bottom) spectra of 3-azidopropyl 2-acetamido-3,4,6-tri-O-acetyl-β-d-glucopyranoside.

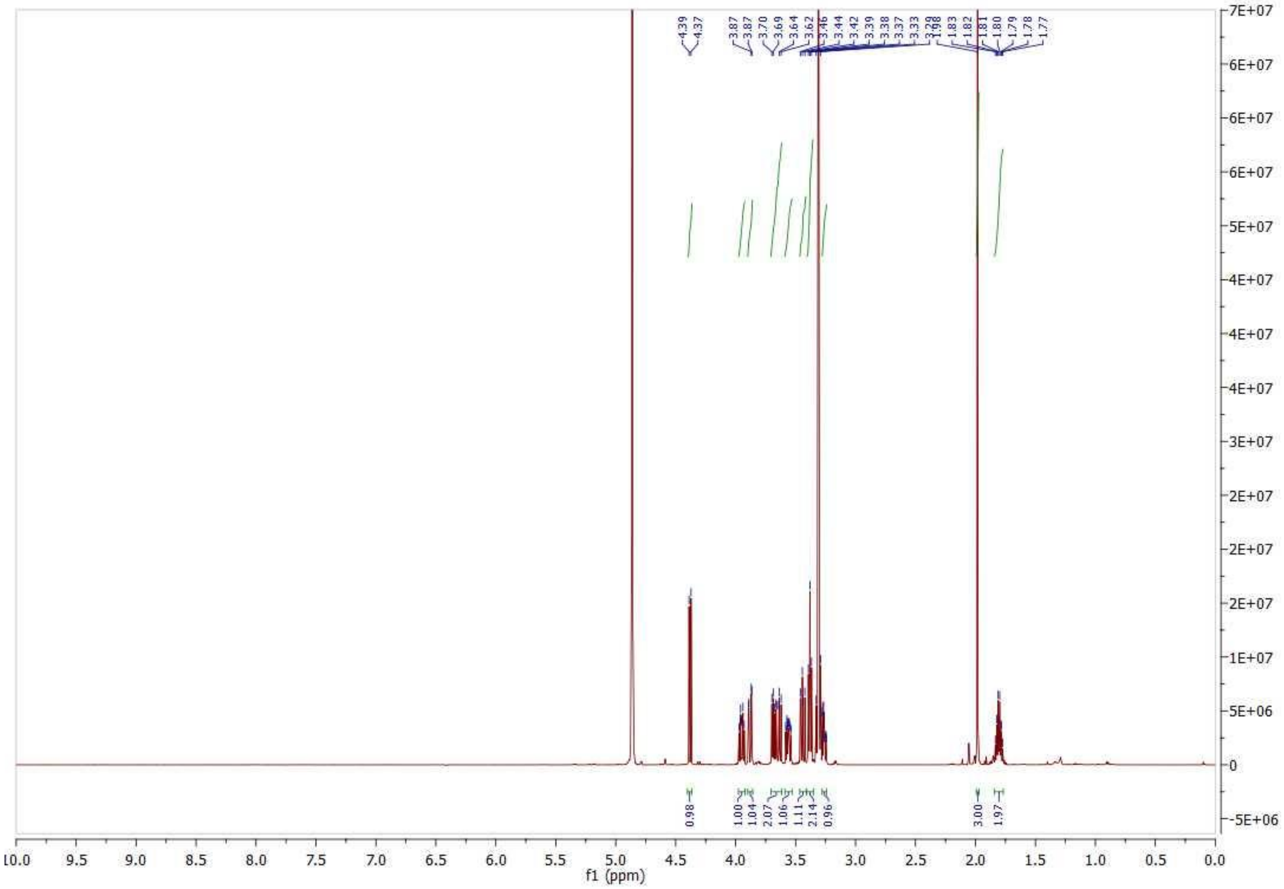

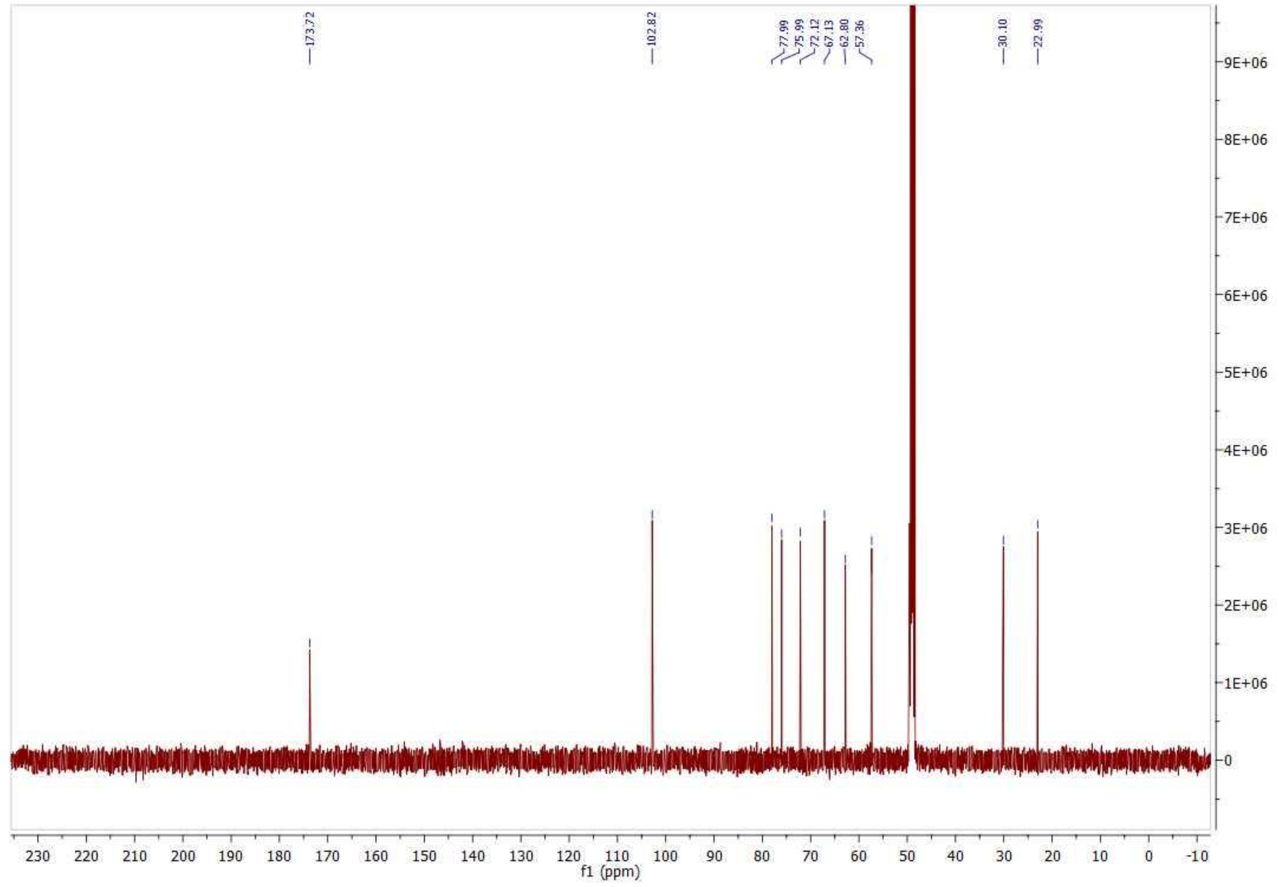

Figure S19: ^1H NMR (top) and ^{13}H NMR (bottom) spectra of 3-azidopropyl *N*-acetamido-β-D-glucopyranoside (**1**).

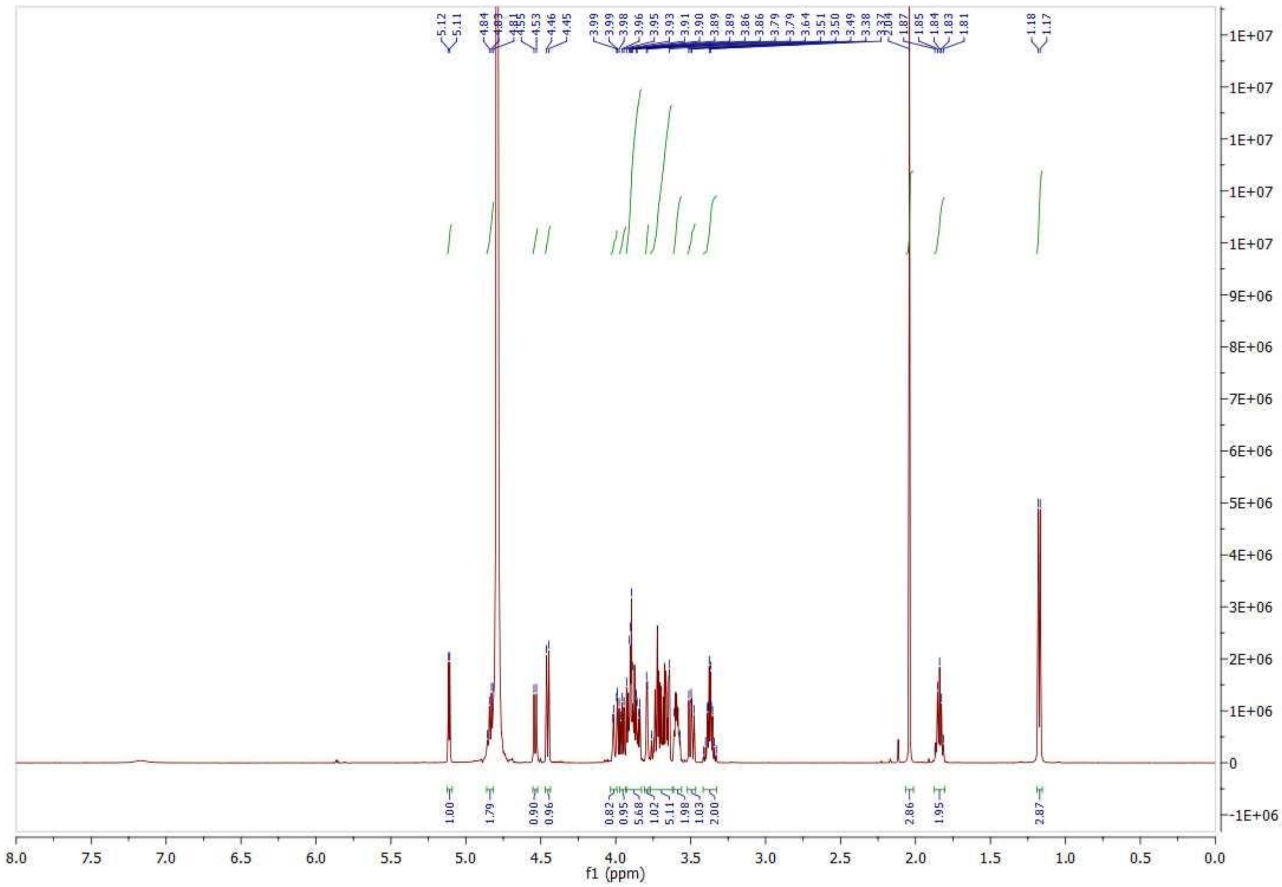

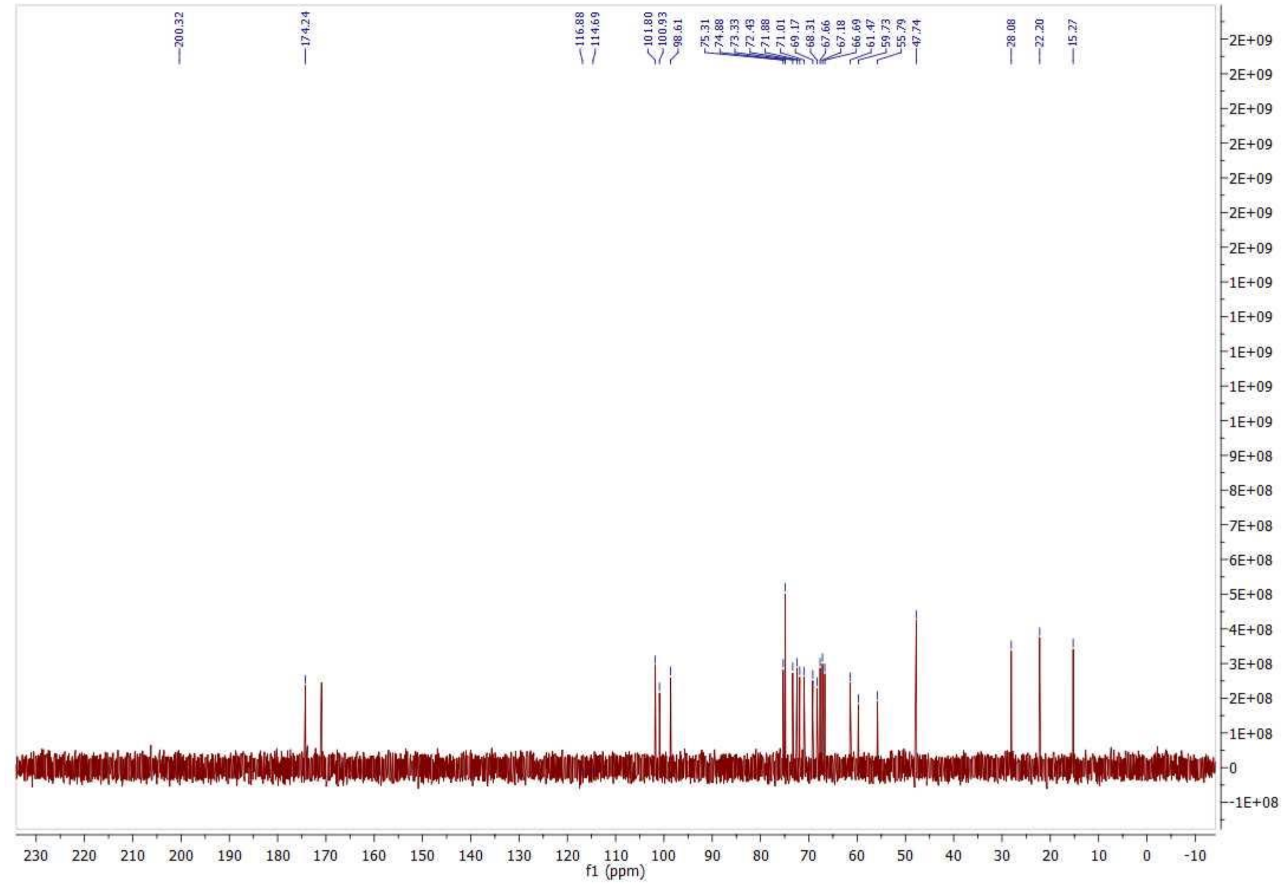

Figure S20: ^{1}H NMR (top) and ^{13}C NMR (bottom) spectra of azidopropyl Le^{x} (**3**).

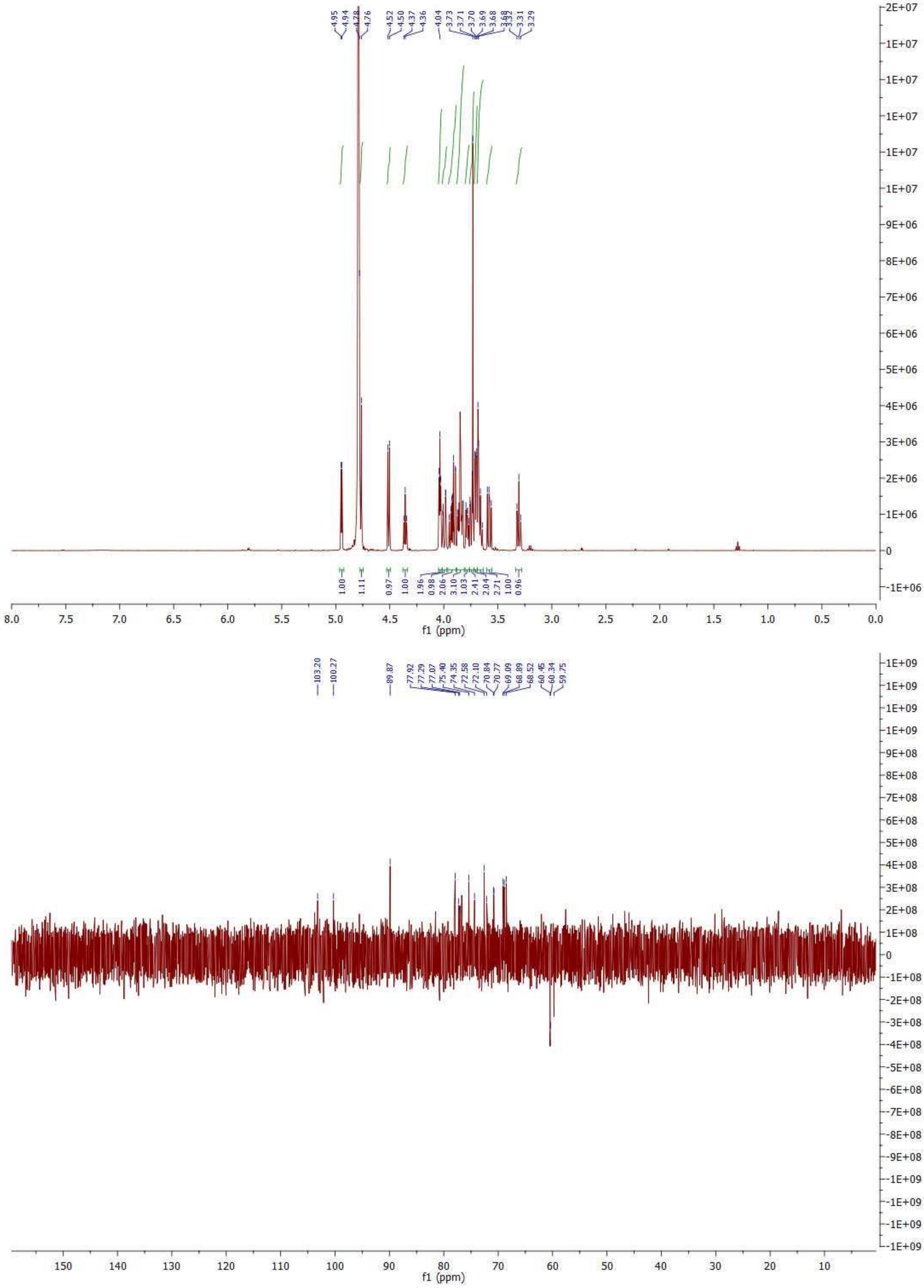

Figure S21: ^{1}H NMR (top) and ^{13}C NMR (bottom) spectra of Gb_3-N_3 (**5**).

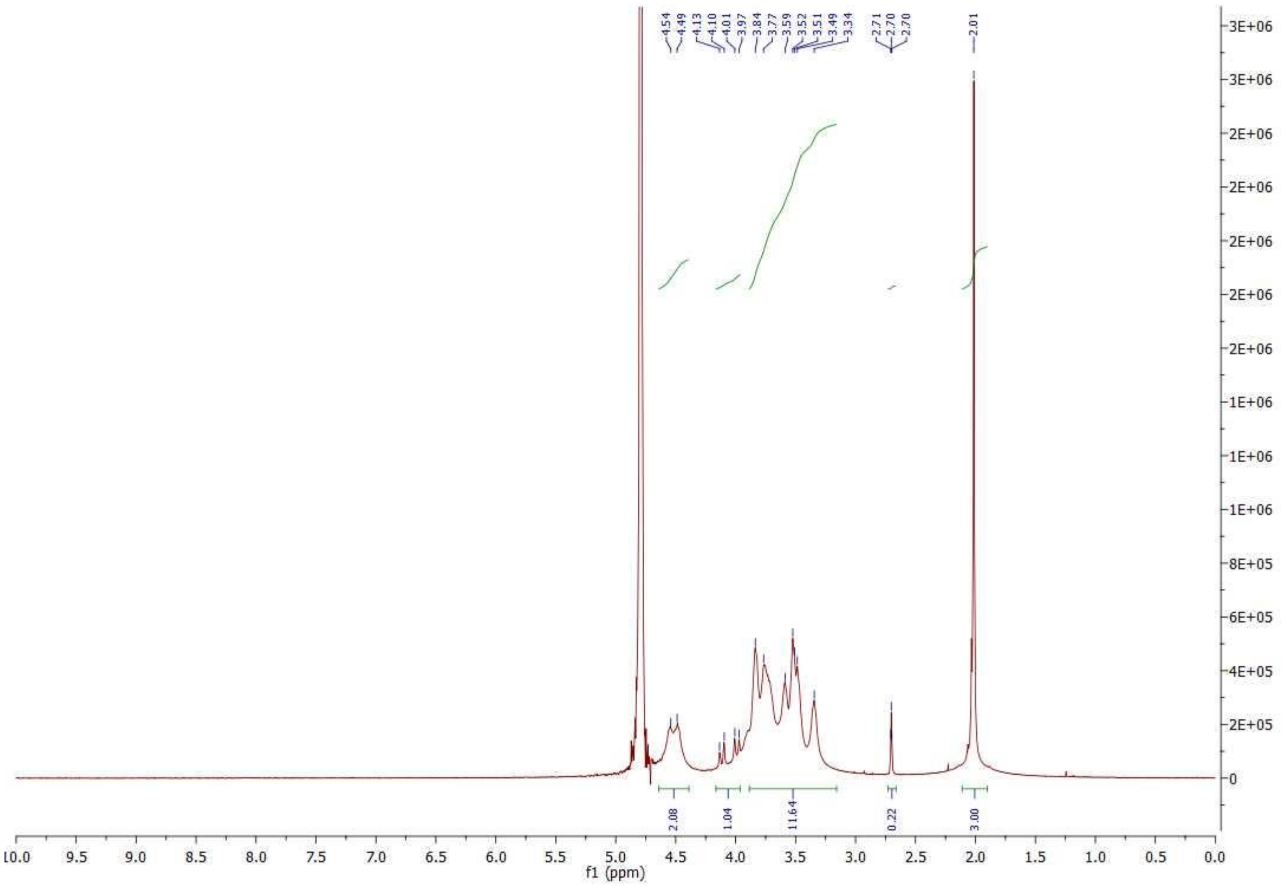

Figure S22: ^{1}H NMR spectrum of HA-*g*-Propargyl **6** (Test 1; *DS* = 22%).

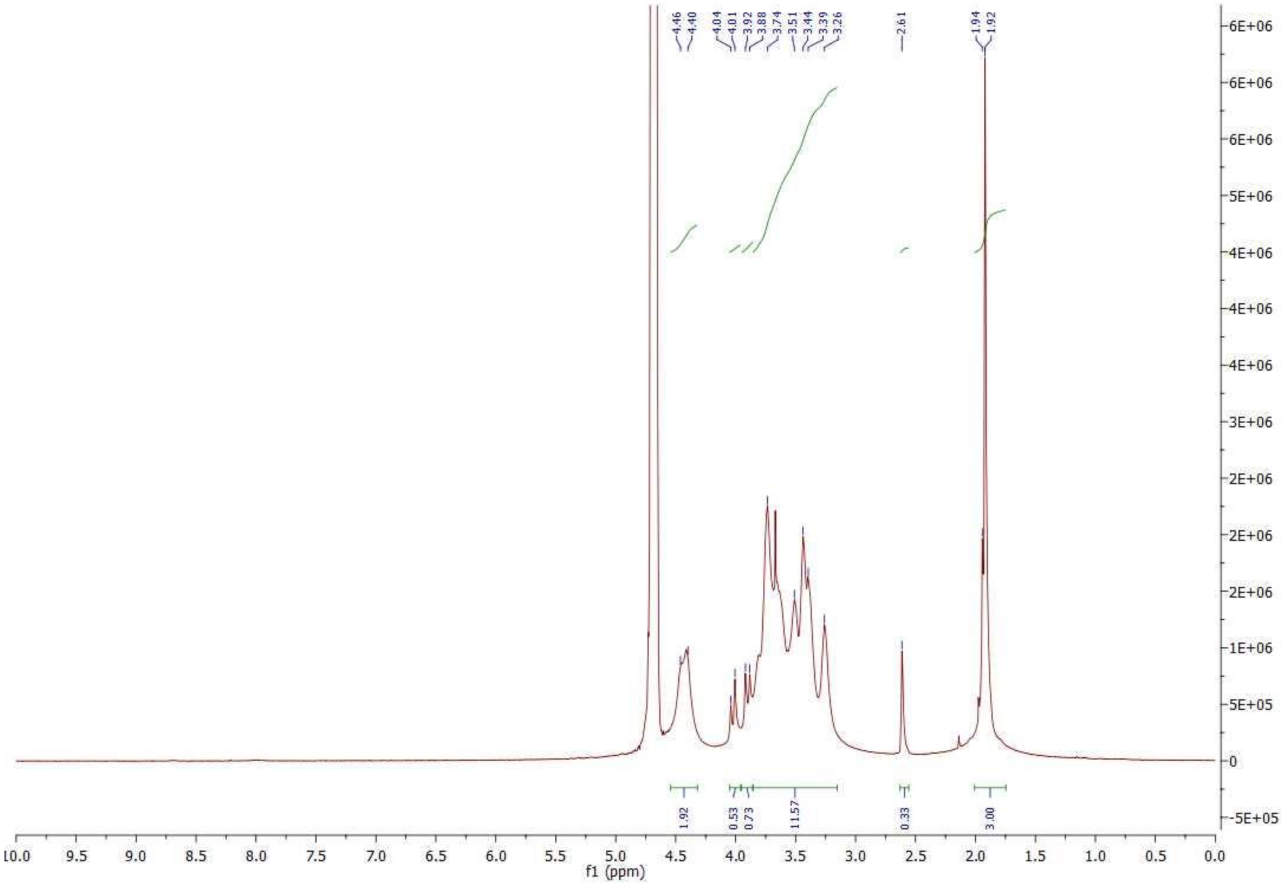

Figure S23: ^{1}H NMR spectrum of HA-*g*-Propargyl **6** (Test 2; *DS* = 33%).

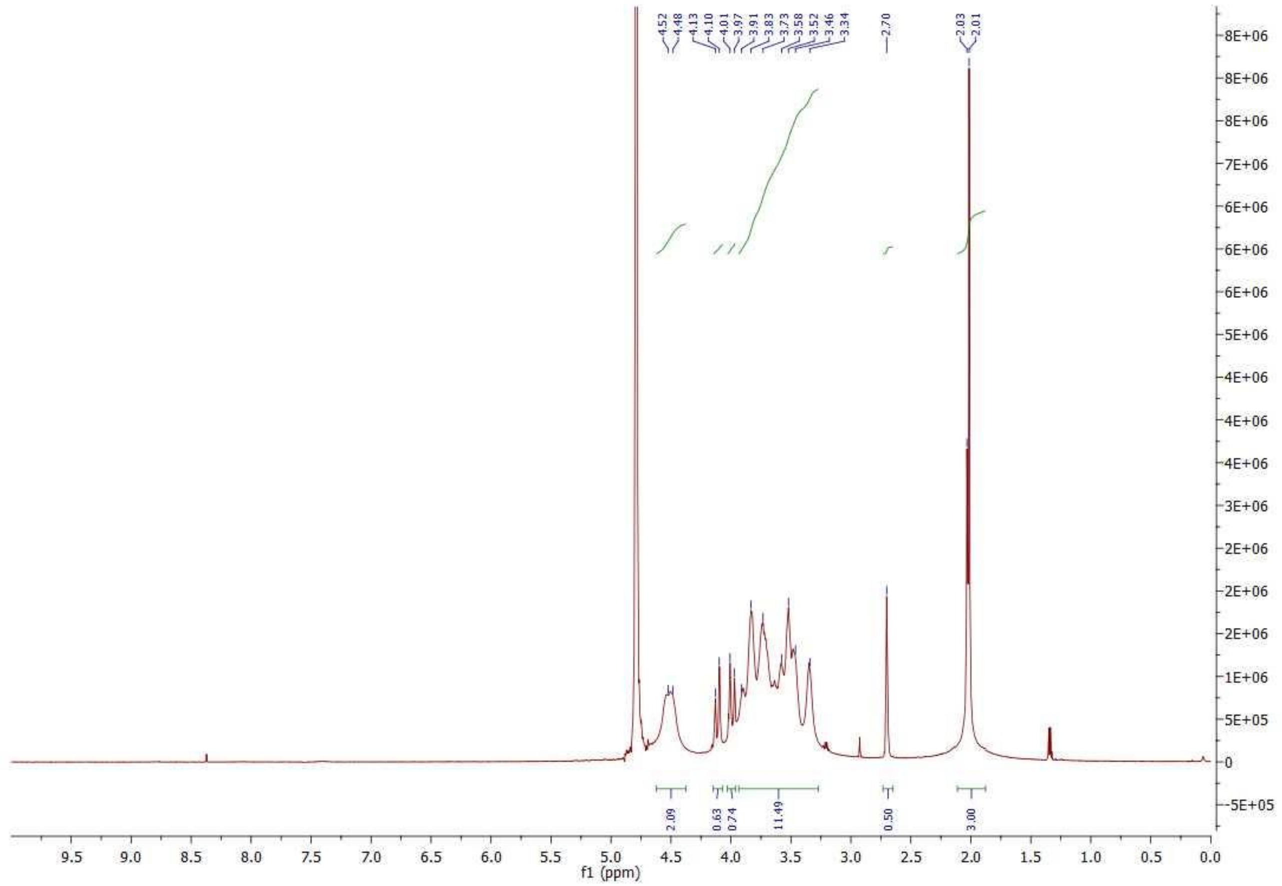

Figure S24: ^{1}H NMR spectrum of HA-*g*-Propargyl **6** (Test 3; *DS* = 50%).

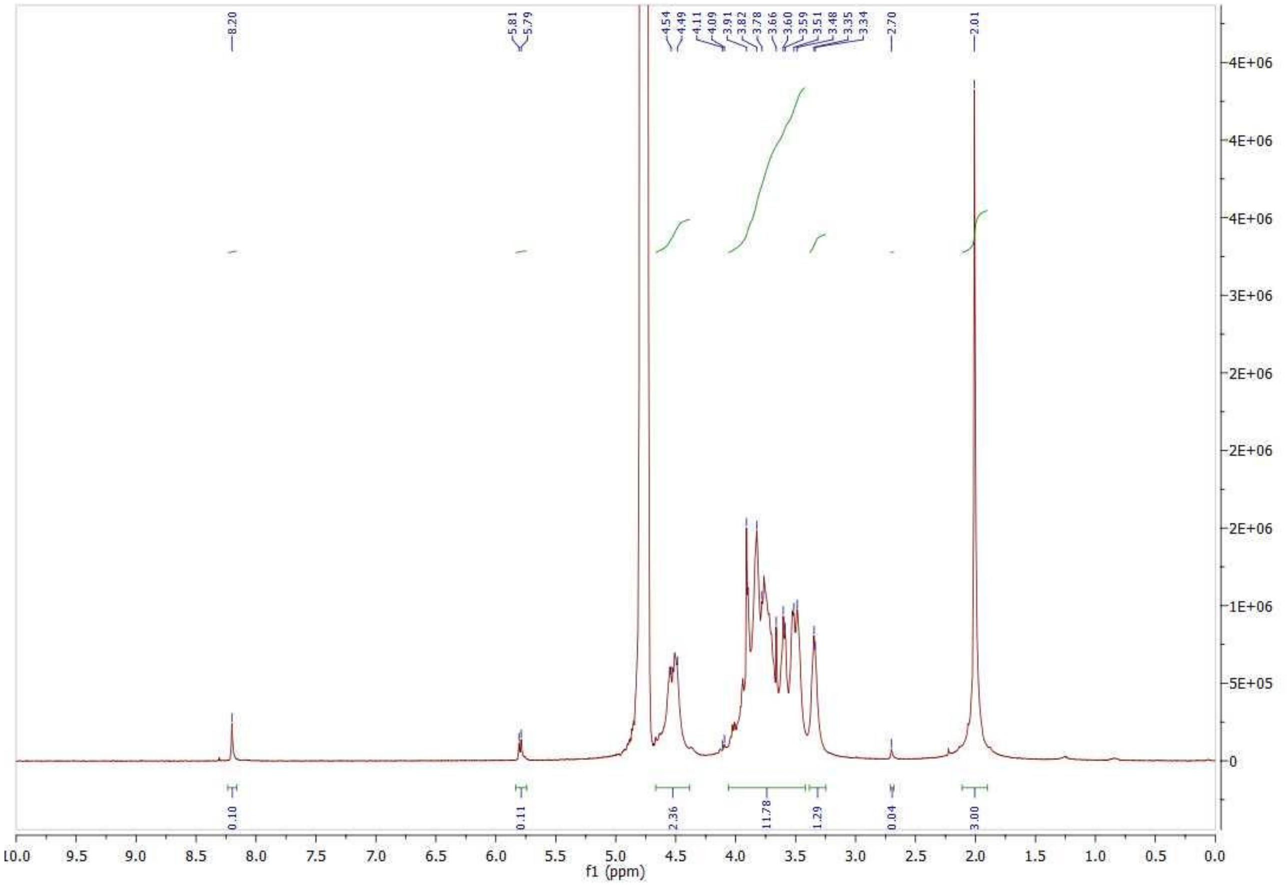

Figure S25: ^{1}H NMR spectrum of HA-g-Lac^L.

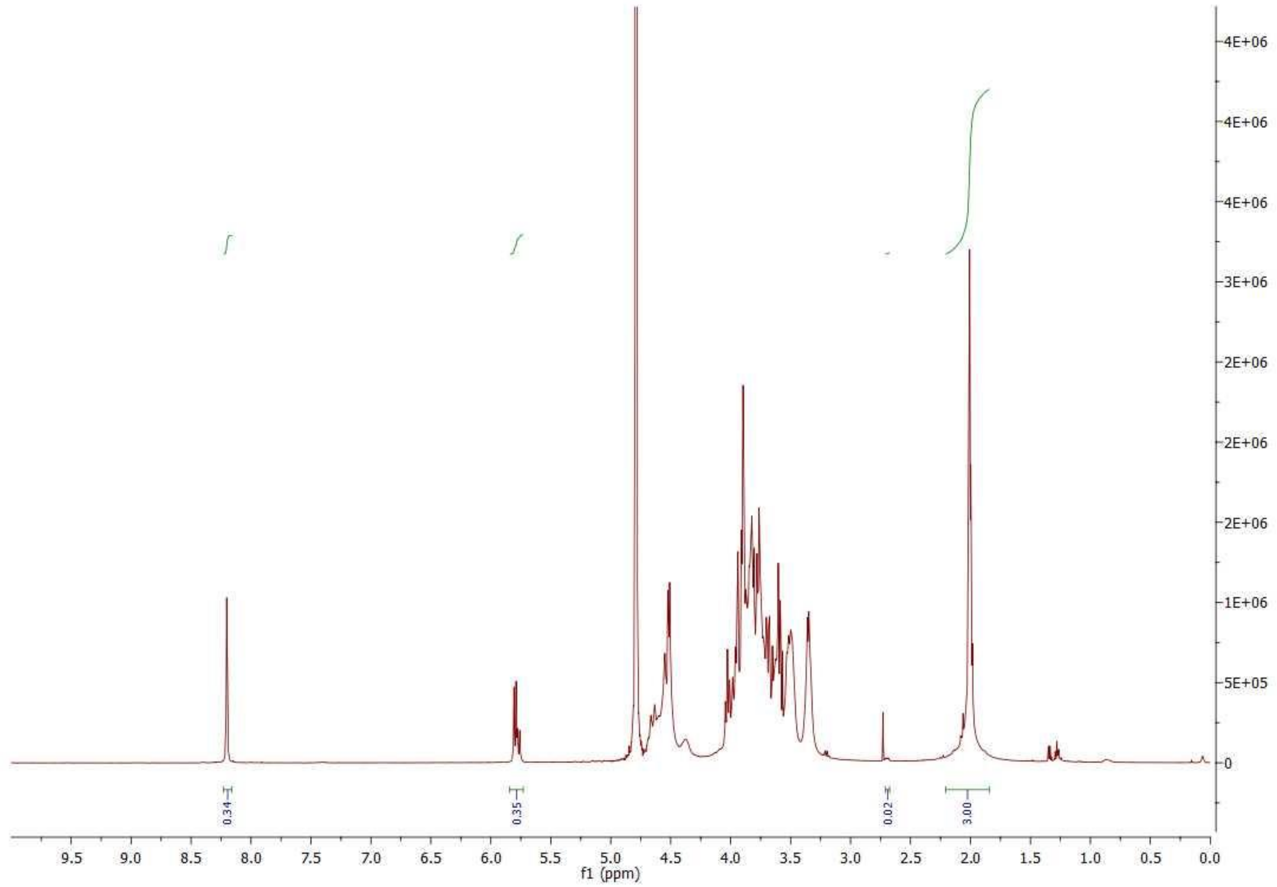

Figure S26: ^{1}H NMR spectrum of HA-g-Lac^{H}.

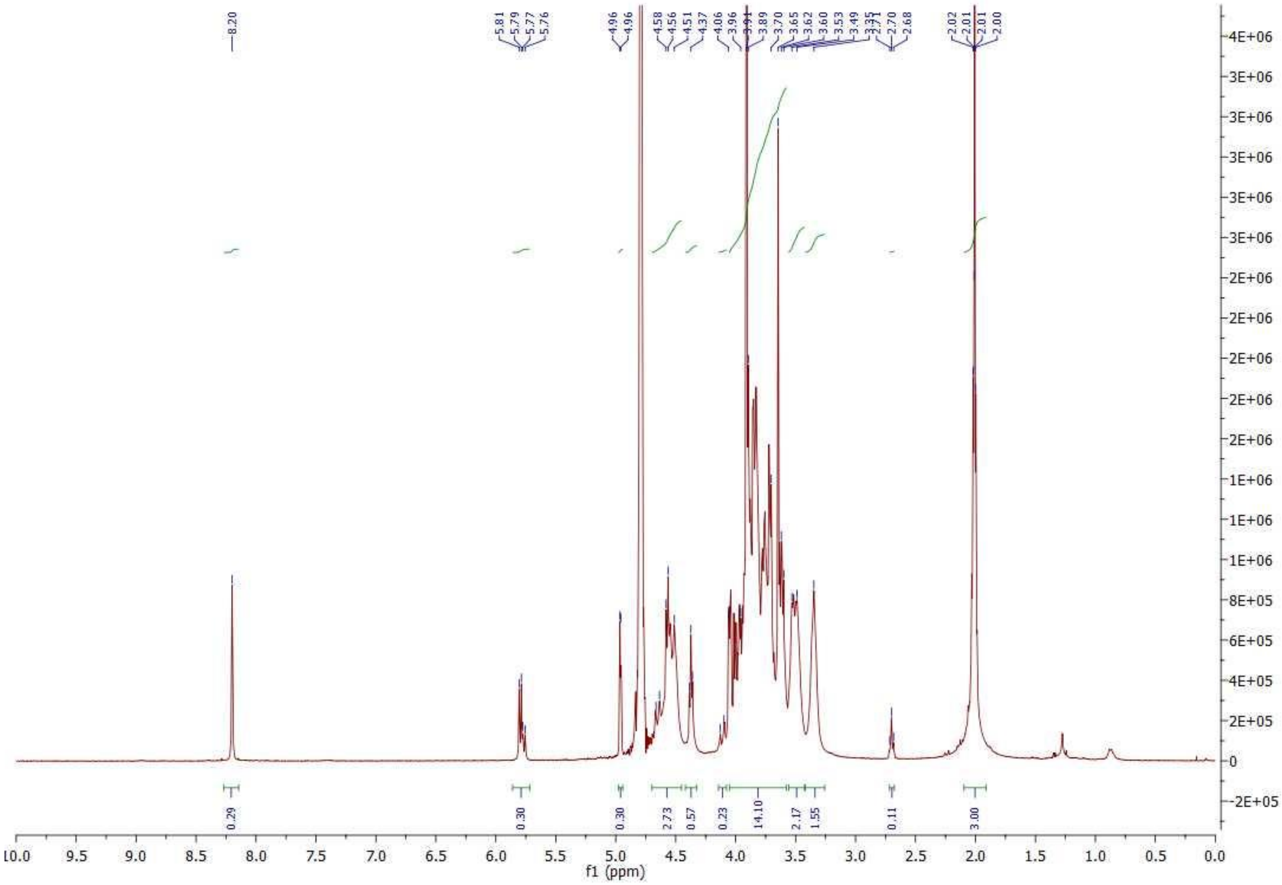

Figure S27: ^{1}H NMR spectrum of HA-g-Gb$_3$.

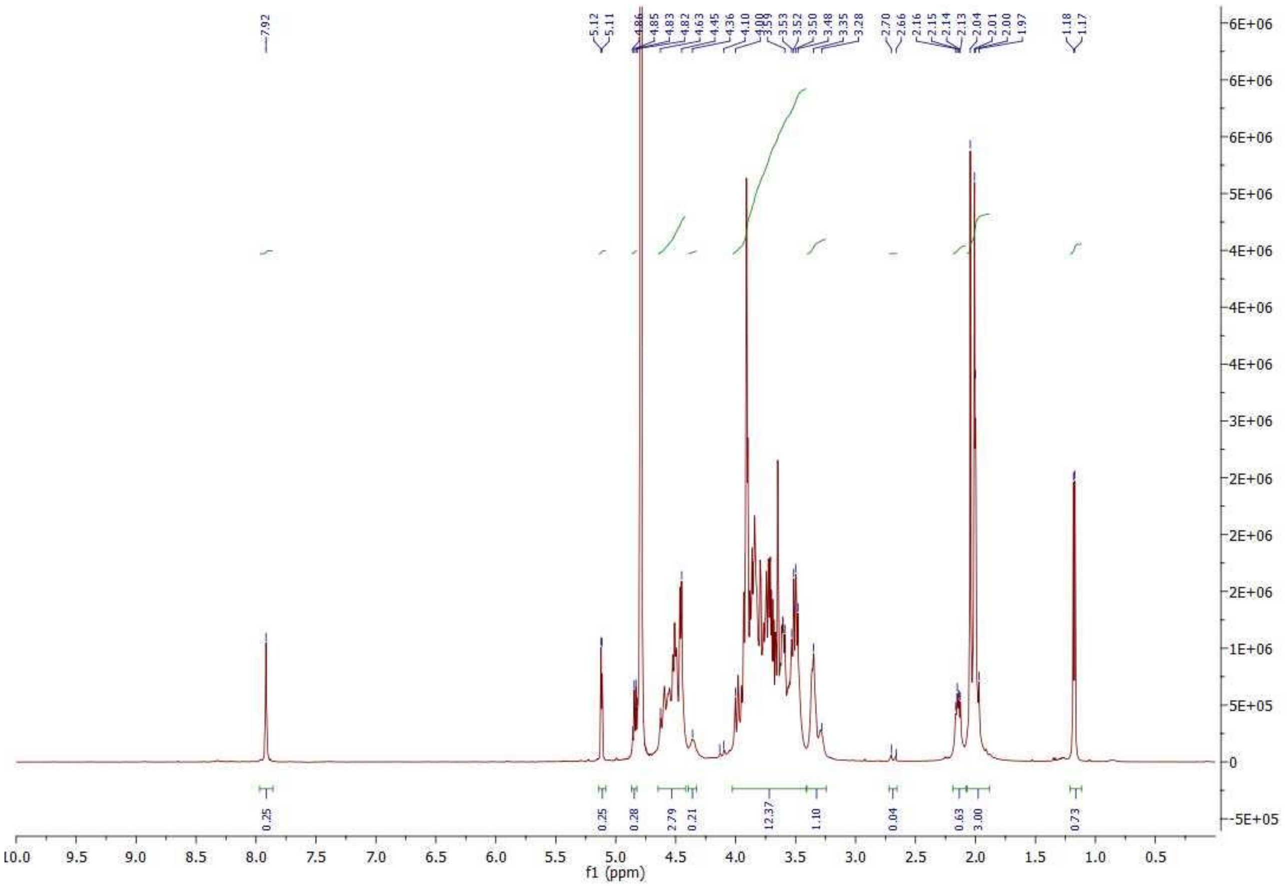

Figure S28: ^{1}H NMR spectrum of HA-g-Le^{x}.

SEC-MALS data

All size exclusion chromatograms show the light scattering intensity (I_{LS}; black solid line) and the refractive index increase (Δn; compared to plain HEPES buffer; red dashed line) normalized by the maximal values ($I_{LS,max}$ and Δn_{max}), and the molar mass (purple symbols) as a function of the elution volume.

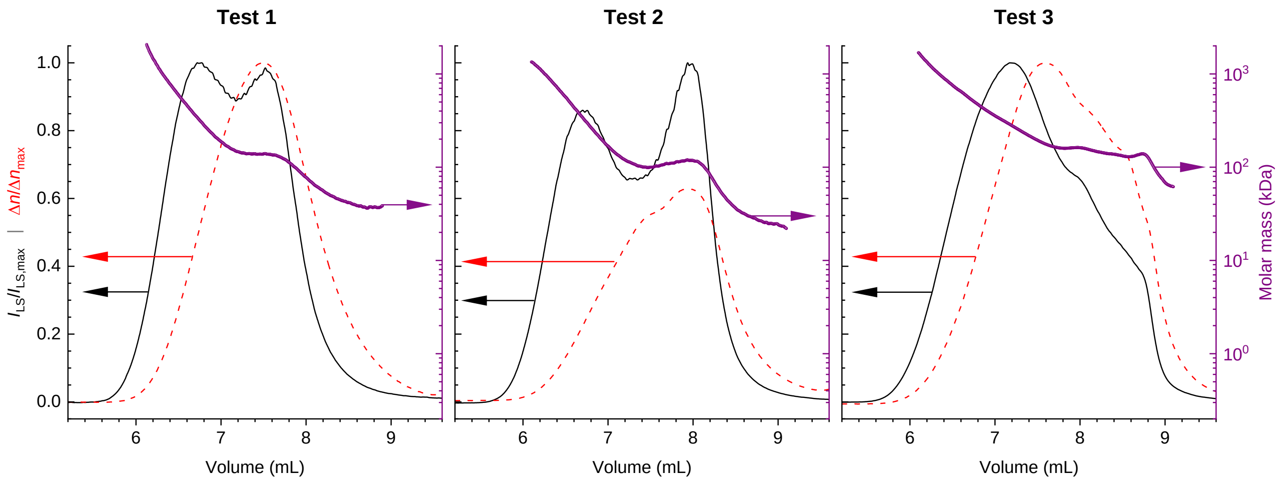

Figure S29: HA-*g*-Propargyl **6** (Test 1-3, as indicated above the graphs).

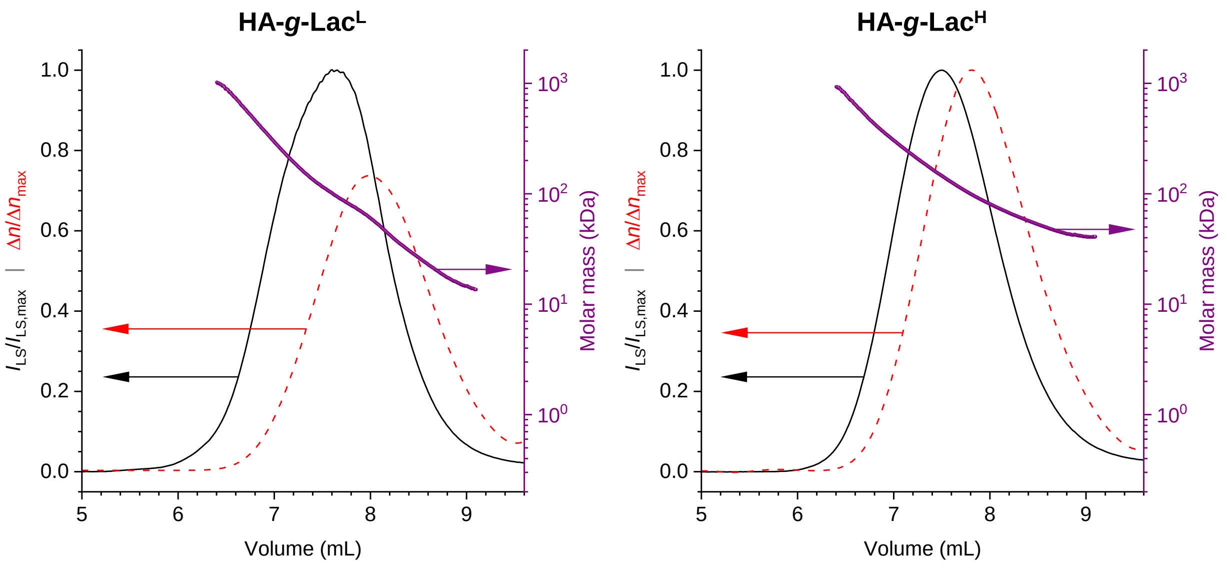

Figure S30: HA-*g*-Lac^L(left) and HA-*g*-Lac^H (right).

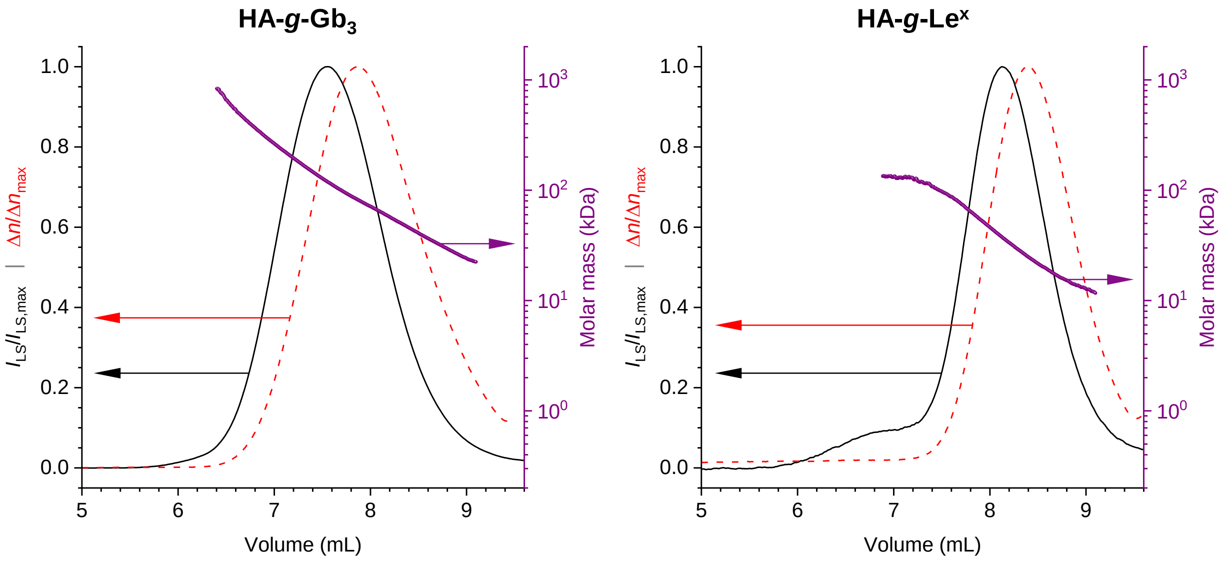

Figure S31: HA-*g*-Gb_3. (left) and HA-*g*-Le^x (right).

SUPPORTING REFERENCES

(1) Gottlieb, H. E.; Kotlyar, V.; Nudelman, A. NMR Chemical Shifts of Common Laboratory Solvents as Trace Impurities. *J. Org. Chem.* **1997**, *62*, 7512–7515. https://doi.org/10.1109/TPS.2011.2168830.

(2) Hollósi, M.; Kollát, E.; Laczkó, I.; Medzihradszky, K. F.; Thurina, J.; Otvös Jr., L. SSolid-phase synthesis of glycopeptides: Glycosylation of resin-bound serine-peptides by 3,4,6-tri-o-acetyl-D-glucose-oxazoline. *Tetrahedron Lett.* **1991**, *32* (12), 1531–1534.

(3) Srivastava, V. K. A facile synthesis of 2-methyl-(3,4,6-tri-O-acetyl-1,2-dideoxy-α-D-glucopyrano)- [2,1-e]-oxazoline. *Carbohydr. Res.* **1982**, *103*, 286–292.

(4) Willems, M. M. J. H. P.; Zom, G. G.; Meeuwenoord, N.; Ossendorp, F. A.; Overkleeft, H. S.; Marel, G. A. Van Der; Codée, J. D. C.; Filippov, D. V. Design , automated synthesis and immunological evaluation of NOD2-ligand – antigen conjugates. *Beilstein J. Org. Chem.* **2014**, *10*, 1445–1453. https://doi.org/10.3762/bjoc.10.148.

(5) Valverde, P.; Vendeville, J. B.; Hollingsworth, K.; Mattey, A. P.; Keenan, T.; Chidwick, H.; Ledru, H.; Huonnic, K.; Huang, K.; Light, M. E.; Turner, N.; Jiménez-Barbero, J.; Galan, M. C.; Fascione, M. A.; Flitsch, S.; Turnbull, W. B.; Linclau, B. Chemoenzymatic synthesis of 3-deoxy-3-fluoro-l-fucose and its enzymatic incorporation into glycoconjugates. *Chem. Commun.* **2020**, *56* (47), 6408–6411. https://doi.org/10.1039/d0cc02209h.

(6) Nam, Y. W.; Nishimoto, M.; Arakawa, T.; Kitaoka, M.; Fushinobu, S. Structural basis for broad substrate specificity of UDP-glucose 4-epimerase in the human milk oligosaccharide catabolic pathway of Bifidobacterium longum. *Sci. Rep.* **2019**, *9* (1), 11081. https://doi.org/10.1038/s41598-019-47591-w.

(7) Hollingsworth, K.; Di Maio, A.; Richards, S.-J.; Vendeville, J.-B.; Wheatley, D. E.; Council, C. E.; Keenan, T.; Ledru, H.; Chidwick, H.; Huang, K.; Parmeggiani, F.; Marchesi, A.; Chai, W.; McBerney, R.; Kamiński, T. P.; Balmforth, M. R.; Tamasanu, A.; Finnigan, J. D.; Young, C.; Warriner, S. L.; Webb, M. E.; Fascione, M. A.; Flitsch, S.; Galan, M. C.; Feizi, T.; Gibson, M. I.; Liu, Y.; Turnbull, W. B.; Linclau, B. Synthesis and screening of a library of Lewisx deoxyfluoro-analogues reveals differential recognition by glycan-binding partners. *Nat. Commun.* **2024**, *15* (1), 7925. https://doi.org/10.1038/s41467-024-51081-7.

(8) Pasupuleti, R.; Riedl, S.; Saltor Núñez, L.; Karava, M.; Kumar, V.; Kourist, R.; Turnbull, W. B.; Zweytick, D.; Wiltschi, B. Lectin-anticancer peptide fusion demonstrates a significant cancer-cell-selective cytotoxic effect and inspires the production of "clickable" anticancer peptide in Escherichia coli. *Protein Sci.* **2023**, *32* (12), 1–14. https://doi.org/10.1002/pro.4830.

(9) Kirichuk, O.; Srimasorn, S.; Zhang, X.; Roberts, A. R. E.; Coche-Guerente, L.; Kwok, J. C. F.; Bureau, L.; Débarre, D.; Richter, R. P. Competitive Specific Anchorage of Molecules onto Surfaces: Quantitative Control of Grafting Densities and Contamination by Free Anchors. *Langmuir* **2023**, *39* (50), 18410–18423. https://doi.org/10.1021/acs.langmuir.3c02567.

(10) Beutel, O.; Nikolaus, J.; Birkholz, O.; You, C.; Schmidt, T.; Herrmann, A.; Piehler, J. High-fidelity protein targeting into membrane lipid microdomains in living cells. *Angew. Chem. Int. Ed.* **2014**, *53* (5), 1311–1315. https://doi.org/10.1002/anie.201306328.

(11) Lata, S.; Reichel, A.; Brock, R.; Tampé, R.; Piehler, J. High-affinity adaptors for switchable recognition of histidine-tagged proteins. *J. Am. Chem. Soc.* **2005**, *127* (29), 10205–10215. https://doi.org/10.1021/ja050690c.

(12) Reviakine, I.; Johannsmann, D.; Richter, R. P. Hearing what you cannot see and visualizing what you hear: Interpreting quartz crystal microbalance data from solvated interfaces. *Anal. Chem.* **2011**, *83* (23), 8838–8848. https://doi.org/10.1021/ac201778h.

(13) Eisele, N. B.; Andersson, F. I.; Frey, S.; Richter, R. P. Viscoelasticity of thin biomolecular films: A case study on nucleoporin phenylalanine-glycine repeats grafted to a histidine-tag capturing QCM-D sensor. *Biomacromolecules* **2012**, *13* (8), 2322–2332. https://doi.org/10.1021/bm300577s.

(14) Johannsmann, D.; Langhoff, A.; Leppin, C.; Reviakine, I.; Maan, A. M. C. Effect of Noise on Determining Ultrathin-Film Parameters from QCM-D Data with the Viscoelastic Model. *Sensors* **2023**, *23* (3). https://doi.org/10.3390/s23031348.

(15) DJohannsmann. *https://github.com/DJohannsmann/QCM-D-Modelling-PyQTM*.

(16) Eisele, N. B.; Frey, S.; Piehler, J.; Görlich, D.; Richter, R. P. Ultrathin nucleoporin phenylalanine-glycine repeat films and their interaction with nuclear transport receptors. *EMBO Rep.* **2010**, *11* (5), 366–372. https://doi.org/10.1038/embor.2010.34.

(17) Carton, I.; Brisson, A. R.; Richter, R. P. Label-Free Detection of Clustering of Membrane-Bound Proteins. *Anal. Chem.* **2010**, *82* (22), 9275–9281.

(18) Richter, R. P.; Rodenhousen, K. B.; Eisele, N. B.; Schubert, M. Chapter 11 Coupling Spectroscopic Ellipsometry and Quartz Crystal Microbalance to Study Organic Films at the Solid-Liquid Interface. in *Ellipsometry of Functional Organic Surfaces and Films*; Hinrichs, K., Eichhorn, K., Eds.; Springer, Cham, 2018; vol. 52, pp. 391–417. https://doi.org/10.1007/978-3-642-40128-2.
